# Producing molecular biology reagents without purification

**DOI:** 10.1371/journal.pone.0252507

**Published:** 2021-06-01

**Authors:** Sanchita Bhadra, Vylan Nguyen, Jose-Angel Torres, Shaunak Kar, Stéphane Fadanka, Chiara Gandini, Harry Akligoh, Inyup Paik, Andre C. Maranhao, Jenny Molloy, Andrew D. Ellington

**Affiliations:** 1 Department of Molecular Biosciences, College of Natural Sciences, The University of Texas at Austin, Austin, Texas, United States of America; 2 Center for Systems and Synthetic Biology, The University of Texas at Austin, Austin, Texas, United States of America; 3 Freshman Research Initiative, DIY Diagnostics Stream, The University of Texas at Austin, Austin, Texas, United States of America; 4 Mboalab Biotech, Yaoundé, Cameroon; 5 Department of Chemical Engineering and Biotechnology, University of Cambridge, Cambridge, United Kingdom; 6 Hive Biolab, Kumasi, Ghana; University of Helsinki, FINLAND

## Abstract

We recently developed ‘cellular’ reagents–lyophilized bacteria overexpressing proteins of interest–that can replace commercial pure enzymes in typical diagnostic and molecular biology reactions. To make cellular reagent technology widely accessible and amenable to local production with minimal instrumentation, we now report a significantly simplified method for preparing cellular reagents that requires only a common bacterial incubator to grow and subsequently dry enzyme-expressing bacteria at 37°C with the aid of inexpensive chemical desiccants. We demonstrate application of such dried cellular reagents in common molecular and synthetic biology processes, such as PCR, qPCR, reverse transcription, isothermal amplification, and Golden Gate DNA assembly, in building easy-to-use testing kits, and in rapid reagent production for meeting extraordinary diagnostic demands such as those being faced in the ongoing SARS-CoV-2 pandemic. Furthermore, we demonstrate feasibility of local production by successfully implementing this minimized procedure and preparing cellular reagents in several countries, including the United Kingdom, Cameroon, and Ghana. Our results demonstrate possibilities for readily scalable local and distributed reagent production, and further instantiate the opportunities available via synthetic biology in general.

## Introduction

High cost of molecular biology reagents, need for preserving their cold chain, and long distance shipping delays and complicated customs regulations often hamper the ability of researchers and students to freely access and adopt molecular and synthetic biology techniques, especially in low resource settings [1, 2]. Complex procedures and infrastructure required for preparing these reagents frequently deters local production. The resulting hindered access to reagents often translates to restricted hands-on learning, a dearth of trained human resource, and inability to meet large scale needs, such as those facing widespread molecular testing during the ongoing COVID-19 pandemic.

Advances from the field of synthetic biology and protein purification have the potential to address some of these needs [3–7]. We previously developed ‘cellular’ reagents–lyophilized bacteria engineered to overexpress proteins of interest–as an easier and cheaper-to-make alternative to commercial enzymes commonly used in diagnostic and molecular and synthetic biology procedures, such as PCR, isothermal amplification, reverse transcription and DNA assembly [8]. Since cellular reagents perform comparably to pure commercial counterparts but are easier to produce and do not require cold chain during storage, they have the potential to significantly reduce cost and infrastructure barriers to molecular/synthetic biology research and education. However, this potential can only be fulfilled if cellular reagents can be freely and affordably acquired. The best way to ensure unfettered access is to locally produce cellular reagents at or near sites of consumption.

While our original method for preparing cellular reagents requires considerably less instrumentation and expertise compared to production of purified enzymes, it is still impractical for most people to adopt because it uses a lyophilizer to dehydrate cellular reagents via sublimation. Lyophilizers are complex, expensive machines that need dependable infrastructure and regular maintenance for operation. Most typical molecular biology laboratories are not equipped with these instruments. Moreover, cellular reagents must be frozen prior to lyophilization, which requires additional infrastructure in the form of either a -80°C freezer or a dry ice or liquid nitrogen vendor. As a result, local production of lyophilized cellular reagents, especially in resource poor settings, remains difficult.

**Fig 1:**
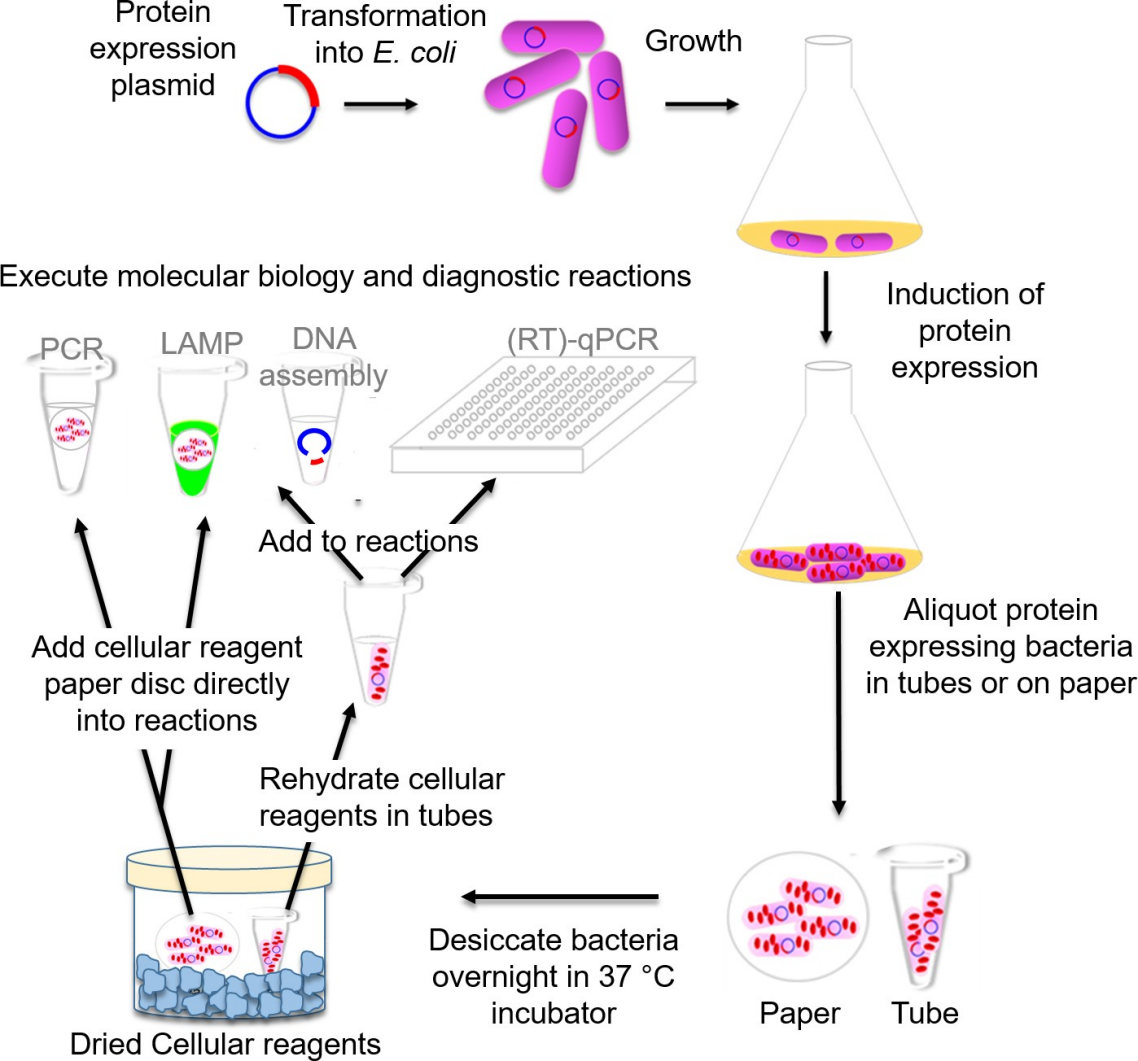
Schematic depicting protocol for preparation and use of evaporated cellular reagents.

We have now overcome this limitation, and demonstrated a considerably easier approach for preparing cellular reagents that requires only a bacterial incubator to grow protein-expressing bacteria and to subsequently dry them into cellular reagents by simply incubating the bacteria at 37°C in the presence of inexpensive chemical desiccants (**Fig 1**). Similar to their lyophilized counterparts, and unlike most commercial preparations of purified enzymes, dried cellular reagents are stable at ambient temperature for several months–a crucial attribute in low resource settings with infrastructure limitations. Furthermore, simple substitution of commercial enzymes with rehydrated cellular reagents allow existing molecular biology protocols to be readily and accurately performed. In fact, by drying cellular reagents on paper discs that can be directly added to molecular reactions, we demonstrate an even more facile reagent-dispensing system that is both easy to manufacture and use. Overall, this method proved suitable for preparation of an expansive set of dried cellular reagents, including DNA polymerases, such as Taq and Bst-LF, reverse transcriptases, such as MMLV and RTX, restriction enzymes, such as *Bsa*I, and ligases, such as T7 DNA ligase. These dried cellular reagents performed comparably to commercial enzymes in common nucleic acid amplification and DNA assembly techniques, including PCR, RT-qPCR, LAMP, and Golden Gate cloning, and enabled assembly of inexpensive and easy to use testing kits. Most importantly, dried cellular reagent technology proved amenable to local implementation in diverse settings. Researchers based in the United Kingdom, Cameroon, and Ghana successfully produced OpenVent DNA polymerase cellular reagents and used them in a sickle cell PCR diagnostics workshops. These results not only demonstrate the possibilities of readily scalable local and distributed reagent production, an important step towards building local scientific capacity [9], but further instantiate the opportunities available via synthetic biology in general.

## Material and methods

### Chemicals and reagents

All chemicals were of analytical grade and were purchased from Sigma-Aldrich (St. Louis, MO, USA) unless otherwise indicated. Bacterial growth media were purchased from Thermo Fisher Scientific (Waltham, MA) and Athena Enzyme Systems (Baltimore, MD, USA). Competent *E*. *coli* protein expression strains, purified enzymes, and related buffers were purchased from New England Biolabs (NEB, Ipswich, MA, USA) unless otherwise indicated. The EndA- RecA- BL21(DE3) *E*. *coli* strain was purchased from EdgeBio (San Jose, CA, USA). KlenTaq1 DNA polymerase was purchased from DNA Polymerase Technologies (St. Louis, MO, USA). All oligonucleotides and gene blocks (**S1 Table in S1 File)** were obtained from Integrated DNA Technologies (IDT, Coralville, IA, USA). SARS-CoV-2 N gene armored RNA was obtained from Asuragen, Austin, TX, USA. SARS-CoV-2 virions were obtained from American Type Culture Collection.

### Plasmids and cloning

Taq, KlenTaq, Bst-LF, T4 DNA ligase, T7 DNA ligase, and MMLV-RT were expressed from the anhydrotetracycline inducible pATetO 6xHis plasmid [8, 10]. RTX expression was driven from a T7 promoter-containing pET vector (Addgene; Plasmid #102787). Br512 was also expressed from a T7 promoter in an in-house *E*. *coli* expression vector, pKAR2 (Addgene; Plasmid #16187). *Geobacillus stearothermophilus* methyltransferase, *Bsm*AIM (UniProtKB—Q6UQ63) was expressed from a constitutive pLac promoter. *Geobacillus stearothermophilus* restriction endonuclease (*Bsa*IR; UniProtKB—Q6SPF4) was expressed from an Isopropyl β- d-1-thiogalactopyranoside (IPTG)-inducible T7 promoter in bacteria expressing *Bsm*AIM. OpenVent, the off-patent DNA polymerase from *Pyrococcus sp*., was cloned in the custom built IPTG-inducible pOBL plasmid. All assembled plasmids were verified by Sanger sequencing. Construction of plasmids is detailed in **Supplementary Materials and methods in S1 File**.

### Production of evaporated cellular reagents

Evaporated cellular reagents were prepared in the following *E*. *coli* strains–Taq, KlenTaq, Bst-LF, and MMLV-RT in BL21; T4 DNA ligase, T7 DNA ligase, *Bsa*IR/*Bsm*AIM, and for some experiments, Bst-LF in the Acella (BL21 DE3 derivative ΔendA ΔrecA) strain; and RTX and Br512 in BL21(DE3). For all single transformants, overnight cultures were grown at 37°C in 3–5 mL Superior broth (Athena Enzyme Systems) containing appropriate antibiotics (100 μg/mL ampicillin). These were used to grow 50 mL 1:200 sub-cultures in Superior Broth with antibiotics in 250 mL conical flasks at 37°C and 225 rpm. Logarithm phase (typical A_600_ = 0.4 to 0.7) cultures were induced as follows– 3 h induction at 37°C with 20 ng/mL anhydrotetracycline (aTC) for Taq, KlenTaq, Bst-LF, MMLV-RT, T4 DNA ligase, and T7 DNA ligase; 16–18 h at 18°C with 1 mM IPTG for RTX; and 18 h at 18°C with 1mM IPTG and 100 ng/mL aTC for Br512. *Bsa*IR/*Bsm*AIM co-transformants were recovered at 37°C for 1 h without antibiotics and then for 1 h with 50 μg/mL kanamycin followed by 18–24 h growth at 30°C on LB agar containing both ampicillin and kanamycin. Individual transformants were cultured for 24 h at 30°C in 3 mL Superior broth containing both antibiotics. Subsequently, 50 mL Superior broth subcultures grown at 30°C till log phase were induced overnight at 16°C with 1 mM IPTG.

Four to six aliquots of 1.2 mL induced cultures were placed in 1.7 mL microcentrifuge tubes. Bacteria were collected by centrifugation at room temperature and 9000 x g for 1 min, washed twice in 1 mL cold 1X PBS (137 mM NaCl, 2.7 mM KCl, 10 mM Na_2_HPO_4_, 1.8 mM KH_2_PO_4_, pH 7.4) and resuspended in cold 1X PBS at a density between A_600_ ~ 6.0–7.0. Some 2 x 10^7^ or 2 x 10^8^ of these induced bacteria (estimated from the A_600_ value using the relation 0.5 optical density = 5x10^8^ bacteria/mL) were then aliquoted into 8 x 0.2 mL microtube strips. In some cases, 2 x 10^7^ bacteria were aliquoted directly onto 3 mm paper discs made of cellulose (CL, Catalog number CFSP173000, Millipore, Burlington, MA, USA), glass fiber (161, Catalog number 1610–0210, Ahlstrom, Finland; 691, Catalog number 28297–976, VWR, Radnor, PA, USA; 693, Catalog number 28297–500, VWR), glass fiber conjugate paper (PTR5, Catalog number R-1805/1683, Advanced Microdevices (Mdi), India; GF800, Catalog number GFCP000800, Sigma-Aldrich), or polyethersulfone (PES, Catalog number GPWP04700, Millipore, Burlington, MA, USA) placed individually inside 0.2 mL microtubes. A single hole was punched in each microtube cap using an 18-gauge needle. Two to three 8-tube strips were then placed inside 118 mL lidded plastic jars filled halfway with calcium sulfate desiccant pellets to facilitate evaporation. In some experiments, evaporation was performed in the absence of any desiccant. After capping, the jars were placed in a small personal-sized 37°C bacterial incubator for 1–2 days, unless otherwise indicated, to allow all the liquid to dry up. In some experiments, the capped jars were placed in a -20°C freezer, in a 4°C refrigerator, or on a 25°C lab countertop for indicated durations. In some cases, cellular reagents were lyophilized as described previously [8], either directly in microtubes or on 3 mm paper discs placed inside 0.2 mL microtubes or stuck on strips of self-adhesive laminating plastic sheet (Fellowes 5221502, Itasca, IL, USA). Once the cellular reagents were visibly dry, the hole in each microtube cap was plugged with hot glue and the desiccant jars with dry cellular reagents were stored in a cabinet at room temperature.

#### Preparation of OpenVent cellular reagents in Ghana and Cameroon

A single BL21(DE3) pOBL1 transformant selected with kanamycin on Luria Bertani (LB: 10 mg/mL tryptone, 5 mg/mL yeast extract and 10 mg/mL NaCl in distilled water) agar and cultured overnight in 5 ml LB broth at 37°C was used to grow a 1:200 sub-culture in 50 mL LB broth supplemented with 100 μg/mL Kanamycin. Manual agitation of the culture flask was performed every 15 min to ensure appropriate aeration of the medium. After ~3 h of incubation, at 0.4 OD_600_ bacterial cell density (evaluated using McFarland turbidity standards; see **Supplementary Material and methods, S1 Fig and S2 Table in S1 File**), the culture was induced with IPTG (1mM final concentration) for four hours with manual agitation every 15 min. The cell density at the time of harvest was around OD_600_ 2. Bacteria were collected by centrifugation, washed, resuspended in 1X PBS, and aliquoted in 8-strips of 0.2 mL PCR tubes as described above. With the lids left opened, the strips were placed in an air-tight container **(S2 Fig in S1 File)** filled halfway with desiccant silica beads (Silica gel, 2–5 mm, Merck CAS-No:7631-86-9) and incubated overnight in a 37°C DIY incubator (**S3 Fig in S1 File**; for assembly instructions see **Supplementary Materials and methods in S1 File**). In some cases, living bacteria were deactivated by incubating the cellular reagents at 50, 60 or 70°C for 10 or 20 minutes prior to drying. The dryness of the sample was inspected by eye and the activity of the cellular reagent was assayed by PCR **(S4 Fig in S1 File)**. To assess the stability over time, dried OpenVent cellular reagents were sealed in plastic bags (Antistat metal shielding ziplock bags, Rapid Electronics Ltd) containing 7g of desiccant silica beads and stored in three conditions: at room temperature, at 4°C and at 37°C. The viability of prepared enzymes was assessed by performing PCR reactions to amplify the 400 bp polymorphic portion of a synthetic human β-globin gene.

### PCR using evaporated cellular reagents

All assay mixes were assembled and kept on ice prior to initiation of amplification. PCR reactions were assembled in 25 μL (CT16S) or 50 μL (TtgR and HF183) volumes containing 10 ng or indicated amounts of plasmid templates along with compatible forward and reverse primers (HF183 assays for colorimetric detection received fluorescein-labeled reverse primers along with 100 nM of biotinylated probe), deoxyribonucleotides, and an indicated enzyme source– 3 μL (2 x 10^7^ cells) of Taq or KlenTaq cellular reagents freshly rehydrated in 30 μL water, one 3 mm diameter paper disc containing 2 x 10^7^ dried Taq or KlenTaq cellular reagents directly added to and kept immersed in the PCR reaction, or pure Taq or KlenTaq DNA polymerase. Endpoint PCRs were analyzed after 20–30 cycles of amplification by agarose gel electrophoresis or by using fluorescein/biotin-specific colorimetric lateral flow dipsticks (Milenia HybriDetect 1, Milenia Biotec GmbH). Quantitative PCRs included 1X EvaGreen intercalating dye (Biotium, Freemont, CA), whose fluorescence was measured using a real-time PCR machine. Additional details of PCR protocols are described in the **Supplementary Materials and methods in S1 File.**

### LAMP-OSD

All assay mixes were assembled and kept on ice prior to initiation of amplification. Human glyceraldehyde-3-phosphate dehydrogenase (*gapd*), HF183, and multiplex SARS-CoV-2 LAMP-OSD reaction mixtures were prepared in 25 μL volumes containing indicated DNA or RNA templates in appropriate reaction buffers supplemented with compatible LAMP primer mixes, deoxyribonucleotides, betaine, MgSO_4_, OSD probes, and one of the following DNA polymerase sources– 16 units of Bst 2.0 (NEB), 3 μL of Bst-LF-expressing BL21 (*gapd*) or Acella (HF183) cellular reagents hydrated in 30 μL water, a 3 mm paper disc containing 2 x 10^7^ evaporated Bst-LF cellular reagents (*gapd*), or 3 μL of Br512-expressing BL21(DE3) cellular reagents rehydrated in 30 μL water and pretreated at 65°C for 30 min prior to use (SARS-CoV-2). *Gapd* LAMP-OSD fluorescence was measured in real-time at 65°C. HF183 assays were incubated at 60°C for 90 min while SARS-CoV-2 assays were kept at 65°C for 1 h followed by 1 min incubation at 95°C. Presence or absence of endpoint OSD fluorescence at room temperature was read visually and imaged using a ChemiDoc camera (Bio-Rad, Hercules, CA, USA). Colorimetric readout was performed using U-Star (Ustar Biotechnologies, Zhejiang, China) or Milenia HybriDetect 1 (Milenia Biotec GmbH) fluorescein/biotin lateral flow dipsticks. Additional details of LAMP-OSD protocols are described in the **Supplementary Materials and methods in S1 File.**

### DNA assembly using cellular reagents

DNA assembly reactions were prepared in 20 μL volume containing 1X T4 DNA ligase buffer (NEB; 50 mM Tris-HCl, 10 mM MgCl_2_, 1 mM ATP, 10 mM DTT, pH 7.5 @ 25°C), 50 ng of plasmid vector bearing two *Bsa*I restriction sites downstream of a constitutive pLac promoter, 110 ng of a PCR amplicon encoding the pink chromoprotein FP595 [11] flanked by two *Bsa*I restriction sites (**S5 Fig in S1 File**), and an enzyme source– 400 units of pure T4 DNA ligase (NEB), 20 units of pure *Bsa*I-HF-V2 (NEB), a combination of both pure enzymes, 2 x 10^7^ *Bsa*I cellular reagents, 2 x10^7^ T4 DNA ligase cellular reagents, 2 x 10^7^ T7 DNA ligase cellular reagents, or a combination of 2 x 10^7^ each of *Bsa*I and either T4 or T7 DNA ligase cellular reagents. All cellular reagents were hydrated in water immediately prior to use. All reactions were subjected to 15 cycles of 5 min at 37°C followed by 5 min at 16°C, a final 20 min at 65°C, and transformation of 1 μL reaction into 50 μL of chemically competent Top10 *E*. *coli*. Transformants were recovered for 1 h in a 37°C shaker and then grown overnight on Superior broth agar plates containing chloramphenicol. Individual colonies were inoculated in 1 mL superior broth with chloramphenicol and grown overnight in 96-well grow blocks incubated in a 37°C shaker. Bacterial pellets were collected by centrifuging at 3000 x g for 10 min at 4°C, imaged using a cellphone camera, and extracted for plasmids that were Sanger sequenced using vector-specific primers flanking the insert.

## Results

### Transport and use of lyophilized cellular reagents in Africa

Previously we reported the development and in-house validation of lyophilized cells as reagents for a variety of molecular biology and molecular diagnostics applications, such as the polymerase chain reaction (PCR), reverse transcription (RT) PCR, and loop-mediated isothermal amplification (LAMP) [8]. We also demonstrated that cellular reagents could be used to fabricate DNA constructs via the Gibson assembly [12] of as many as three separate DNA parts. The lyophilized cellular reagents proved stable at ambient temperature for extended periods, and therefore potentially provided ready access to reagents for researchers in resource poor settings.

To proof this conjecture, we sent ready-to-use lyophilized cellular reagents for Taq DNA polymerase to our collaborators at the University of Cambridge (UK), who in turn traveled to the Mboalab in Yaoundé, Cameroon and the Hive Biolab in Kumasi, Ghana. The cellular reagents travelled for a total of 2 weeks, by FEDEX courier, and then in a luggage to the final destination. During this time the reagents were kept in a closed container in the presence of desiccant (Drierite, W. A. Hammond DRIERITE Co. LTD, Xenia, OH, USA) and the storage temperature was uncontrolled. In both the Mboalab and the Hive Biolab, researchers were able to successfully use cellular reagents in a molecular biology workshop in place of commercial pure polymerases for demonstration of a molecular diagnostics workflow such as PCR amplification of the polymorphic portion of a synthetic human β-globin gene containing sickle cell disease variants (Sickle Cell Genetics Lab: Diagnosing Baby Marie™, MiniPCR bio, Cambridge, MA, USA, **S6A Fig in S1 File)**.

The cellular reagents were then tested for stability while stored in an air-tight container, in the presence of desiccant, at 4°C. Stability tests were performed with the synthetic β-globin gene in the following months and revealed that the freeze-dried Taq DNA polymerase maintained its activity for at least 11 months (**S6B Fig in S1 File**). It is worth noting that in both locations hours of electricity blackouts were routine, preventing any continuous cold chain.

### Simplifying cellular reagent production by eliminating the lyophilization step

While lyophilized cellular reagents could be shipped, this was still a limitation for scaling local applications, as lyophilizers are often not available in resource-poor settings. We therefore sought to determine whether an even simpler method, evaporation, could be used for the dehydration and storage of protein-expressing cellular reagents. We hypothesized that since many prokaryotes are tolerant to desiccation and evaporation is commonly used to dehydrate and preserve bacteria (e.g., probiotic preparations), it might also be suitable for drying cellular reagents while preserving their functionality [13].

In particular, we wished to create an entirely new reagent-dispensing system based on paper disks that could be used in molecular reactions, such as PCR, by simply immersing the disk in the reaction mix. To this end, we suspended Taq DNA polymerase-expressing *E*. *coli* bacteria in 1X PBS and distributed 3 μL (2 x 10^7^ CFU) of this suspension into four sets of 0.2 mL PCR tubes whose lids had been punched with an 18-gauge needle to make a single hole. We had previously observed that cellular reagents could be lyophilized on different types of paper discs or on laminated plastic, including glass fiber conjugate paper (**S7 Fig in S1 File**). Therefore, some tubes were loaded with the cellular reagent suspension directly, while the remaining ones were pre-loaded with 3 mm discs of varying glass fiber papers to soak up the 3 μL liquid and thereby provide larger surface area for expedited evaporation.

We placed the sets of tubes in 118 mL lidded plastic jars filled halfway with calcium sulfate desiccant pellets to facilitate evaporation. We then carried out overnight evaporation at four different temperatures, incubating the jars in a -20 ˚C freezer, a 4 ˚C refrigerator, a 25 ˚C countertop, or a small personal-sized 37 ˚C bacterial incubator. Lyophilized cellular reagents served as positive controls for these preparation methods. Cellular reagents were completely dry after overnight incubation at 25 ˚C or 37 ˚C. After 4 days, cellular reagents kept at 4 ˚C were fully dry when visually inspected, but cellular reagents at -20 ˚C still retained moisture.

**Fig 2:**
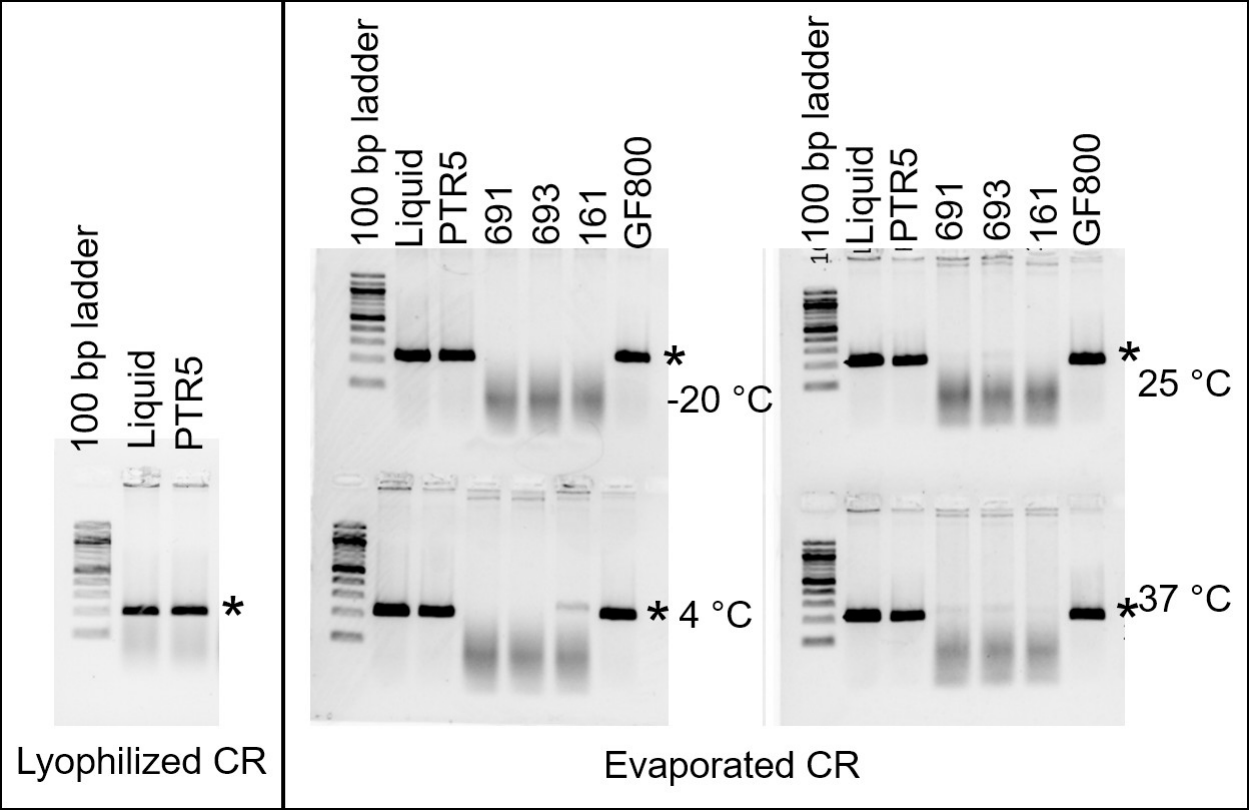
Comparison of lyophilized and evaporated Taq DNA polymerase cellular reagents prepared with or without solid support. Activities of cellular reagents were compared by performing 20 cycle PCR amplification of 10 ng CT16S plasmid templates using either 3 μL of rehydrated evaporated or lyophilized cellular reagents prepared without solid support (‘Liquid’) or a single 3 mm paper disc containing evaporated cellular reagents added into the PCR reaction mix (PTR5 and GF800: two types of glass fiber conjugate pad paper; 691, 693, 161: three types of glass fiber filter paper). Agarose gel electrophoretic analysis of resulting PCR amplicons is depicted. Expected product size is indicated by an asterisk. Data shown are representative of eight biological replicates.

We compared the activity of the evaporated versus lyophilized cellular reagents by performing PCR amplifications of a standard DNA template (**S1 Table in S1 File**) followed by gel electrophoretic analysis of the PCR amplicons. We observed that cellular reagents completely dried by evaporation either directly in tubes or on PTR5 and GF800 glass fiber conjugate papers at 4 ˚C, 25 ˚C, or 37 ˚C generated a single prominent PCR amplicon of the expected size in amounts comparable to those generated by cellular reagents lyophilized directly in tubes or on PTR5 paper discs (**Fig 2**). Cellular reagents that had been kept at -20°C also performed well in PCR. In contrast, cellular reagents evaporated on 691, 693, and 161 glass fiber paper matrices were poorly- or non-functional. Overall, these results suggested that it was possible to prepare paper-based cellular reagents simply by evaporation and directly add them to reactions.

To confirm and extend these findings, we prepared new batches of evaporated cellular reagents for Taq DNA polymerase as well as for two additional DNA polymerases–KlenTaq and Bst-LF. All three cellular reagents were evaporated either directly in tubes or on GF800 glass fiber conjugate paper discs. We used the GF800 paper matrix as a solid support because it was more absorptive and sturdier than PTR5 paper. The reagents were allowed to evaporate inside lidded jars containing desiccant pellets for 4 days at -20°C or 4 ˚C or for overnight at 25 ˚C or 37 ˚C. The jars of dried reagents from 4 ˚C, 25 ˚C and 37 ˚C and the slightly wet reagents from -20°C were then kept in a dark cabinet at room temperature for 2–2.5 months prior to functional testing.

**Fig 3:**
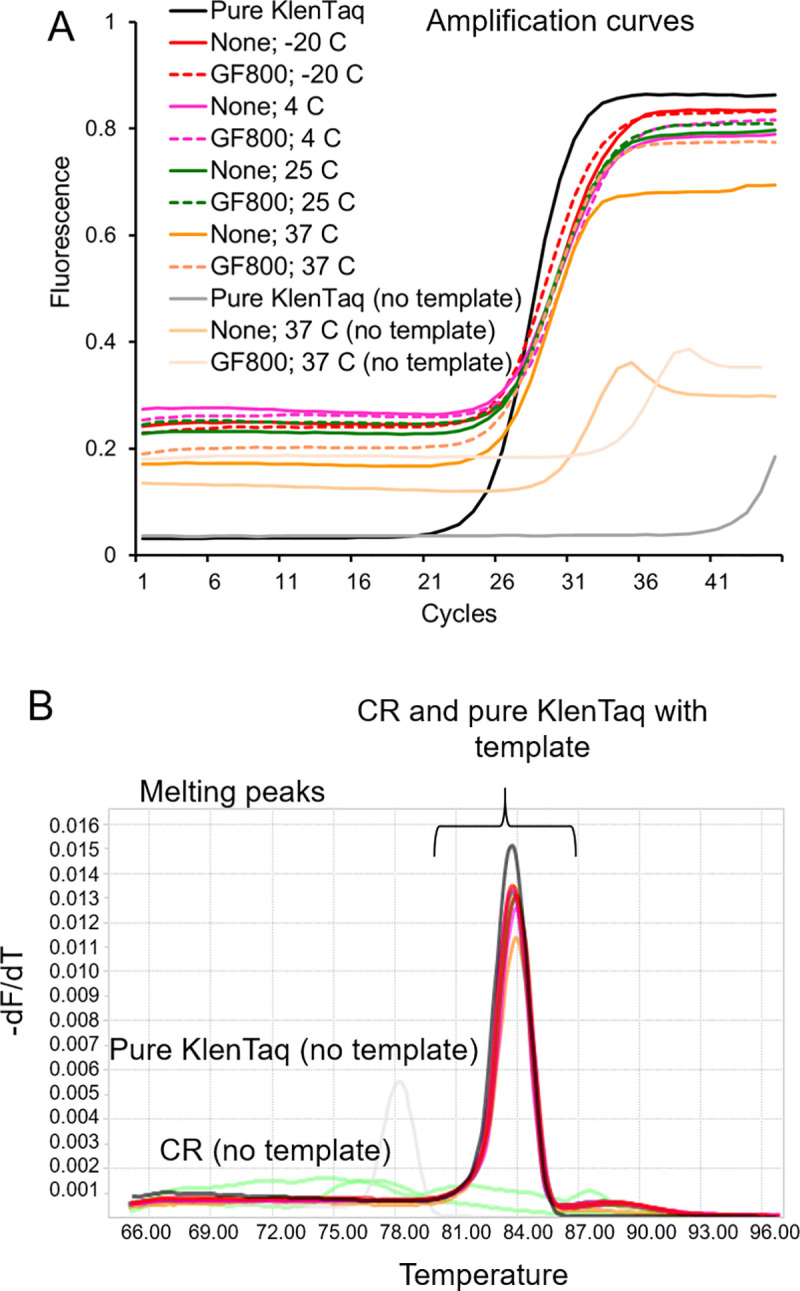
Activity of evaporated KlenTaq cellular reagents. KlenTaq DNA polymerase cellular reagents evaporated at the indicated temperature with (GF800) or without (None) solid support were tested after 2 months of preparation by performing qPCR reactions with or without CT16S plasmid templates. Quantitative PCR reactions performed using commercially obtained pure KlenTaq enzyme served as control. Amplification curves observed in real-time by measuring increase in fluorescence of intercalating EvaGreen dye are depicted in panel A. Melting temperature analysis of resulting PCR amplicons is shown in panel B. Data shown are representative of three biological replicates.

Even after extended storage at ambient temperatures, all preparations of evaporated Taq and KlenTaq DNA polymerase cellular reagents (including the initially wet -20°C reagents that subsequently dried during storage), were active and yielded the expected PCR amplicon when rehydrated in assay buffer and templates (**S8 Fig in S1 File**). Real-time PCR amplification kinetics measured by EvaGreen dye intercalation revealed that evaporated KlenTaq cellular reagents produced a similar time-to-signal and a product with similar melting temperatures when compared to the fresh, pure enzyme (**Fig 3**). A two-way ANOVA test of Cq values did not show statistically significant effect of temperature or drying format (direct versus on paper) on performance of cellular reagents (P-value = 0.996).

**Fig 4:**
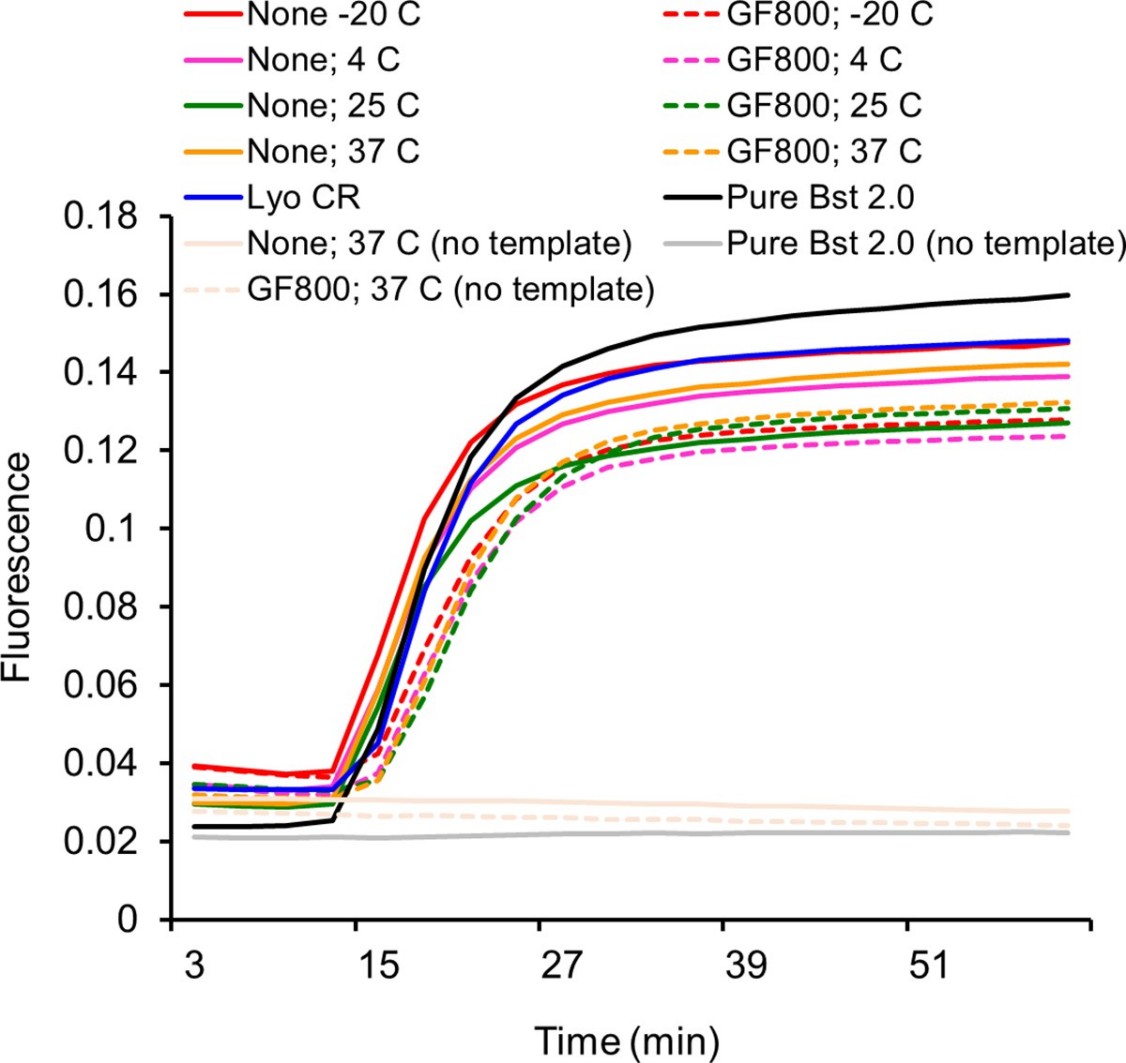
Activity of Bst-LF evaporated cellular reagents. Bst-LF DNA polymerase cellular reagents evaporated at the indicated temperature with (GF800) or without (None) solid support were tested after 2 months of preparation by performing LAMP-OSD reactions with or without human gapd plasmid DNA templates. LAMP-OSD reactions performed using commercially obtained pure Bst 2.0 enzyme or with lyophilized Bst-LF cellular reagents (Lyo CR) served as controls. Amplification curves observed in real-time by measuring increase in fluorescence of OSD probes are depicted. Data shown are representative of three biological replicates.

The direct and on-paper versions of evaporated Bst-LF cellular reagents were also active and demonstrated similar performance to commercial Bst 2.0 enzyme (New England Biolabs), an engineered improvement of Bst-LF, in loop-mediated isothermal amplification (LAMP) of a DNA template followed by signaling via oligonucleotide strand displacement (OSD) probes (**Fig 4**) [14, 15]. A two-way ANOVA test of time-to-signal did not reveal statistically significant effect of temperature or drying format (direct versus on paper) on performance of Bst-LF cellular reagents (P-value = 0.961).

To confirm that evaporation can be universally used to prepare cellular reagents, even for non-thermostable proteins, we prepared direct and on-paper versions of Molony murine leukemia virus reverse transcriptase (MMLV RT) by overnight evaporation at 37°C. When tested in TaqMan reverse transcription qPCR assays, both types of evaporated MMLV RT cellular reagents decreased the Cq of amplification by ~8-fold when compared to assays performed without any added reverse transcriptase (**Supplementary Materials and methods** and **S9 Fig in S1 File**). These results suggest that cellular reagents are an effective delivery system not only for thermostable enzymes but also for mesophilic reagents. This is likely because desiccation and drying in the absence of external protectants, such as trehalose, starch, or skimmed milk, render bacterial membranes leaky, especially upon rehydration [13, 16]. Furthermore, we previously found that cellular reagents dried in the absence of protectants and in the presence of phosphate buffered saline appeared hollow upon Gram staining and disintegrated readily when heated [8]. These observations suggest that cellular reagents may have porous walls that allow mingling of cellular and external reaction components upon rehydration; for reactions carried out at higher temperatures, cells would also undergo further thermal lysis [17].

Overall, these results strongly support that evaporation without a cold chain can be used for the local preparation of cellular reagents for almost any enzyme. Indeed, cellular reagents evaporated without aid of desiccants are also active, albeit to a lesser degree compared to reagents dried in the presence of desiccants (**S10 Fig in S1 File**).

### Preparation of evaporated cellular reagents for international distribution and for local production in Africa

In order to appropriately distribute the evaporated cellular reagents to other laboratories they should not contain living *E*. *coli*, as these would be classified as Genetically Modified Organisms (GMOs). This is particularly important in an international context as many countries, including the UK, require all labs working with GMOs to undertake a form of registration and adhere to additional guidelines compared to labs undertaking only in vitro assays such as PCR. The ability to deactivate cells while maintaining enzymatic activity is therefore crucial for safe and regulated distribution. Unlike the freeze-dried preparations of cellular reagents that do not retain viability due to lack of excipients (such as trehalose) during lyophilization [2], the evaporated cellular reagents retained viable cells for 5 months. To further deactivate the evaporated cellular reagents, a heat treatment was added prior to the evaporation step. As shown in **S4 Fig in S1 File**, a heat treatment for 10 minutes at 60°C was enough to completely kill the cells while nonetheless maintaining DNA polymerase activity.

The new preparation method also enables local manufacture that should conform to a variety of regulatory structures. In collaboration with partners in both Mboalab (Cameroon) and Hive Biolab (Ghana), we attempted to manufacture a DNA polymerase cellular reagent that should have improved characteristics relative to Taq DNA polymerase. The DeepVent™ polymerase is an off-patent DNA polymerase sold by New England Biolabs which has 5 times higher fidelity, higher thermostability (with a half-life of 23h at 95°C) and better performance with difficult templates (such as GC-rich sequences) relative to Taq DNA Polymerase. To enable transfer of materials to Cameroon and Ghana under the Open Material Transfer Agreement [18], we built pOBL1, an open source pUC-based expression vector which carries the gene to express DeepVent™ (renamed and hereafter called OpenVent) under a T7 IPTG-inducible promoter (**S11 Fig in S1 File**). pOBL1 was then constructed, sequenced and transformed into the BL21(DE3) *E*. *coli* strain by the Cambridge research team, emphasizing the possibilities for open source engineering, and then shipped to Hive Biolab and Mboalab.

**Fig 5:**
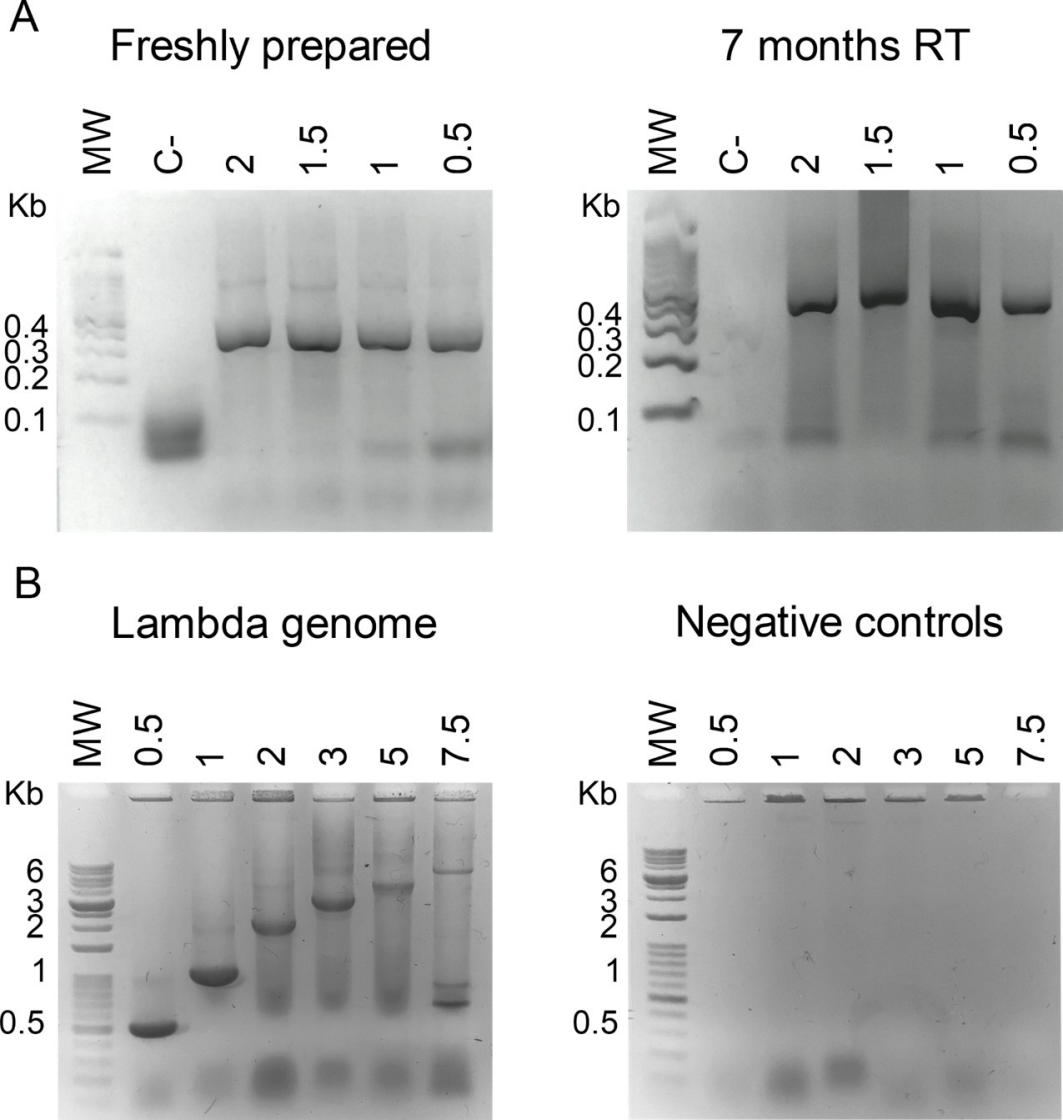
Activity and stability assays of evaporated OpenVent cellular reagents produced in Ghana and Cameroon. (A) Left panel: OpenVent evaporated cellular reagents were used to amplify the synthetic beta-globin human gene. OpenVent is functional down to 25% of the standard concentration (2μL of reagent in 20μL PCR reaction). Right panel: OpenVent evaporated cellular reagents are stable for at least 7 months stored at 4°C in an airtight container in the presence of silica desiccant beads. (B) OpenVent evaporated cellular reagents amplify DNA fragments up to 7.5 kb from lambda genome. The shorter amplicons seen along with the 7.5 kb PCR product is due to unintended mispriming of lambda DNA. No amplicons were observed in the negative controls lacking lambda DNA templates. MW: molecular weight (A: 100bp DNA ladder, Newmarket Scientific, B: 1Kb plus DNA ladder, Beneficial Bio; C-: negative control).

At the time of the OpenVent cellular reagents production trials, both laboratories lacked a photometer and a shaker incubator. Therefore, a DIY incubator was built from locally sourced materials (**S3 Fig in S1 File**) and culture density was assessed using McFarland standards [19–21] **(Supplementary Material and methods, S2 Table and S1 Fig in S1 File)**. The OpenVent expression strain was incubated at 37°C in the DIY incubator and shaken manually every 15 minutes. IPTG induction was initiated at around 0.4 OD_600_, and the culture was harvested after 4 hours of expression. The cellular reagents were aliquoted in PCR tubes, placed with open lids in an air-tight container half-filled with silica gel desiccant, and evaporated overnight at 37°C (**S2 Fig in S1 File**). OpenVent activity was then tested using the β-globin synthetic gene as a template. The OpenVent preparation was not only active for at least 7 months at 4°C, but its concentration could be decreased by a factor of four from a previous protocol developed for Taq DNA polymerase cellular reagents [8] and still successfully amplify a target template (**Fig 5A**). Moreover, the evaporated OpenVent cellular reagents were able to amplify up to 7.5 kb amplicons, starting with a complex template, the bacteriophage lambda genome (**Fig 5B**).

### Rapid response to diagnostic needs using evaporated cellular reagents

By reducing the time and instrumentation needed for reagent production, evaporated cellular reagents can considerably reduce response times when there are emergent reagent needs, such as those encountered in the current COVID-19 pandemic. In this regard, we have previously developed a thermostable reverse transcriptase / DNA polymerase, RTX, that can support one-enzyme dye-read RT-qPCR assays [22, 23], and a novel *Bst* DNA polymerase variant, Br512, with improved capabilities for isothermal amplification assays [10]. We wished to determine whether these enzymes could be dried for rapid distribution for the execution of SARS-CoV-2 specific RT-qPCR and RT-LAMP assays.

To proof RTX rapid response reagents, we set up CDC SARS-CoV-2 N1 RT-qPCR assays (https://www.cdc.gov/coronavirus/2019-ncov/lab/rt-pcr-panel-primer-probes.html) using evaporated RTX cellular reagents and seeded them with different amounts of armored SARS-CoV-2 N gene RNA (Asuragen, Austin, TX, USA). The N1 assay amplifies a 72 nt long region from position 28287 to 28358 of the SARS-CoV-2 viral genomic RNA (GenBank Accession No. MN985325.1). Real-time measurement of amplification kinetics using EvaGreen intercalating dye followed by melt curve analysis of resulting amplicons revealed that the RTX cellular reagent operated N1 RT-qPCR assays could specifically identify as few as 5000 copies of SARS-CoV-2 RNA and generated amplicons with distinct melting temperature (**Supplementary Materials and methods** and **S12 Fig in S1 File**).

**Fig 6:**
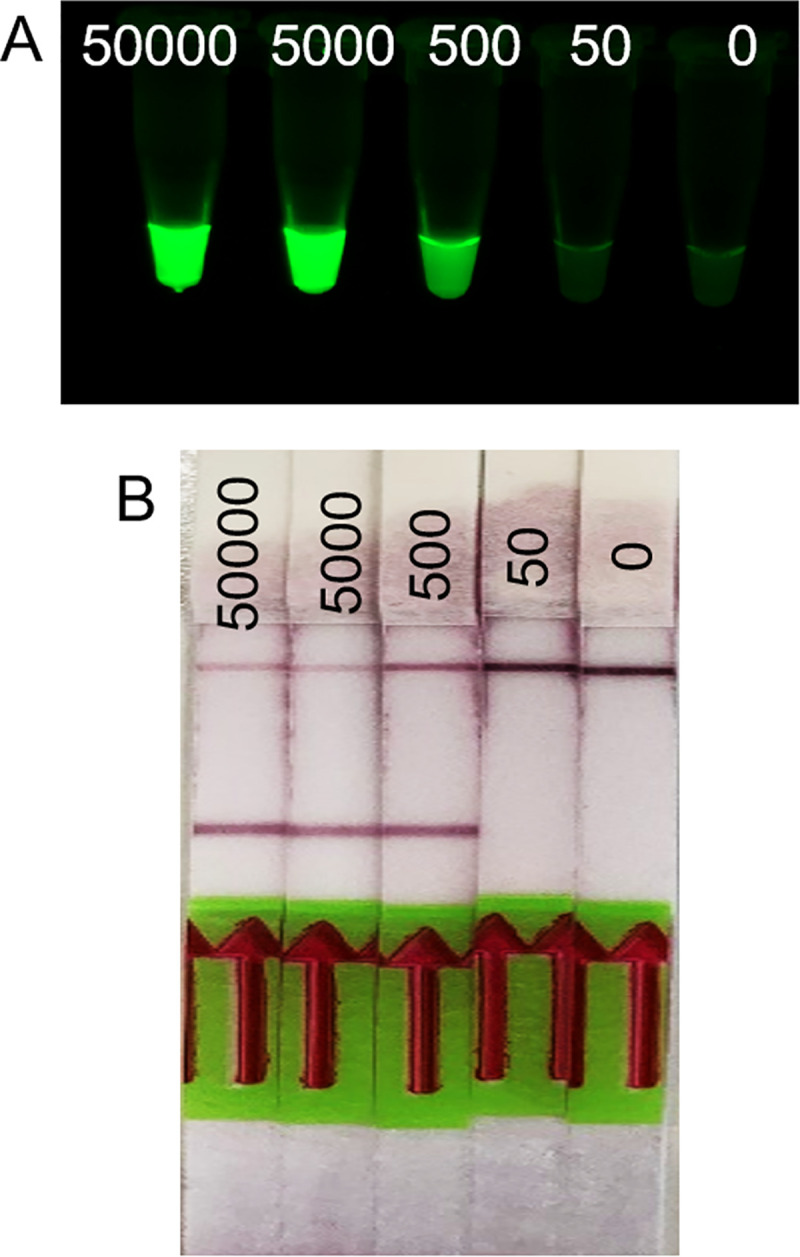
Detection of SARS-CoV-2 using Br512 polymerase evaporated cellular reagents and multiplex RT-LAMP-OSD assays. Multiplex RT-LAMP-OSD assays for viral N and ORF1AB genes were operated using only Br512 polymerase cellular reagents and indicated amounts of inactivated SARS-CoV-2 virions. Images of endpoint OSD fluorescence representative of five biological replicates (A) and colorimetric readout using fluorescein/biotin lateral flow dipsticks representative of three biological replicates (B) are depicted.

We similarly set up a SARS-CoV-2 isothermal amplification assay using Br512 cellular reagents in a multiplex RT-LAMP-OSD assay that amplifies regions in viral N and ORF1AB genes, resulting in strand displacement of amplicon-specific fluorogenic OSD reporters ([24–26]). The ensuing fluorescence in this assay can be observed directly or it can be converted to a colorimetric signal by using biotinylated primers and fluorescein/biotin-specific lateral flow dipsticks. When seeded with as few as 500 SARS-CoV-2 virions, assays with Br512 as a cellular reagent generated visible endpoint fluorescence as well as red test lines on lateral flow dipsticks indicative of successful viral detection (**Fig 6**). Assays lacking specific analytes remained dark and did not lead to color accumulation at test lines of lateral flow strips.

While these detection limits are higher than those that have been achieved with either pure RTX [23] or Br512 RT-LAMP-OSD assays that also contained a dedicated reverse transcriptase [10], they nonetheless show that cellular reagents can potentially allow rapid scale-up in a variety of locales, especially during crises when such rapid testing may be crucial for containing viral spread [27].

### Evaporated cellular reagents for Golden Gate DNA assembly

Having successfully developed a method to produce cellular reagents with only a bacterial incubator/shaker, microcentrifuge, and desiccant, we sought to expand the evaporated cellular reagent toolset to encompass enzymes used in DNA assembly technologies, thereby further enabling local biotechnology approaches [28] in resource-poor settings. We have previously demonstrated that Gibson assembly is possible using cellular reagents and now sought to extend that demonstration to Golden Gate cloning [29], which is far more compatible with parts kits and approaches that can be utilized in a modular and scalable way throughout the world. In particular, we sought to express *Bsa*I restriction endonuclease and T7 DNA ligase as evaporated cellular reagents and demonstrate basal Golden Gate DNA assembly of plasmid constructs from individual parts.

Evaporated cellular reagents were prepared in an endA-, recA- BL21(DE3) strain of *E*. *coli*. The *BsmA*IM methyl transferase was expressed from an *E*. *coli* promoter, and the *Bsa*I restriction endonuclease was inducibly expressed from a T7 promoter in bacteria co-transformed with both expression plasmids. Despite the co-presence of the methyl transferase, the *Bsa*I cellular reagents could cut added plasmid DNA bearing two *Bsa*I sites to release a 745 bp long superfolder GFP-encoding insert, albeit less efficiently than a commercial pure engineered version of the enzyme termed *Bsa*I-HF-V2 (**S13 Fig in S1 File**).

**Fig 7:**
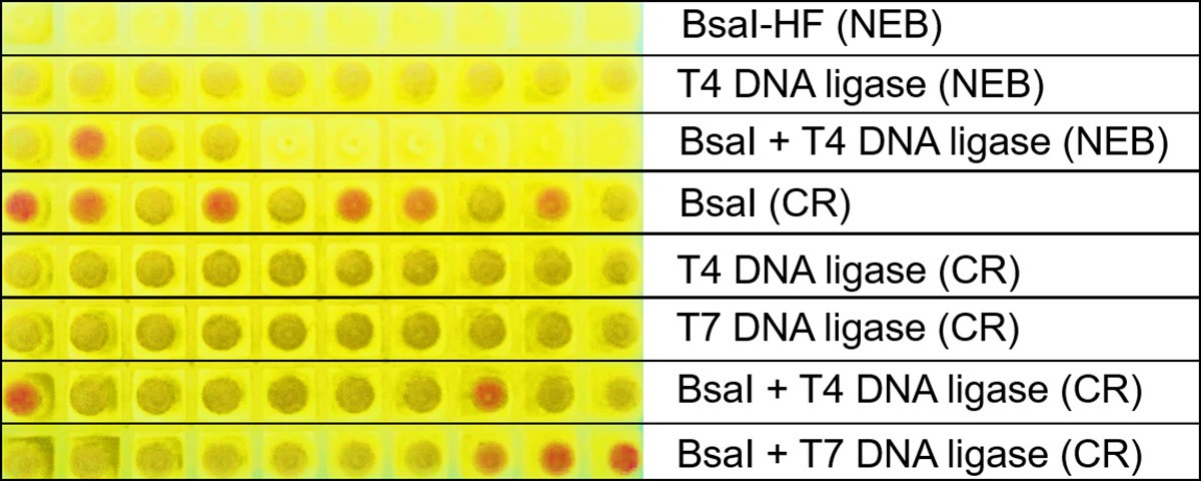
DNA assembly using evaporated cellular reagents. Pure enzymes (NEB) or evaporated cellular reagents (CR) for *Bsa*I, T7 DNA ligase, and T4 DNA ligase were used to assemble a PCR product comprising the coding sequence of pink chromoprotein FP595 flanked by two *Bsa*I restriction sites into a chloramphenicol resistant plasmid bearing two *Bsa*I restriction sites downstream of a constitutive lac promoter. Cellphone image of pellets of individual FP595-expressing (pink) and non-expressing (white) transformants grown overnight in chloramphenicol-containing growth media is depicted. Data are representative of four biological replicates.

To evaluate the feasibility of Golden gate DNA assembly using cellular reagents, some 2 x 10^7^ of *Bsa*I-expressing, evaporated cells were rehydrated and added to a mixture of the vector (a protein expression plasmid bearing two *Bsa*I recognition sites downstream of a constitutive Lac promoter) and the insert (a PCR amplicon encoding the pink chromoprotein, FP595 [11], flanked by *Bsa*I recognition sites) (**S5 Fig in S1 File**). The restriction enzyme cellular reagents were also sometimes supplemented with cellular reagents for T4 DNA ligase or T7 DNA ligase, while control reactions received only T4 or T7 ligase cellular reagents. Following restriction digestion and DNA ligation, vector insert mixtures treated with only *Bsa*I cellular reagents, or with both *Bsa*I and ligase cellular reagents, yielded pink-colored transformants indicative of successful Golden Gate assembly of the FP595 chromoprotein gene into the protein expression plasmid (**Fig 7**). We further confirmed that the FP595 coding sequence had been accurately inserted via Sanger sequencing. Assemblies performed using only *Bsa*I cellular reagents tended to yield more correct clones compared to reactions containing 3 μL each of two types of cellular reagents (*Bsa*I + T4 ligase or *Bsa*I + T7 ligase) suggesting that presence of too much cellular material might hinder accurate assembly perhaps due to crowding and/or activity of nucleases. We had observed a similar caveat during our previous work with Gibson assembly using cellular reagents [8]. The absence of pink colonies from vectors treated with pure *Bsa*I-HF-V2 enzyme suggested that DNA assembly with cellular reagents containing only restriction enzymes might be due to the activity of endogenous *E*. *coli* DNA ligases. Further optimization of assembly conditions and perhaps co-expression of *Bsa*I and ligase in the same cellular reagent strain may improve assembly.

### Using evaporated cellular reagents to build molecular testing kits

**Fig 8:**
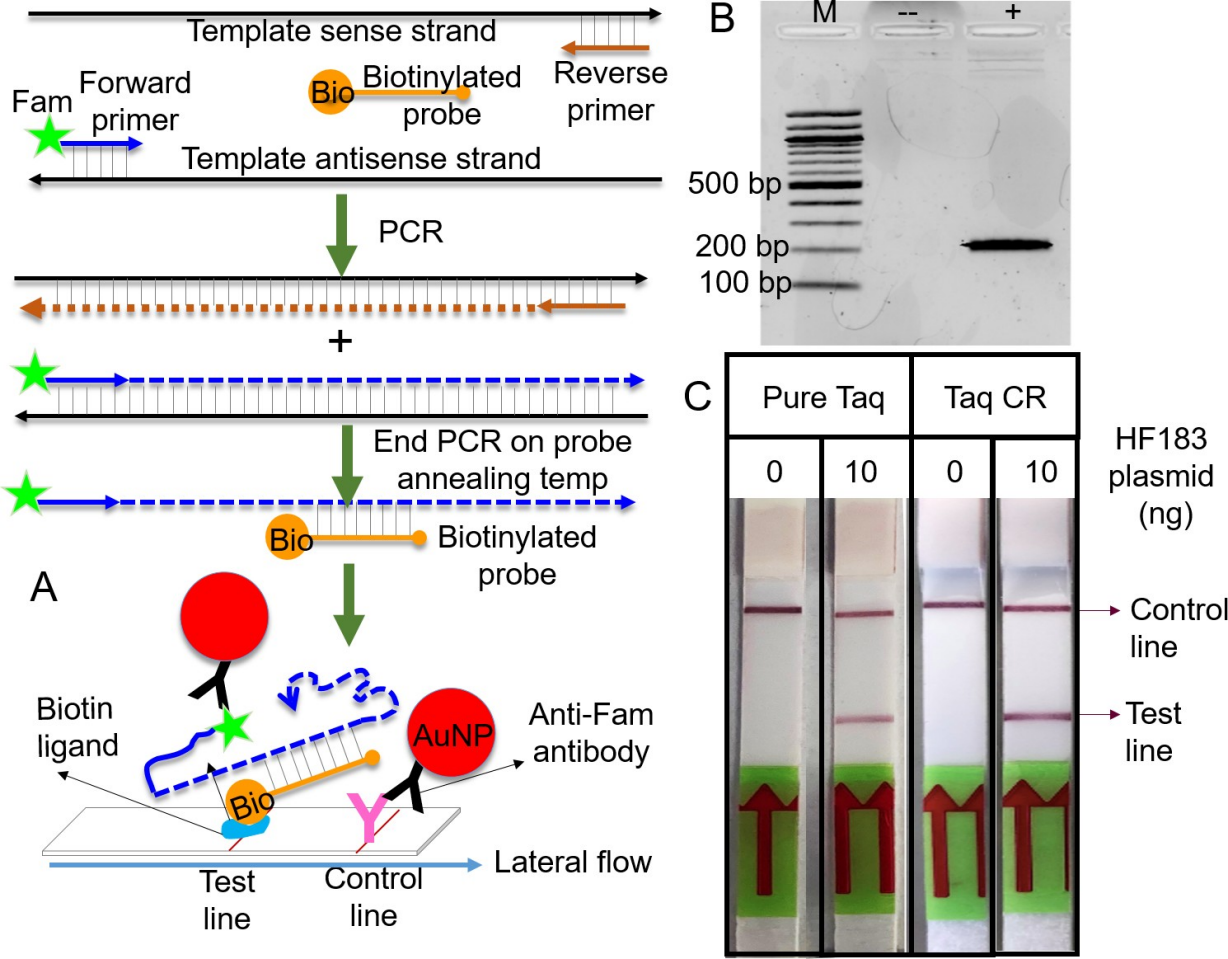
PCR-based molecular testing kit using Taq DNA polymerase evaporated cellular reagents. (A) Schematic depicting design of probe-based colorimetric detection of PCR using fluorescein/biotin-specific lateral flow dipsticks. (B) HF183 PCR performed using evaporated Taq cellular reagents and analyzed by agarose gel electrophoresis. M: 100 bp molecular weight ladder; (-): no template; (+): with 10 ng plasmid template. (C) Indicated amounts of HF183 plasmid templates were PCR amplified using pure Taq DNA polymerase or Taq DNA polymerase evaporated cellular reagents and analyzed using lateral flow dipsticks. Cellphone images of the dipsticks taken after development of red colored control and test lines are depicted. Data are representative of three biological replicates.

The ease of production and opportunities for portability without a cold chain make evaporated cellular reagents attractive candidates for assembling molecular testing kits. As a proof, we sought to use evaporated cellular reagents to build prototype kits for PCR and LAMP, two common nucleic acid amplification methods. Two criteria were invoked in the design of these prototype test kits–first, that they should address real-world issues; and second, they should require minimum ancillary instruments. To address the first criteria, we built a kit around the human-associated *Bacteroides* HF183 marker, a globally-validated diagnostic target for monitoring human fecal contamination in environmental and drinking water [30–33]. Our PCR testing kit employs evaporated Taq DNA polymerase cellular reagents and is based on the TaqMan qPCR assay approved for HF183 detection by the US Environmental Protection Agency. With respect to the second criteria, since qPCR machines are expensive, we reengineered this assay for qualitative endpoint readout using colorimetric lateral flow dipsticks (**Fig 8**) thereby allowing the kits to be used with inexpensive thermocyclers, such as the miniPCR (miniPCR bio, Cambridge, MA, USA), and completely replacing more complex readout instruments, such as agarose gel electrophoresis apparatus or fluorimeters.

We enabled dipstick-based readout of PCR by making two changes to the HF183 TaqMan qPCR assay–first, we replaced the TaqMan probe with a shorter probe labeled with biotin and second, we labeled the reverse primer (that is extended by the DNA polymerase to form the strand complementary to the probe) with a fluorescein moiety (**S1 Table in S1 File**). The biotinylated probe was also engineered to avoid hybridization to amplicons during primer annealing and extension, thus preventing its degradation by Taq DNA polymerase. Instead, we programmed the thermocycler with a final step of heat denaturation and cooling, denaturing the amplicons and allowing probe-amplicon hybridization. The amplicon-probe complexes should juxtapose fluorescein and biotin moieties and allow their colorimetric detection using commercially available fluorescein/biotin-specific lateral flow dipsticks (**Fig 8A**). In the absence of target templates, probe-amplicon complexes should not form and no detectable signal should be generated.

The kit was tested with both synthetic HF183 amplified with pure enzymes, and similar reactions that utilized evaporated Taq DNA polymerase cellular reagents. The PCR products were analyzed both by conventional agarose gel electrophoresis and by lateral flow dipsticks. Agarose gel analysis revealed that, as expected, Taq DNA polymerase cellular reagents generated the correct-sized PCR product only in the presence of HF183 templates (**Fig 8B**). This result was accurately mirrored by dipstick readouts, as evident from the red colored test line only when PCR reactions had received HF183 templates (**Fig 8C**).

We further simplified the opportunities for testing kits via LAMP-OSD, which has no need of thermocycling equipment. We have previously reported on a visually-read isothermal LAMP-OSD assay that targets the same HF183 region as the qPCR test, but needs only a single temperature for amplification, which can be provided with a simple heat block, water bath, or even chemical hand warmers [34]. If correct amplicons are produced, the OSD probes generate high amplitude fluorescence that can be excited using inexpensive LEDs and observed visually with a cellphone camera and simple gel lighting filters (Reference [34] and **S14 Fig in S1 File**). Alternately, by including a biotinylated LAMP primer along with the fluorescein-labeled OSD probe a positive signal can be read by simply applying the reaction to anti-fluorescein/biotin dipsticks [35], similar to what was done with PCR products.

**Fig 9:**
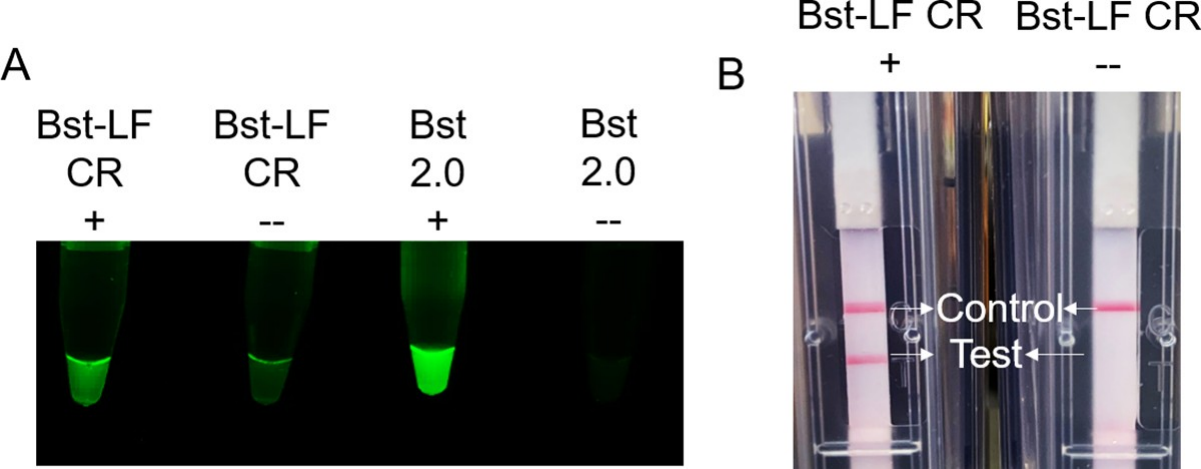
LAMP-OSD based molecular testing kit using Bst-LF evaporated cellular reagents. 80,000 (+) or 0 (—) copies of HF183 plasmid templates were amplified using HF183 LAMP-OSD assays operated with either Bst-LF cellular reagents (CR) or with pure Bst 2.0 enzymes. Endpoint image of OSD fluorescence (A) and colorimetric readout on lateral flow devices (B) are depicted. Data are representative of three biological replicates.

To test the LAMP-OSD kit, reactions were performed using synthetic HF183 targets and evaporated Bst-LF cellular reagents prepared in a BL21(DE3) endA-, recA- strain. As shown in **Fig 9**, in the presence of HF183 templates, the LAMP-OSD cellular reagent kit generated a bright visual fluorescence signal and a red test line on the dipstick. In the absence of specific templates, both visual fluorescence and colorimetric signals remained at background level.

## Discussion

We have demonstrated an uncomplicated method that requires only a 37°C bacterial incubator and a tabletop microcentrifuge to prepare functional enzymes in evaporated cellular reagents. The evaporated reagents are stable for long periods (many months) without a cold chain. The method we have developed is broadly applicable and yielded an extensive cellular reagent toolkit that could be used to execute a variety of molecular biology, diagnostic, and synthetic biology protocols, such as PCR, isothermal amplification, and DNA assembly. Moreover, usage in most cases simply required substitution of pure enzymes with evaporated cellular reagents in otherwise standard protocols. To make this system even more user friendly and implementable, it might be useful to explore cellular reagent production with protein expression systems that do not require antibiotic selection and/or employ cheaper or easier induction mechanisms [36, 37]. Additionally, expansion of this technology to other types of cells (other prokaryotes, such as *Bacillus spp* as well as eukaryotes, such as *Saccharomyces*), could expand the range, type, and compatibility of delivered reagents.

The development of evaporated cellular reagents has parallels with a trend towards cell free biology that has also proven that biological activity can be preserved in dried crude cell extracts, which is extremely enabling for research [38], diagnostics [39, 40], and teaching [4]. The ability to prepare and use cell-free extracts has allowed the construction of genetic circuitry that can function wholly *in vitro*, with individual components being transcribed and translated as needed. For example, cell-free biosynthetic systems can be used to program the introduction of non-canonical amino acid analogs and genetic code modifications much more readily than engineered cells [41, 42]. Cell-free biosensor circuits have been used to measure small molecule and metal contaminants in water, ultimately transcribing easily read fluorescent reporters [39]. Evaporated cellular reagents could potentially be substituted for many of these applications. Elements from the new cellular reagent manufacturing process may also be adaptable to produce cell extracts at lower cost and with wider diversity of use.

The minimal requirement for instruments, infrastructure, and labor practically facilitated implementation of this methodology for the local production of cellular reagents. Starting with a protein expression plasmid, a bacterial strain suited for protein production, and a DIY bacterial incubator built from locally sourced parts we successfully produced and applied cellular reagents in both Cameroon and Ghana. The breadth of the cellular reagent tool kit and its crossover with DIY efforts implies that evaporated cellular reagents could be useful in implementing educational kits and curricula [43], an especially important application given that a large body of research suggests that hands-on learning experiences are significantly more impactful than observation-based approaches [44, 45]. Cellular reagents should have an ease of use similar to teaching tools that rely on rehydration of freeze-dried cell-free reaction systems [4], but as they require much less instrumentation and expertise to produce compared to cell-free extracts, they could further enhance the flexibility of curriculum design through enabling adaptation and customization by educators.

Besides making local implementation feasible, the minimized process of producing evaporated cellular reagents at source also cuts down the expense and time required for larger scale reagent production. As a result, evaporated cellular reagents can potentially make widespread public health molecular testing programs, such as those employed in surveillance of arthropod-borne diseases and microbial contamination of water bodies, more affordable. In addition, urgent large scale reagent needs, such as those being encountered currently to meet the demand for Covid-19 diagnostics, might be met more readily and in a cost-effective and logistically feasible manner. The quality of these reagents can be ascertained by functional assessment of enzyme activities (e.g., measuring qPCR Cq values, a reliable batch-to-batch property; **S15 Fig in S1 File**). While, other metrics commonly applied to the quality control of pure reagents, such as determining the levels of nucleases and other cellular contaminants, may be more difficult to apply to cellular reagents, it is really the preclinical assessment and qualification of performance that are most important for any reagent. In this regard, cellular reagents with reproducible properties may find application in regulated clinical diagnostics similar to diagnostic methods approved for direct analysis of crude, impure samples, such as SalivaDirect^TM^.

## Supporting information

S1 File (PDF)

S1 Raw images (PDF)

## References

[1] Adebamowo SN, Francis V, Tambo E, Diallo SH, Landoure G, Nembaware V, et al. Implementation of genomics research in Africa: challenges and recommendations. Glob Health Action. 2018; 11(1): 1419033. Epub 2018/01/18. 10.1080/16549716.2017.1419033 29336236 PMC5769805

[2] Abou Tayoun AN, Burchard PR, Malik I, Scherer A, Tsongalis GJ. Democratizing molecular diagnostics for the developing world. Am J Clin Pathol. 2014; 141(1): 17–24. Epub 2013/12/18. 10.1309/AJCPA1L4KPXBJNPG . 24343733

[3] Thavarajah W, Verosloff MS, Jung JK, Alam KK, Miller JD, Jewett MC, et al. A primer on emerging field-deployable synthetic biology tools for global water quality monitoring. npj Clean Water. 2020; 3(1): 18. 10.1038/s41545-020-0064-8 PMC8279131 34267944

[4] Huang A, Nguyen PQ, Stark JC, Takahashi MK, Donghia N, Ferrante T, et al. BioBits™ Explorer: A modular synthetic biology education kit. Science Advances. 2018; 4(8): eaat5105. 10.1126/sciadv.aat5105 30083608 PMC6070312

[5] Castaldo M, Barlind L, Mauritzson F, Wan PT, Snijder HJ. A fast and easy strategy for protein purification using "teabags". Sci Rep. 2016; 6: 28887. Epub 2016/07/01. 10.1038/srep28887 27356497 PMC4928122

[6] Coyle BL, Baneyx F. A cleavable silica-binding affinity tag for rapid and inexpensive protein purification. Biotechnol Bioeng. 2014; 111(10): 2019–2026. Epub 2014/04/30. 10.1002/bit.25257 . 24777569

[7] Hong J, Wang Y, Ye X, Zhang YHP. Simple protein purification through affinity adsorption on regenerated amorphous cellulose followed by intein self-cleavage. J Chromatogr A. 2008; 1194(2): 150–154. 10.1016/j.chroma.2008.04.048 18468611

[8] Bhadra S, Pothukuchy A, Shroff R, Cole AW, Byrom M, Ellefson JW, et al. Cellular reagents for diagnostics and synthetic biology. Plos One. 2018; 13(8): e0201681. Epub 2018/08/16. 10.1371/journal.pone.0201681 30110361 PMC6093680

[9] Harris E. Building scientific capacity in developing countries. EMBO reports. 2004; 5(1): 7–11. Epub 2004/01/08. 10.1038/sj.embor.7400058 14710175 PMC1298969

[10] Maranhao A, Bhadra S, Paik I, Walker D, Ellington AD. An improved and readily available version of Bst DNA Polymerase for LAMP, and applications to COVID-19 diagnostics. medRxiv. 2020: 2020.2010.2002.20203356. 10.1101/2020.10.02.20203356

[11] Liljeruhm J, Funk SK, Tietscher S, Edlund AD, Jamal S, Wistrand-Yuen P, et al. Engineering a palette of eukaryotic chromoproteins for bacterial synthetic biology. Journal of biological engineering. 2018; 12: 8. Epub 2018/05/16. 10.1186/s13036-018-0100-0 29760772 PMC5946454

[12] Gibson DG, Young L, Chuang RY, Venter JC, Hutchison CA, Smith HO. Enzymatic assembly of DNA molecules up to several hundred kilobases. Nat Methods. 2009; 6(5): 343–345. 10.1038/nmeth.1318 ISI:000265661600012. 19363495

[13] Potts M. Desiccation tolerance of prokaryotes. Microbiol Rev. 1994; 58(4): 755–805. Epub 1994/12/01. 7854254 10.1128/mr.58.4.755-805.1994 PMC372989

[14] Jiang YS, Bhadra S, Li B, Wu YR, Milligan JN, Ellington AD. Robust strand exchange reactions for the sequence-specific, real-time detection of nucleic acid amplicons. Anal Chem. 2015; 87(6): 3314–3320. Epub 2015/02/25. 10.1021/ac504387c . 25708458

[15] Notomi T, Okayama H, Masubuchi H, Yonekawa T, Watanabe K, Amino N, et al. Loop-mediated isothermal amplification of DNA. Nucleic Acids Res. 2000; 28(12): E63. Epub 2000/06/28. 10.1093/nar/28.12.e63 10871386 PMC102748

[16] Berninger T, Gonzalez Lopez O, Bejarano A, Preininger C, Sessitsch A. Maintenance and assessment of cell viability in formulation of non-sporulating bacterial inoculants. Microb Biotechnol. 2018; 11(2): 277–301. Epub 2017/12/06. 10.1111/1751-7915.12880 29205959 PMC5812248

[17] Mackey BM, Miles CA, Parsons SE, Seymour DA. Thermal-Denaturation of Whole Cells and Cell Components of Escherichia-Coli Examined by Differential Scanning Calorimetry. J Gen Microbiol. 1991; 137: 2361–2374. ISI:A1991GL15300012. 10.1099/00221287-137-10-2361 1722814

[18] Kahl L, Molloy J, Patron N, Matthewman C, Haseloff J, Grewal D, et al. Opening options for material transfer. Nature biotechnology. 2018; 36(10): 923–927. Epub 2018/10/12. 10.1038/nbt.4263 30307930 PMC6871013

[19] McFarland J. The Nephelometer: an instrument for estimating the number of bacteria in suspensions used for calculating the opsonic index and for vaccines. JAMA. 1907; 49(14): 1176–1178. 10.1001/jama.1907.25320140022001f

[20] Voigt C, Voigt CA. Synthetic Biology, Part A: Methods for Part/Device Characterization and Chassis Engineering: Elsevier Science; 2011.

[21] Baldwin G, Bayer T, Dickinson R, Ellis T, Freemont PS, Kitney RI, et al. Synthetic Biology—A Primer: World Scientific; 2012.

[22] Ellefson JW, Gollihar J, Shroff R, Shivram H, Iyer VR, Ellington AD. Synthetic evolutionary origin of a proofreading reverse transcriptase. Science. 2016; 352(6293): 1590–1593. Epub 2016/06/25. 10.1126/science.aaf5409 . 27339990

[23] Bhadra S, Maranhao AC, Ellington AD. A one-enzyme RT-qPCR assay for SARS-CoV-2, and procedures for reagent production. bioRxiv. 2020: 2020.2003.2029.013342. 10.1101/2020.03.29.013342

[24] Zhang Y, Odiwuor N, Xiong J, Sun L, Nyaruaba RO, Wei H, et al. Rapid Molecular Detection of SARS-CoV-2 (COVID-19) Virus RNA Using Colorimetric LAMP. medRxiv. 2020: 2020.2002.2026.20028373. 10.1101/2020.02.26.20028373

[25] Lamb LE, Bartolone SN, Ward E, Chancellor MB. Rapid detection of novel coronavirus/Severe Acute Respiratory Syndrome Coronavirus 2 (SARS-CoV-2) by reverse transcription-loop-mediated isothermal amplification. Plos One. 2020; 15(6): e0234682. Epub 2020/06/13. 10.1371/journal.pone.0234682 32530929 PMC7292379

[26] Bhadra S, Riedel TE, Lakhotia S, Tran ND, Ellington AD. High-surety isothermal amplification and detection of SARS-CoV-2. mSphere. 2021; 6(3): e00911–e00920. 10.1128/mSphere.00911-20 . 34011690 PMC8265673

[27] Wolfel R, Corman VM, Guggemos W, Seilmaier M, Zange S, Muller MA, et al. Virological assessment of hospitalized patients with COVID-2019. Nature. 2020; 581(7809): 465–469. Epub 2020/04/03. 10.1038/s41586-020-2196-x . 32235945

[28] Casini A, Storch M, Baldwin GS, Ellis T. Bricks and blueprints: methods and standards for DNA assembly. Nature Reviews Molecular Cell Biology. 2015; 16(9): 568–576. 10.1038/nrm4014 WOS:000360342100009. 26081612

[29] Engler C, Kandzia R, Marillonnet S. A one pot, one step, precision cloning method with high throughput capability. Plos One. 2008; 3(11): e3647. Epub 2008/11/06. 10.1371/journal.pone.0003647 18985154 PMC2574415

[30] Green HC, Haugland RA, Varma M, Millen HT, Borchardt MA, Field KG, et al. Improved HF183 Quantitative Real-Time PCR Assay for Characterization of Human Fecal Pollution in Ambient Surface Water Samples. Appl Environ Microbiol. 2014; 80(10): 3086–3094. 10.1128/AEM.04137-13 ISI:000335386200015. 24610857 PMC4018914

[31] Boehm AB, Van De Werfhorst LC, Griffith JF, Holden PA, Jay JA, Shanks OC, et al. Performance of forty-one microbial source tracking methods: A twenty-seven lab evaluation study. Water Res. 2013; 47(18): 6812–6828. 10.1016/j.watres.2012.12.046 ISI:000328444000002. 23880218

[32] Nshimyimana JP, Ekklesia E, Shanahan P, Chua LHC, Thompson JR. Distribution and abundance of human-specific Bacteroides and relation to traditional indicators in an urban tropical catchment. J Appl Microbiol. 2014; 116(5): 1369–1383. 10.1111/jam.12455 ISI:000334062400028. 24460587 PMC4271309

[33] Riedel TE, Zimmer-Faust AG, Thulsiraj V, Madi T, Hanley KT, Ebentier DL, et al. Detection limits and cost comparisons of human- and gull-associated conventional and quantitative PCR assays in artificial and environmental waters. J Environ Manage. 2014; 136: 112–120. Epub 2014/03/04. 10.1016/j.jenvman.2014.01.029 . 24583609

[34] Jiang YS, Riedel TE, Popoola JA, Morrow BR, Cai S, Ellington AD, et al. Portable platform for rapid in-field identification of human fecal pollution in water. Water Res. 2018; 131: 186–195. Epub 2017/12/27. 10.1016/j.watres.2017.12.023 29278789 PMC5999531

[35] Phillips EA, Moehling TJ, Bhadra S, Ellington AD, Linnes JC. Strand Displacement Probes Combined with Isothermal Nucleic Acid Amplification for Instrument-Free Detection from Complex Samples. Anal Chem. 2018; 90(11): 6580–6586. Epub 2018/04/19. 10.1021/acs.analchem.8b00269 29667809 PMC5990927

[36] Briand L, Marcion G, Kriznik A, Heydel JM, Artur Y, Garrido C, et al. A self-inducible heterologous protein expression system in Escherichia coli. Sci Rep. 2016; 6: 33037. Epub 2016/09/10. 10.1038/srep33037 27611846 PMC5017159

[37] Peubez I, Chaudet N, Mignon C, Hild G, Husson S, Courtois V, et al. Antibiotic-free selection in E. coli: new considerations for optimal design and improved production. Microb Cell Fact. 2010; 9: 65. Epub 2010/09/09. 10.1186/1475-2859-9-65 20822537 PMC2941680

[38] Silverman AD, Karim AS, Jewett MC. Cell-free gene expression: an expanded repertoire of applications. Nat Rev Genet. 2020; 21(3): 151–170. Epub 2019/11/30. 10.1038/s41576-019-0186-3 . 31780816

[39] Jung JK, Alam KK, Verosloff MS, Capdevila DA, Desmau M, Clauer PR, et al. Cell-free biosensors for rapid detection of water contaminants. Nature biotechnology. 2020; 38(12): 1451–1459. Epub 2020/07/08. 10.1038/s41587-020-0571-7 32632301 PMC7718425

[40] Pardee K, Green AA, Takahashi MK, Braff D, Lambert G, Lee JW, et al. Rapid, Low-Cost Detection of Zika Virus Using Programmable Biomolecular Components. Cell. 2016; 165(5): 1255–1266. Epub 2016/05/11. 10.1016/j.cell.2016.04.059 . 27160350

[41] Singh-Blom A, Hughes RA, Ellington AD. An amino acid depleted cell-free protein synthesis system for the incorporation of non-canonical amino acid analogs into proteins. J Biotechnol. 2014; 178: 12–22. Epub 2014/03/19. 10.1016/j.jbiotec.2014.02.009 . 24631721

[42] Lee J, Torres R, Kim DS, Byrom M, Ellington AD, Jewett MC. Ribosomal incorporation of cyclic beta-amino acids into peptides using in vitro translation. Chem Commun (Camb). 2020; 56(42): 5597–5600. Epub 2020/05/14. 10.1039/d0cc02121k . 32400780

[43] Heili JM, Gomez-Garcia J, Gaut NJ, Cash BW, Aufdembrink LM, Heffron BA, et al. Real-Time Visualization of in Vitro Transcription of a Fluorescent RNA Aptamer: An Experiment for the Upper-Division Undergraduate or First-Year Graduate Laboratory. J Chem Educ. 2018; 95(10): 1867–1871. 10.1021/acs.jchemed.7b00735 WOS:000447816500025.

[44] Randall WC, Burkholder T. Hands-on laboratory experience in teaching-learning physiology. Am J Physiol. 1990; 259(6 Pt 3): S4–7. Epub 1990/12/01. 10.1152/advances.1990.259.6.S4 . 2256533

[45] Flores Bueso Y, Tangney M. Seeding sustainable education in developing countries: Teaching biotech in low-income areas. EMBO reports. 2020; 21(9): e50587. Epub 2020/09/02. 10.15252/embr.202050587 32869903 PMC7507435

