# Supplementary material for: Producing molecular biology reagents without purification
Source: PLoS One. 2021 Jun 1;16(6):e0252507. doi: 10.1371/journal.pone.0252507 (PMC8168896; doi:10.1371/journal.pone.0252507)
Supplement: S1 File — (PDF) [file pone.0252507.s001.pdf]

Producing molecular biology reagents without purification

Sanchita Bhadra[1,2,*], Vylan Nguyen[3], Jose-Angel Torres[3], Shaunak Kar[1,2], Stéphane Fadanka[6], Chiara Gandini[4], Harry Akligoh[5], Inyup Paik[1,2], Andre C. Maranhao[1,2], Jenny Molloy[4], Andrew D. Ellington[1,2,*]

Supplementary Information

Materials and methods
S1 Table
S2 Table
S1 Fig
S2 Fig
S3 Fig
S4 Fig
S5 Fig
S6 Fig
S7 Fig
S8 Fig
S9 Fig
S10 Fig
S11 Fig
S12 Fig
S13 Fig
S14 Fig
S15 Fig
S16 Fig
References

Material and methods

Plasmids and cloning

Phusion DNA polymerase (NEB) was used for PCR amplification of sequences for subsequent cloning. Standard Gibson assembly techniques were used for cloning unless otherwise noted. Plasmid constructs for expressing Taq DNA polymerase, KlenTaq DNA polymerase, Bst-LF DNA polymerase, RTX DNA polymerase, MMLV RT, and Br512 DNA polymerase were described previously [1, 2]. Briefly, Taq, KlenTaq, Bst-LF, and MMLV-RT are expressed from an anhydrotetracycline inducible pAtetO promoter in the ampicillin resistant pATetO 6xHis plasmid. RTX expression is driven from a T7 promoter-containing pET vector (Addgene; Plasmid #102787). Br512 is also expressed from a T7 promoter in an in-house *E. coli* expression vector, pKAR2 (Addgene; Plasmid #16187).

Construction of T4 DNA ligase, T7 DNA ligase, *Bsa*I, and *Bsm*AIM plasmids

E. coli codon optimized T4 (UniProtKB - P00970) or T7 (UniProtKB - P00969) DNA ligase encoding gene blocks were inserted immediately downstream of the Factor X cleavage site in the pATetO 6xHis plasmid [1, 3]. This is an in-house designed vector based on the pASK-IBA37plus vector (IBA GmbH) from which the multiple cloning site and Rop gene have been removed to improve plasmid copy number [3]. The plasmid also features a modified anhydrotetracycline controlled pATetO promoter with a single point mutation to make it unidirectional. Assembled plasmids were transformed into chemically competent Top10 *E. coli* and verified by Sanger sequencing at the Institute of Cellular and Molecular Biology Core DNA Sequencing Facility.

A constitutive expression plasmid for *Geobacillus stearothermophilus* methyltransferase, *Bsm*AIM (UniProtKB - Q6UQ63), was built by inserting two *E. coli* codon optimized gBlocks comprising the *Bsm*AIM coding sequence downstream of a constitutive pLac promoter in a plasmid bearing pBR322 origin of replication and a kanamycin resistance gene (**S16 Fig**). Assembled plasmid was transformed into chemically competent Top10 *E. coli* and verified by Sanger sequencing at the Institute of Cellular and Molecular Biology Core DNA Sequencing Facility.

An inducible expression system for the *Geobacillus stearothermophilus* restriction endonuclease (*Bsa*IR; UniProtKB - Q6SPF4) was constructed by inserting an *E. coli* codon optimized gBlock comprising the enzyme coding sequence beginning with a TTG start codon in an ampicillin resistant plasmid with a p15a origin of replication and a LacI expression cassette (**S16 Fig**). The *Bsa*IR gene was flanked by an

upstream LacO-regulated T7 promoter and two downstream T7 terminators to control gene expression. Assembled plasmid was co-transformed along with the *Bsm*AIM expression plasmid into chemically competent Top10 *E. coli*. Transformants were allowed to recover for 1 h in a 37 °C shaker prior to adding kanamycin at a concentration of 50 µg/mL and continuing recovery for another 1 h in the 37 °C shaker. Subsequently, the transformants were plated on Luria Bertani agar plates containing 100 µg/mL ampicillin and 50 µg/mL kanamycin and allowed to grow overnight at 30 °C. Plasmids were isolated from individual transformants resistant to both ampicillin and kanamycin and verified by Sanger sequencing.

Construction of pOBL1 expression vector for the expression of OpenVent

pOBL1 carries the gene encoding for an off-patent DNA polymerase from *Pyrococcus sp.* The corresponding protein is sold by NEB under the name DeepVent™ [4, 5]. We have renamed the gene product OpenVent. The protein sequence was retrieved from the patent [4] and back translated. The OpenVent gene sequence was then optimized for expression in *E. coli* using OPTIMIZER [6] (guided random optimization) and domesticated for type II restriction enzymes. The optimized and domesticated sequence was then sent to be chemically synthesized (Twist Bioscience). The gene was cloned in a custom built backbone named pOBL, based on the open source minimal vector built by Staal and co-workers [7] and containing a Kanamycin resistance cassette. pOBL and the OpenVent gene were assembled to give the final pOBL1 using Klenow assembly [8]. pOBL1 map, gb and FASTA files are available in Supporting information (**S11 Fig,** and supplementary Genbank and FASTA files).

McFarland standards preparation

McFarland turbidity standards [9] were prepared as reported in [10, 11]. Prepare a 1.0% (wt/vol) solution of anhydrous barium chloride ($BaCl_2$, 0.048 M) and a 1.0% (vol/vol) solution of sulfuric acid (H_2SO_4, 0.18 M). The solutions are mixed according to the **Table S2.** Turbidity standards should be aliquoted in clear tubes of the same size and shape, the volume of the standard should be filling the tube to a height of around 2.5 cm. Place the tubes in a rack that allows the tubes to be viewed from the side (**S1 Fig**). Print or draw on paper a black and white pattern to place behind the standards. To compare your culture with the standards, you should aliquot some culture in the same tube used for the standards and compare the turbidity against the black and white pattern, diluting your culture if the turbidity is equal to 1 OD_{600}. The sample tube should be placed next to the turbidity standards to be compared side by side. The standard

that best represents the turbidity of the sample will be the one that obscures to the same extent the black and white pattern.

Protein Expression Analysis

For protein analysis, cells were harvested before induction and at the time of final collection. Cell samples equivalent to 1 mL of 1 OD_{600} were harvested in microcentrifuge tubes, centrifuged at 5000 x g for 5 minutes in a table top centrifuge and the supernatant was discarded. Cell pellets were resuspended in 1x Laemli buffer (0.33 M TRIS pH 6.8, 7% SDS, 10% glycerol, 5% 2-Mercaptoethanol), boiled at 95°C for 5 minutes and centrifuged at 13 000 x g for 2 minutes. The supernatant was transferred in a new microcentrifuge tube and the pellet discarded. 10 µL of each sample were loaded on a 12% Bis-Tris gel (SurePAGE, Genscript) and run as suggested by the manufacturer. The protein gel was stained using Quick Coomassie Stain (Generon) (**S4C Fig**).

Assembly instructions for a DIY incubator

Documentation including laser cutting design files and the Arduino firmware program can be found here https://github.com/FOSH-following-demand/Incubator.

The incubator is made up of three main parts:

1. Temperature control unit made of an Arduino Uno that thermostatically controls the temperature using a DHT22 temperature sensor and a 75W incandescent light bulb. The temperature can be visualized by an LCD screen and adjusted through push-buttons. Materials: Arduino UNO, LCD screen, potentiometer, DHT22 temperature sensor, laser cut acrylic box, push buttons, wires as needed.
2. Heat source: an incandescent light bulb.
3. Cooler box: an insulated cooler box to help keep a constant temperature. This should be big enough to contain the cell samples to be grown.

Electronics were wired as shown in the diagram of **S3 Fig** and fitted in a laser cut box. Wires leading to the incandescent bulb and to the temperature sensors were fitted through a hole in the lid of the cooler box and a bulb holder to house the incandescent light bulb was fitted on the other side of the lid. The Arduino program controlled the temperature by turning the bulb on and off in response to the difference between the temperature set by the user and the temperature sensed by the DHT22 temperature sensor. The final build is shown in **S3 Fig, bottom panel**.

Deactivation of living cells in OpenVent cellular reagents

BL21(DE3) *E. coli* strain carrying the pOBL1 expression vector **(S11 Fig)** was grown, induced, harvested, washed and aliquoted as reported above. Cellular reagent samples containing $2x10^8$ cells (enough to perform 10 PCR reactions) were incubated at 50, 60 or 70°C for 10 or 20 minutes. Untreated cells were processed as per standard protocol. All the samples were then dried overnight (>16 h) at 37°C. The following day, each sample was resuspended in the appropriate amount of water (see method above). 1/10 of each sample of cellular reagents was used to perform a PCR reaction with a 700 bp amplicon to assay the enzyme activity after treatment **(S4B Fig)**. The remaining cellular reagents were plated on LB agar without antibiotics. The plates were incubated overnight at 37°C and cell growth visualized the next day (**S4A Fig**).

PCR using evaporated cellular reagents

All assay mixes were assembled and kept on ice prior to initiation of amplification. Endpoint PCR reactions were assembled in 25 µL (CT16S) or 50 µL (TtgR and HF183) volumes containing 10 ng or indicated amounts of plasmid templates along with a final concentration of 500 nM each of CT16.FWD and CT16.REV primers, 400 nM each of TtgR.FWD and TtgR.REV primers, or 200 nM each of HF183.FWD and fluorescein-labeled HF183.REV primers along with 100 nM of biotinylated HF183 probe. Amplification was performed in 1X ThermoPol buffer (NEB) (20 mM Tris-HCl, 10 mM $(NH_4)_2SO_4$, 10 mM KCl, 2 mM $MgSO_4$, 0.1% Triton® X-100, pH 8.8@25°C) containing 0.2 mM deoxyribonucleotide mix (dNTPs) and an indicated enzyme source – 3 µL (2×10^7 cells) of Taq DNA polymerase cellular reagents freshly rehydrated in 30 µL water, one 3 mm diameter paper disc containing 2×10^7 dried Taq polymerase cellular reagents directly added to and kept immersed in the PCR reaction, or 2.5 units of pure Taq DNA polymerase. Following an initial 2 min incubation at 94-95 °C, thermocycling was performed (CT16S: 20 cycles of 30 sec at 95 °C, 15 sec at 55 °C, and 1 min at 72 °C; TtgR: 30 cycles of 30 sec at 94 °C, 15 sec at 55 °C, and 1 min at 72 °C and a final incubation of 3 min at 68 °C; HF183: 20 cycles of 30 sec at 95 °C, 10 sec at 60 °C, and 30 sec at 72 °C followed by a final denaturation at 95 °C for 30 sec and rapid cooling and 1 min incubation at 4 °C to enable probe hybridization). Seven to ten microliters of the resultant PCR products were analyzed by agarose gel electrophoresis. Colorimetric readout of HF183 PCR was performed by diluting the PCR reaction with 25 µL of lateral flow buffer (Milenia Biotec GmbH, Giessen, Germany) and analyzing on a fluorescein/biotin-specific lateral flow dipstick (Milenia HybriDetect 1, Milenia Biotec GmbH) according to the manufacturer's instructions.

Quantitative PCR reactions with Taq or KlenTaq polymerases were prepared in 25 µL volume containing indicated amounts of pCR2.1-CT16S plasmid template along with a final concentration of 0.5 µM or 0.4 µM each of forward (CT16.FWD) and reverse (CT16.REV) primers, respectively. Amplification was performed in 1X ThermoPol (Taq) or 1X KlenTaq1 buffer (DNA Polymerase Technology) (50mM Tris-Cl pH 9.2, 16 mM ammonium sulfate, 0.05% Brij 58, and 3.5 mM magnesium chloride) containing 0.4 mM dNTPs, 1X EvaGreen intercalating dye (Biotium, Freemont, CA), and an enzyme source – 0.2 µL of pure KlenTaq1, 3 µL of KlenTaq or Taq cellular reagents freshly rehydrated in 30 µL water, or 3 mm paper discs containing 2 x 10^7 evaporated KlenTaq cellular reagents. Assays were subjected to 10 min at 95 °C followed by 45 cycles of 10 sec at 95 °C (denaturation), 15 sec at 55 °C (annealing) and 30 sec at 72 °C (extension) using a LightCycler 96 real-time PCR machine. Fluorescence signals were recorded in the FAM channel during the extension step in each cycle. Following qPCR amplification, amplicon melt curve analysis was performed. All data were analyzed using the LightCycler 96 software.

LAMP-OSD

All assay mixes were assembled and kept on ice prior to initiation of amplification. Human glyceraldehyde-3-phosphate dehydrogenase (*gapd*) and HF183 LAMP-OSD reaction mixtures were prepared in 25 µL of 1X isothermal buffer (NEB) (20 mM Tris-HCl, 10 mM $(NH4)_2SO_4$, 50 mM KCl, 2 mM $MgSO_4$, 0.1% Tween-20, pH 8.8) containing indicated DNA templates along with 1.6 µM each of BIP and FIP primers, 0.4 µM each of B3 and F3 primers, 0.8 µM of unmodified (*gapd*) or biotinylated (HF183) loop primer, 0.8 M (*gapd*) or 1 M (HF183) betaine, 0.8 mM dNTPs, 2 mM additional $MgSO_4$, and 100 nM of fluorescein-labeled OSD strand pre-annealed with 5-fold (*gapd*) or 2-fold (HF183) excess of the quencher labeled OSD strand. Multiplex 25 µL RT-LAMP-OSD assays containing indicated copies of inactivated SARS-CoV-2 virions were assembled in 1X G6D buffer (60 mM Tris-HCl, pH 8.0, 2 mm $(NH_4)_2SO_4$, 40 mM KCl, 8 mM $MgCl_2$) containing 1.4 mM dNTPs, 0.4 M betaine, 6-Lamb primers (1.2 µM each of FIP and BIP, 0.6 µM each of LF and biotinylated LB, 0.3 µM each of F3 and B3), NB primers (1.2 µM each of FIP and BIP, 0.2 µM additional biotinylated FIP and BIP, 0.6 µM LB, 0.3 µM each F3 and B3), and 100 nM fluorescein-labeled 6-Lamb and NB OSD strands pre-annealed with 3-fold and 5-fold excess of quencher strands, respectively. All OSDs were annealed in 1X isothermal buffer by incubation at 95 °C for 1 min followed by cooling at the rate of 0.1 °C/sec to 25 °C. Amplification was performed using indicated enzymes – 16 units of Bst 2.0 (NEB), 3 µL of Bst-LF-expressing BL21 (*gapd*) or Acella (HF183) cellular reagents hydrated in

30 µL water, a 3 mm paper disc containing 2 x 10^7 evaporated Bst-LF cellular reagents, or 3 µL of Br512-expressing BL21(DE3) cellular reagents rehydrated in 30 µL water and pretreated at 65 °C for 30 min prior to use in amplification. *Gapd* assays were incubated at 65 °C in a LightCycler 96 real-time PCR machine (Roche, Basel, Switzerland) and OSD fluorescence signals were recorded every 3 min and analyzed using the LightCycler 96 software. HF183 assays were incubated at 60 °C for 90 min while SARS-CoV-2 assays were kept at 65 °C for 1 h followed by 1 min incubation at 95 °C. Endpoint OSD fluorescence was observed at room temperature using blue LED transilluminator and imaged using a ChemiDoc camera (Bio-Rad, Hercules, CA, USA). Colorimetric readout of HF183 assays was performed using U-Star fluorescein/biotin closed-tube lateral flow devices (Ustar Biotechnologies, Zhejiang, China distributed by TwistDx, Maidenhead, UK) while SARS-CoV-2 assays were diluted with 25 µL of lateral flow buffer (Milenia Biotec) and analyzed using Milenia HybriDetect 1 fluorescein/biotin lateral flow dipsticks (Milenia Biotec GmbH).

Two-step quantitative reverse transcription (RT) PCR using MMLV-RT

All assay mixes were assembled and kept on ice prior to incubation at indicated temperatures. Indicated amounts of *in vitro* transcribed and polyacrylamide gel purified Zika virus NS5 RNA templates were mixed with 10 µM reverse (Zika-4552c) primers and 1 mM dNTP in a total volume of 10 µL. Primer template annealing was performed by incubating the solutions at 65 °C for 5 min followed by 2 min on ice. Reverse transcription was initiated by adding a 10 µL solution containing 2X MMLV RT buffer (NEB) (100 mM Tris-HCl, 20 mM DTT, 150 mM KCl, 6 mM $MgCl_2$, pH 8.3), 8 units of RNase inhibitor and 3 µL (2 x 10^7 cells) of MMLV reverse transcriptase expressing evaporated cellular reagents rehydrated prior to use in water. In some reactions, reverse transcription was performed by directly adding 3 mm paper discs containing 2 x 10^7 evaporated MMLV RT cellular reagents. Following 1 h of reverse transcription at 42 °C, 5 µL of the resulting cDNA-containing solution was analyzed by TaqMan qPCR using Taq DNA polymerase. Briefly, Taq DNA polymerase TaqMan qPCR reactions were prepared in 25 µL volume containing a final concentration of 0.32 µM each of forward (Zika-4481_F) and reverse (Zika-4552c) primers [12]. Amplification was performed in 1X ThermoPol buffer (NEB) containing 0.4 mM dNTPs, 2.5 units of Taq DNA polymerase, and 80 nM TaqMan probe (Zika-4507c-FAM) [12]. For real-time signal measurement, the TaqMan qPCR reactions were placed in a LightCycler 96 real-time PCR machine and subjected to 10 min at 95 °C followed by 45 cycles of 15 sec at 95 °C (denaturation) and 30 sec at 55 °C (annealing and

extension). Fluorescence signals were recorded in the FAM channel during the annealing/extension step in each cycle. All data were analyzed using the LightCycler 96 software.

Assembly instructions for a DIY fluorescence visualization device

Documentation including parts list, circuit designs, and assembly instructions can be found at https://github.com/Jose-4625/LAMP-BOX-ECO/tree/master/VisBox.

SARS-CoV-2 RT-qPCR using RTX polymerase

All assay mixes were assembled and kept on ice prior to initiation of amplification. RT-qPCR reactions were set up in 50 µL volume in 1X RTX buffer (60 mM Tris-HCl, 25 mM $(NH_4)_2SO_4$, 10 mM KCl, 2 mM $MgSO_4$, pH 8.4 @25 °C) supplemented with 1X EvaGreen intercalating dye (Biotium), 500 nM each of SARS-CoV-2 N1-fwd and N1-Rev primers, 0.4 mM dNTP, 0.5 M betaine, and some 2 x 10^6 BL21(DE3) RTX cellular reagents hydrated in water immediately before use. Different amounts of armored SARS-CoV-2 N RNA (Asuragen) were added to the reactions, which were then immediately transferred to a LightCycler 96 real time PCR machine and incubated at 95 °C for 5 min followed by 30 min at 60 °C to allow reverse transcription. The reactions were then subjected to 10 min incubation at 95 °C followed by 55 cycles of 15 sec incubation at 95 °C and 30 sec incubation at 55 °C. Fluorescence was measured in the FAM channel during the second step of each cycle. Following completion of amplification, amplicons were subjected to standard melt curve analysis on the LightCycler 96 by incubating them at 95 °C for 10 sec followed by 60 sec at 65 °C and then slowly ramping up the temperature to 97 °C at the rate of 0.05 °C/sec while making 20 measurements of fluorescence in the FAM channel per 1 °C change in temperature. All analysis was performed using LightCycler 96 software.

DNA restriction digestion using cellular reagents

Restriction digestion reactions were set up in 20 µL volume containing 1X CutSmart buffer (NEB; 50 mM Potassium Acetate, 20 mM Tris-acetate, 10 mM Magnesium Acetate, 100 µg/mL BSA, pH 7.9@25°C), 225 ng of plasmid with two *Bsa*I restriction sites flanking a 745 bp insert, and 2 x 10^7 *Bsa*IR or Taq cellular reagents or 20 units of pure *Bsa*I-HF-V2 (NEB) enzyme. The reactions were incubated at 37 °C for 2 h followed by 20 min at 65 °C. Some 10 µL of the reaction products were analyzed by agarose gel electrophoresis.

S1 Table. Oligonucleotide and template sequences for nucleic acid amplification reactions used in the study.		
**Name**	**Sequence**	**Use**
CT16.FWD	TAGTGGCGGAAGGGTTAG	*Chlamydia trachomatis* (CT16S) PCR
CT16.REV	CGTCATAGCCTTGGTAGG	
CT16S *Chlamydia trachomatis* sequence in template plasmid	CGCCAAGCTTGGTACCGAGCTCGGATCCACTAGTAACGGCCGCCAGTGTGCTG<br>GAATTCTAATACGACTCACTATAGGGCAATTGTTTAGTGGCGGAAGGGTTAGTA<br>ATGCATAGATAATTTGTCCTTAACTTGGGAATAACGGTTGGAAACGGCCGCTAAT<br>ACCGAATGTGGCGATATTTGGGCATCCGAGTAACGTTAAAGAAGGGGATCTTAG<br>GACCTTTCGGTTAAGGGAGAGTCTATGTGATATCAGCTAGTTGGTGGGGTAAAG<br>GCCTACCAAGGCTATGACGTCTAGGCGGATTGAGAGATTGGCCGCCAACACTG<br>GGACTGAGACACTGCCCAGACTCCTACGGGAGGCTGCAGTCGAGAATCTTTCG<br>CAATGGACGGAAGTCTGACGAAGCGACGCCGCGTGTGTGATGAAGGCTCTAGG<br>GTTGTAAAGGAATTCTGCAGATATCCATCACACTGGCGGCCGCTCGAGCATGCA<br>TCTAGAGGGCCCAATT	
gapdLAMP.F3	GCCACCCAGAAGACTGTG	gapd LAMP-OSD
gapdLAMP.B3	TGGCAGGTTTTTCTAGACGG	
gapdLAMP.FIP	CGCCAGTAGAGGCAGGGATGAGGGAAACTGTGGCGTGAT	
gapdLAMP.BIP	GGTCATCCCTGAGCTGAACGGTCAGGTCCACCACTGACAC	
gapdLAMP.LR	TGTTCTGGAGAGCCCCGCGGCC	
gapdOSD.F	/56-FAM/CTCACTGGCATGGCCTTCCGTGTCCCCACTGCCAAC/3InvdT/	
gapdOSD.Q	GGACACGGAAGGCCATGCCAGTGAG/3IABkFQ/	
gapd sequence in template plasmid	CCACAGTCCATGCCATCACTGCCACCCAGAAGACTGTGGATGGCCCCTCCGGG<br>AAACTGTGGCGTGATGGCCGCGGGGCTCTCCAGAACATCATCCCTGCCTCTAC<br>TGGCGCTGCCAAGGCTGTGGGCAAGGTCATCCCTGAGCTGAACGGGAAGCTCA<br>CTGGCATGGCCTTCCGTGTCCCCACTGCCAACGTGTCAGTGGTGGACCTGACC<br>TGCCGTCTAGAAAAACCTGCCAAATATGATGACATCAAGAAGGTGGTGAAGCAG<br>GCGTCGGAGGGCCCCCTCAAGGGCATCCTGGGCTACACTGAGCACCAGGTGG<br>TCTCCTCTGACTTCAACAGCGACACCCACTCCTCCACCTTTGACGCTGGGGCTG<br>GCATTGCCCTCAACGACCACTTTGTCAAGCTCATTTCCTG	
Zika 4481_F	CTGTGGCATGAACCCAATAG	Zika TaqMan q(RT)PCR
Zika 4552c	ATCCCATAGAGCACCACTCC	
Zika 4507c-FAM	/56-FAM/CCACGCTCCAGCTGCAAAGG/3IABkFQ/	
Zika TaqMan template	GGGAC CATCTGTGGCATGAACCCAA TAGCCATACC CTTTGCAGCT<br>GGAGCGTGGT ACGTGTATGT GAAGACTGGAAAAAGGAGTG GTGCTCTATG<br>GGATGTGCCT	
TtgR.FWD	CCACGTATCAGAAGGAGGTTAGTATATGGTTCGTCGTACCAAAGAAGAGGC	TtgR PCR
TtgR.REV	CTGCACTCCTCGAATTCTTAGGATTATTATTTACGCAGTGCCGGACTCAG	
TtgR sequence in template plasmid	ATGGTTCGTCGTACCAAAGAAGAGGCACAAGAAACCCGTGCACAGATTATTGAA<br>GCAGCAGAACGTGCATTCTATAAACGTGGTGTTGCACGTACCACCCTGGCAGAT<br>ATTGCAGAACTGGCAGGCGTTACCCGTGGTGCAATTTATTGGCATTTTAACAAC<br>AAAGCCGAACTGGTTCAGGCACTGCTGGATAGCCTGCATGAAACCCATGATCAT<br>CTGGCACGTGCAAGCGAAAGCGAAGATGAAGTTGATCCGCTGGGTTGTATGCG<br>TAAACTGCTGCTGCAGGTTTTTAATGAACTGGTTCTGGATGCACGTACCCGTCG<br>TATTAATGAAATCCTGCATCACAAATGCGAGTTCACCGATGATATGTGTGAAATT<br>CGTCAGCAGCATCAGAGCGCAGTTCTGGATTGTCATAAAGGTATTACCCTGACA<br>CTGGCAAATGTAGTTCGTCGTGGTCAGCTGCCTGGTGAACTGGATGCAGAACG<br>TGCCGCAGTTGCAATGTTTGCCTATGTTGATGGTCTGATTCGTCGTTGGCTGCT<br>GCTGCCGGATAGCGTTGATCTGCTGGGTGATGTTGAAAAATGGGTTGATACCG<br>GTCTGGATATGCTGCGTCTGAGTCCGGCACTGCGTAAATAATAATCCTGAG	
NB-F3	ACCGAAGAGCTACCAGACG	SARS-CoV-2 multiplex RT-LAMP-OSD assay for fluorogenic and lateral flow readout
NB-B3	TGCAGCATTGTTAGCAGGAT	
NB-FIP	TCTGGCCCAGTTCCTAGGTAGTTCGTGGTGGTGACGGTAA	
NB-BIP	AGACGGCATCATATGGGTTGCACGGGTGCCAATGTGATCT	
NB-BIP-Bio	/5Biosg/AGACGGCATCATATGGGTTGCACGGGTGCCAATGTGATCT	
NB-FIP-Bio	/5Biosg/TCTGGCCCAGTTCCTAGGTAGTTCGTGGTGGTGACGGTAA	
NB-LB	ACTGAGGGAGCCTTGAATACA	
NB-OSD-FAM	/56-FAM/CCGAATGAAAGATCTCAGTCCAAGATGGTATTTCT/3InvdT/	
NB-OSD-Q	TCTTGGACTGAGATCTTTCATTCGG/3IABkFQ/	
Lamb-F3	TCCAGATGAGGATGAAGAAGA	
Lamb-B3	AGTCTGAACAACTGGTGTAAG	
Lamb-FIP	AGAGCAGCAGAAGTGGCACAGGTGATTGTGAAGAAGAAGAG	
Lamb-BIP	TCAACCTGAAGAAGAGCAAGAACTGATTGTCCTCACTGCC	
Lamb-LF	CTCATATTGAGTTGATGGCTCA	
Lamb-LB	ACAAACTGTTGGTCAACAAGAC	
Lamb-LB-Bio	/5Biosg/ACAAACTGTTGGTCAACAAGAC	
Lamb-OSD-FAM	GTATGGTACTGAAGATGATTACCAAGGTAAACCTTTGGAATTTGGAC/36-FAM/	
Lamb-OSD-Q	/5IABkFQ/GTCCAAATTCCAAAGGTTTACCTTGGTAATCATCTC/3InvdT/	

HF183.FWD	ATCATGAGTTCACATGTCCG	HF183 PCR and lateral flow readout
HF183.FAM.REV	/56-FAM/CGTAGGAGTTTGGACCGTGT	
HF183.probeBio	ATG CGT TCC ATT AGA TAG TA/3Bio/	
HF183.LAMP.FIP	GAACGCATCCCCATCGTCTACCATCATGAGTTCACATGTCCG	HF183 LAMP-OSD with fluorogenic and lateral flow readout
HF183.LAMP.BIP	GGTAACGGCCCACCTAGTCAACCCGTGTCTCAGTTCCAATGT	
HF183.LAMP.F3	GCCAGCCTTCTGAAAGGAAG	
HF183.LAMP.B3	TGCCTCCCGTAGGAGTTTG	
HF183.LAMP.LP	/5Biosg/GGAAAATACCTTTAATCATG	
HF183.OSD.F	GATGGATAGGGGTTCTGAGAGGAAGGTCCC AGC/36-FAM/	
HF183.OSD.Q	/5IABkFQ/GCTGGGACCTTCCTCTCAGAAC/3InvdT/	
2019-nCoV_N1-F	5’-GAC CCC AAA ATC AGC GAA AT-3’	SARS-CoV-2 N1 RT-qPCR
2019-nCoV_N1-R	5’-TCT GGT TAC TGC CAG TTG AAT CTG-3’	

S2 Table. Preparation of McFarland turbidity standards and corresponding OD_{600} value. The indicated μL quantities are for the preparation of 3mL of standards, the volumes can be scaled up or down accordingly.

1% $BaCl_2$ (μL)	1% H_2SO_4 (μL)	%vol/vol of 1% $BaCl_2$ on total volume	OD_{600}
0	3,000	0	0
10.89	2,989	0.363	0.1
21.78	2,978	0.726	0.2
32.67	2,967	1.089	0.4
43.56	2,956	1.452	0.4
54.45	2,945	1.815	0.5
65.34	2,934	2.111	0.6
76.23	2,923	2.541	0.7
87.12	2,912	2.904	0.8
98.01	2,901	3.267	0.9
108.9	2,890	3.63	1

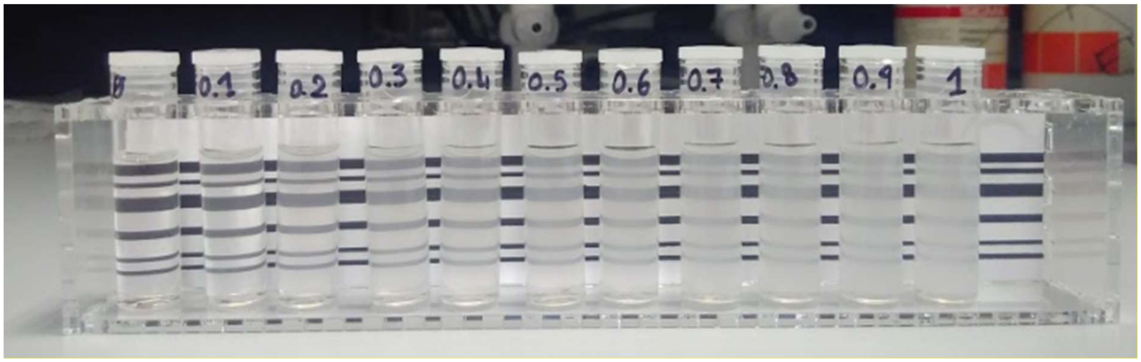

S1 Fig. McFarland turbidity standards. The standards are visualized from the side on a test tube rack against a drawn black and white pattern. The sample to be tested must be aliquoted in the same type of tube used for the standards.

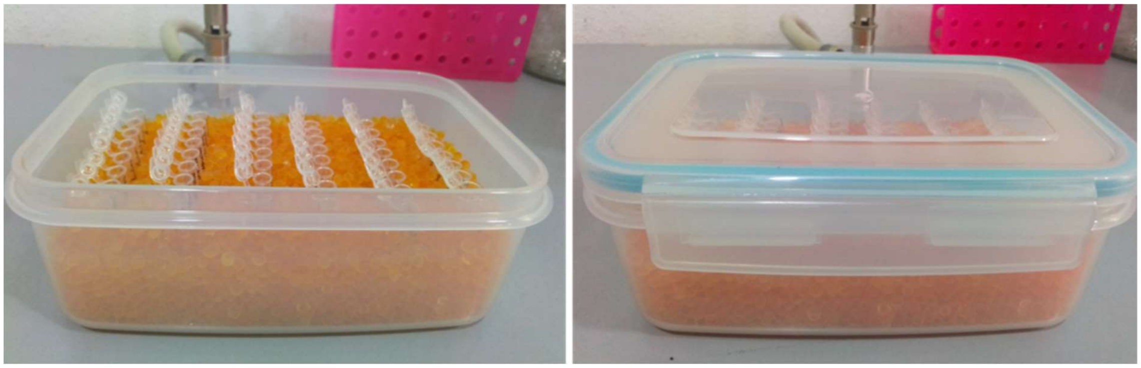

S2 Fig. Sample disposition in the air-tight container for the preparation of evaporated OpenVent cellular reagents.

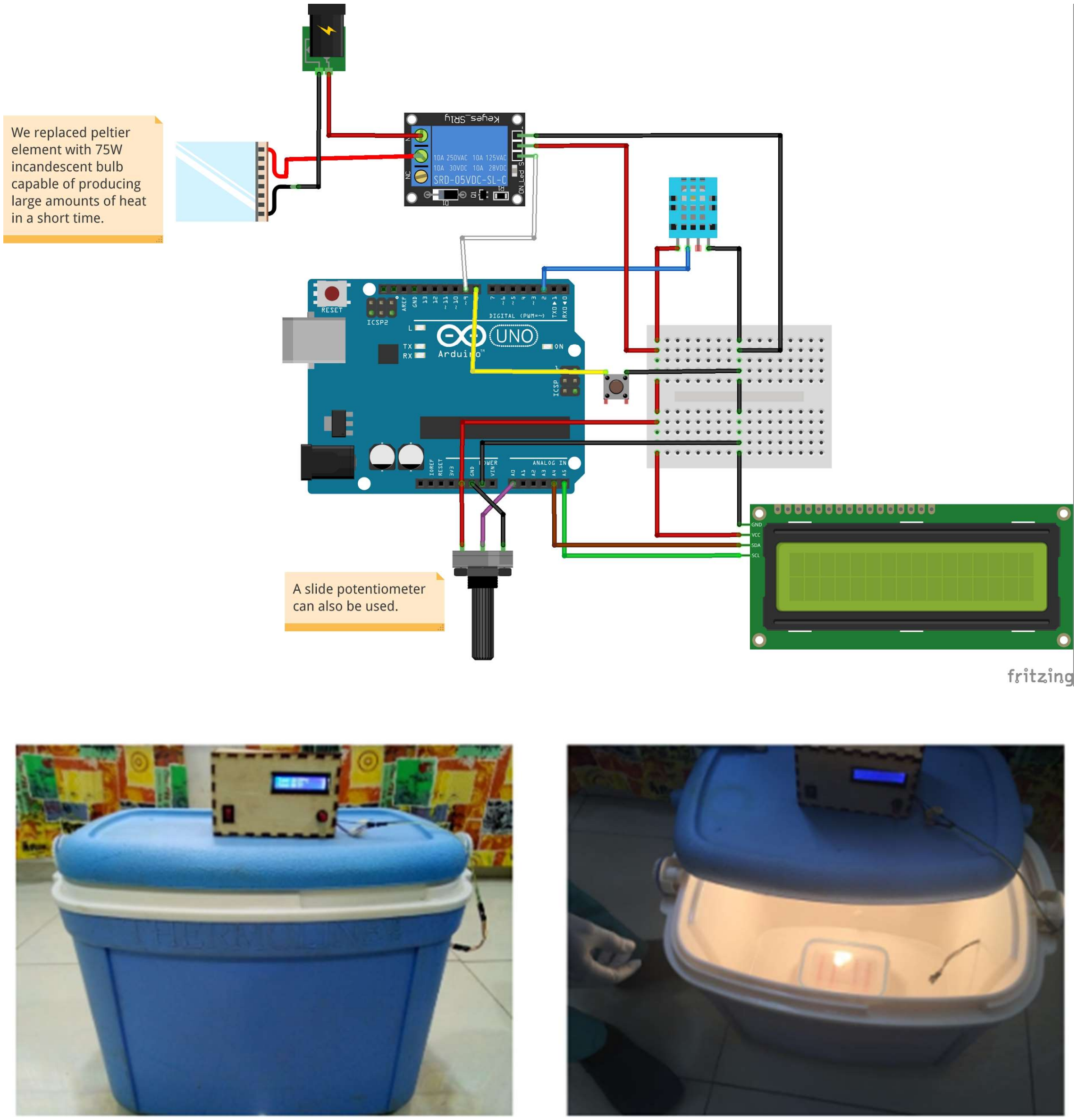

S3 Fig. DIY incubator built from locally sourced materials. Top panel: Arduino scheme to control the DIY bioreactor. Bottom panel: DIY incubator final assembly.

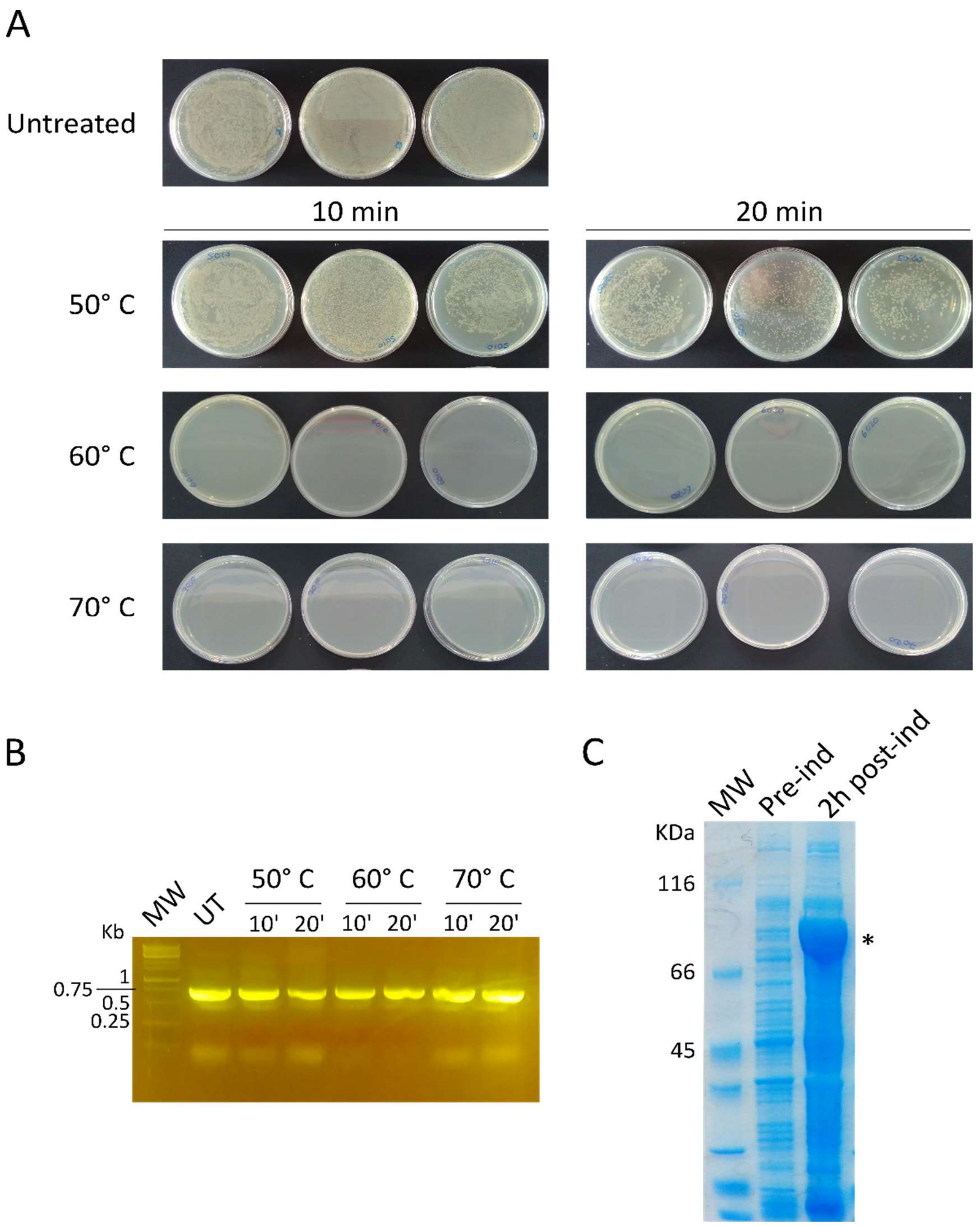

S4 Fig. Heat treatment of cellular reagents for 10 minutes at 60°C is enough to deactivate living cells while maintaining DNA polymerase activity. A) Evaporated cellular reagents contain living cells (untreated sample). Different heat treatments were tested to deactivate living cells. Prior to desiccation, three tubes of OpenVent polymerase cellular reagents (containing ~ $2x10^8$ cells, enough to perform 10 PCR reactions) were heat treated at 50, 60, or 70°C for either 10 or 20 minutes. After evaporation, the cellular reagents were rehydrated and plated on LB plates without antibiotics. **B)** Before plating, 2 µL of each sample was used to assay the OpenVent activity against a 700bp amplicon. None of the heat treatments deactivated OpenVent. **C)** SDS-PAGE analysis of OpenVent expression in the culture used for the experiment. MW: Molecular Weight (Thermo Fisher Pierce Unstained protein MW marker, cat. 26610); Pre-ind: pre-induction; Post-ind: 2 hours post induction (time of harvest).

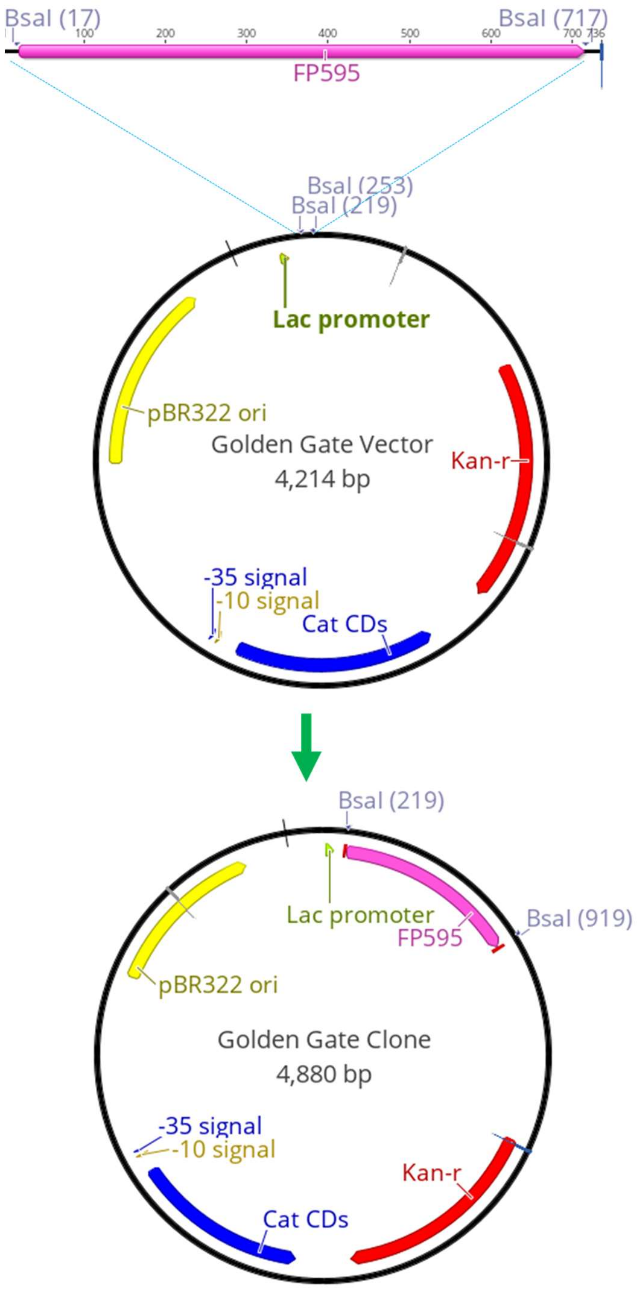

S5 Fig. Vector and insert design for demonstrating Golden Gate cloning using evaporated cellular reagents. A PCR product comprised of the FP595 chromoprotein coding sequence flanked by two *Bsa*I recognition sites served as the insert. A plasmid containing a constitutive Lac promoter upstream of two *Bsa*I sites served as the vector. Following Golden Gate assembly, the FP595 coding sequence is positioned downstream of the Lac promoter leading to expression of pink colored chromoprotein.

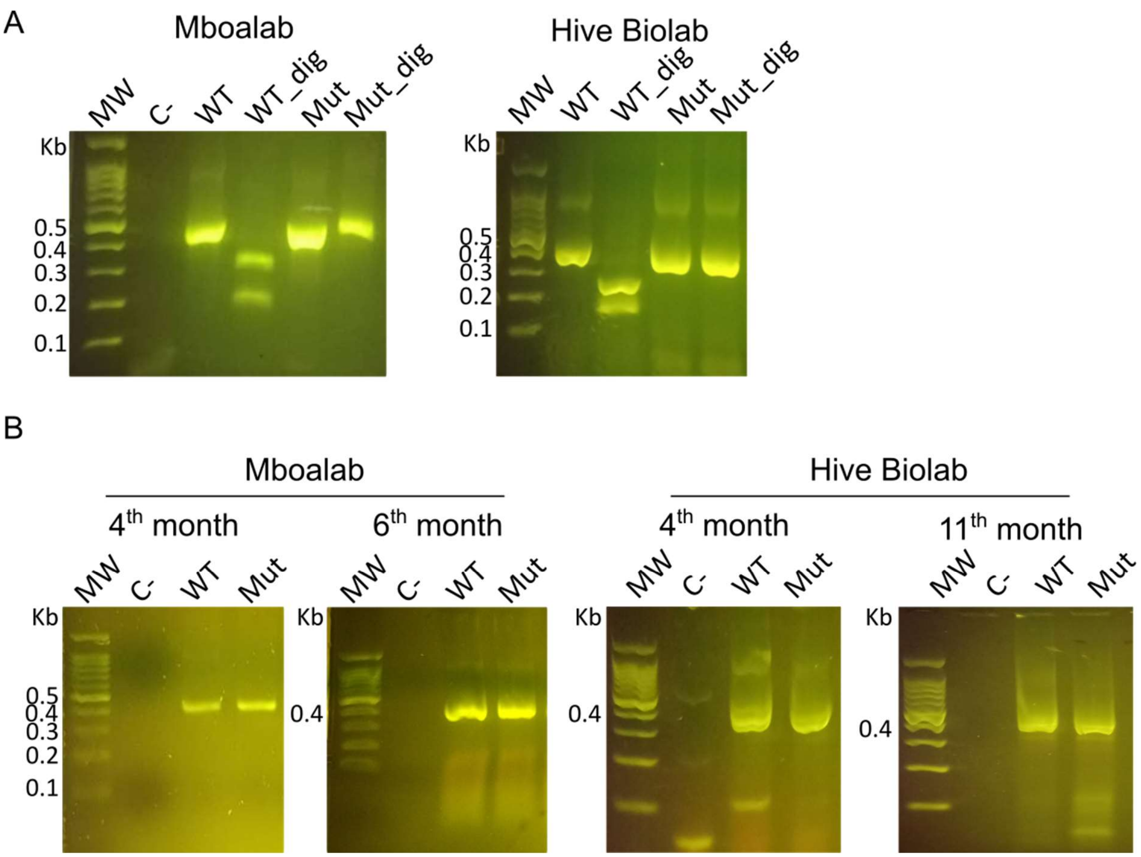

S6 Fig. Use and stability of freeze-dried Taq DNA polymerase cellular reagents in low-resources settings. A) Freeze-dried Taq DNA polymerase cellular reagents were used successfully at Mboalab, (Yaounde, Cameroon) and at Hive Biolab (Kumasi, Ghana) during an educational workshop on sickle cell gene detection in July 2019 [13]. Cellular reagents were used to amplify a synthetic human β-globin wild-type (WT) and mutant (Mut) gene variants. After amplification, both variants were digested with DdeI restriction enzyme. DdeI can digest the WT allele of the beta-globin gene (WT_dig) while it cannot digest its mutant variant (Mut_dig). **B)** Stability of the freeze-dried preparation was assayed by performing a PCR on the WT and Mut variants of synthetic human β-globin at the time and place indicated above the panels. The shorter duration of Mboalab testing is due to the original batch of freeze dried cellular reagents being used up in additional experiments. MW: molecular weight (100bp DNA ladder, Newmarket Scientific); C-: negative control.

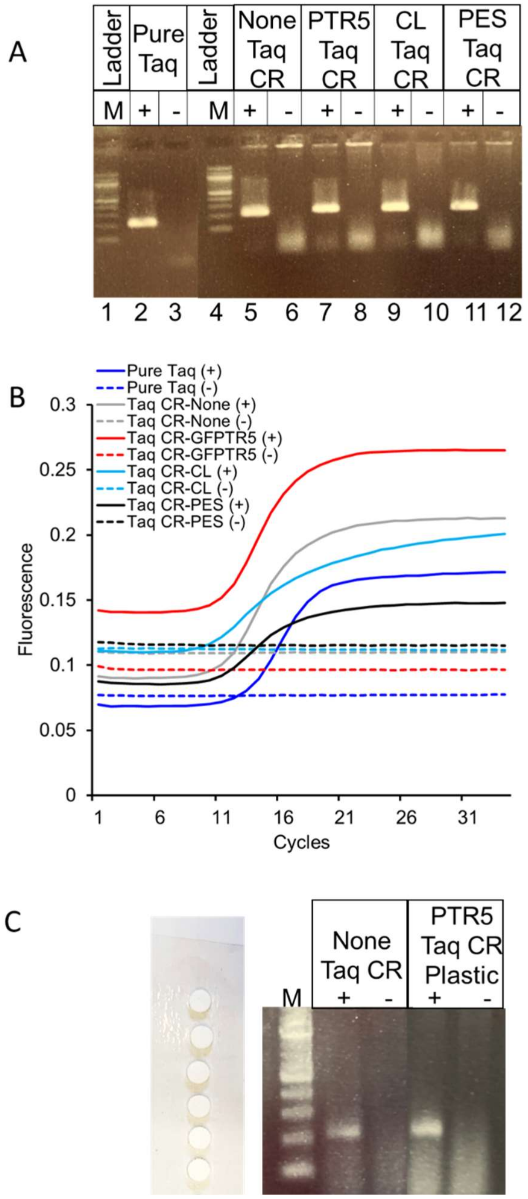

S7 Fig. Comparison of Taq cellular reagents lyophilized without solid support ('None') or lyophilized on glass fiber ('GF-PTR5'), cellulose ('CL'), or polyethersulfone ('PES') paper discs. (**A**) Activities of cellular reagents were compared by performing 20 cycle PCR amplification of CT16S templates (lanes labeled with '+') by adding either 3 µL of rehydrated cellular reagents prepared without solid support or a single 3 mm paper disc containing lyophilized cellular reagents into the PCR reaction mix. Duplicate reactions lacking templates (lanes labeled with '-') were used as negative controls. PCR reactions performed using commercially obtained pure Taq enzyme served as positive controls. (**B**) Activities were compared by performing TaqMan qPCR using a synthetic DNA template derived from Zika virus genomic sequence. PCR reactions were provided with pure commercial Taq (dark blue traces), 3 µL of rehydrated cellular reagents prepared without solid support (gray traces), or lyophilized cellular reagent filled 3 mm paper discs made of cellulose (light blue traces), polyethersulfone (black traces), or glass fiber conjugate paper (red traces). Representative amplification curves are depicted. (**C**) Taq DNA polymerase cellular reagents prepared by evaporation on PTR5 glass fiber paper discs stuck on laminating plastic (left panel) tested by directly adding a punched out piece of the plastic bearing one cellular reagent disc into a PCR reaction. Agarose gel electrophoretic analysis of PCR amplification performed in the presence (+) or absence (-) of templates is depicted. Reactions performed using cellular reagents evaporated without solid support served as control. Data shown are representative of three biological replicates.

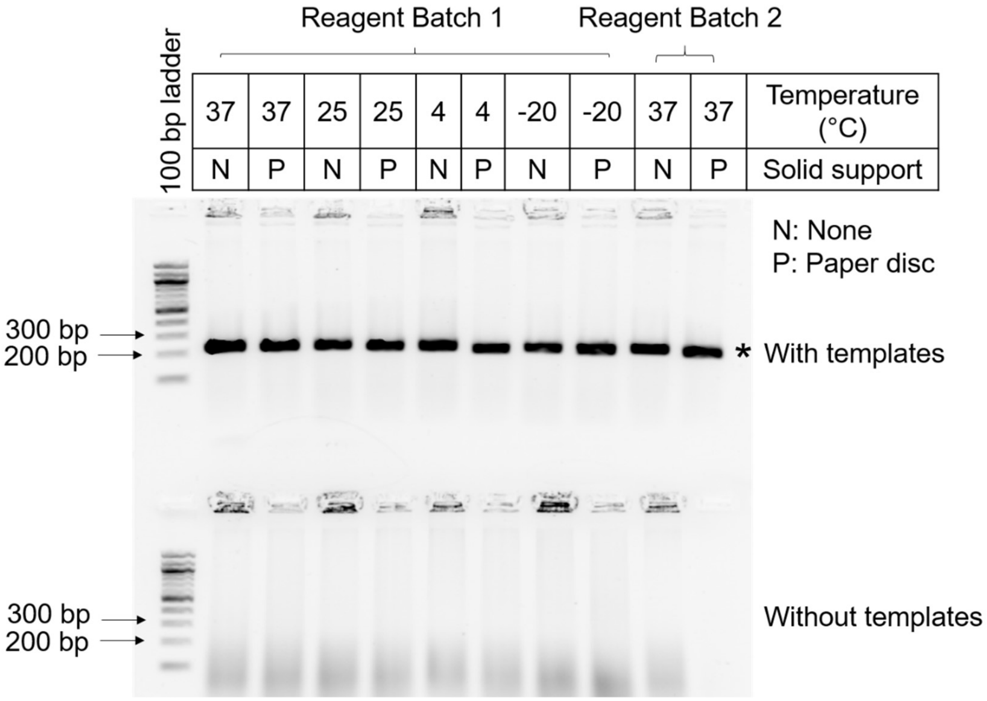

S8 Fig. Storage stability of evaporated Taq DNA polymerase cellular reagents. Two batches of Taq DNA polymerase cellular reagents evaporated at the indicated temperature with (P) or without (N) solid support were tested after 2-2.5 months of preparation by performing 20 cycle PCR reactions with or without CT16S plasmid templates. Agarose gel electrophoretic analysis of resulting PCR amplicons is depicted. Expected products are indicated by an asterisk.

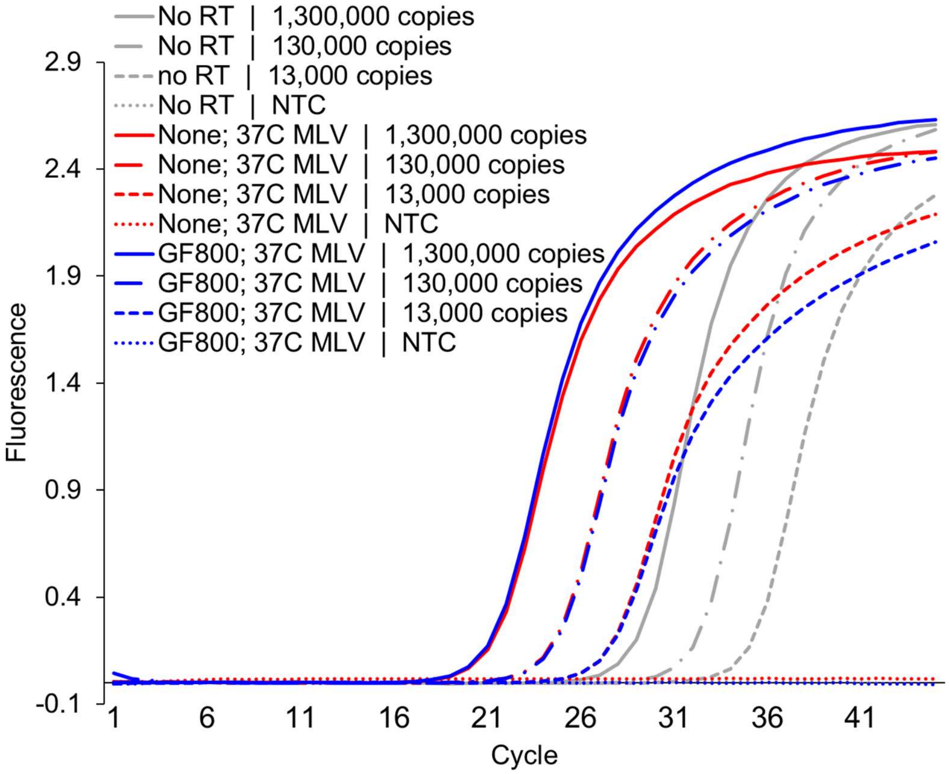

RNA copies	No RT	None; 37 C MLV	GF800; 37C MLV
1,300,000	27.17 ± 0.30	19.33 ± 0.41	19.18 ± 0.32
130,000	30.43 ± 0.30	22.62 ± 0.40	22.60 ± 0.45
13,000	33.22 ± 0.50	25.52 ± 0.58	25.68 ± 0.50

S9 Fig. TaqMan RT-qPCR assay using MLV-RT evaporated cellular reagents. MLV reverse transcriptase cellular reagents evaporated at 37 °C with (GF800) or without (None) solid support were tested in reverse transcription TaqMan qPCR assays using indicated copies of synthetic Zika virus RNA templates and pure Taq DNA polymerase. Assays performed without any added reverse transcriptase served as control. Representative amplification curves (top panel) and average Cq values and standard deviations (lower panel) observed by measuring increase in TaqMan probe fluorescence in real-time in triplicate experiments are depicted.

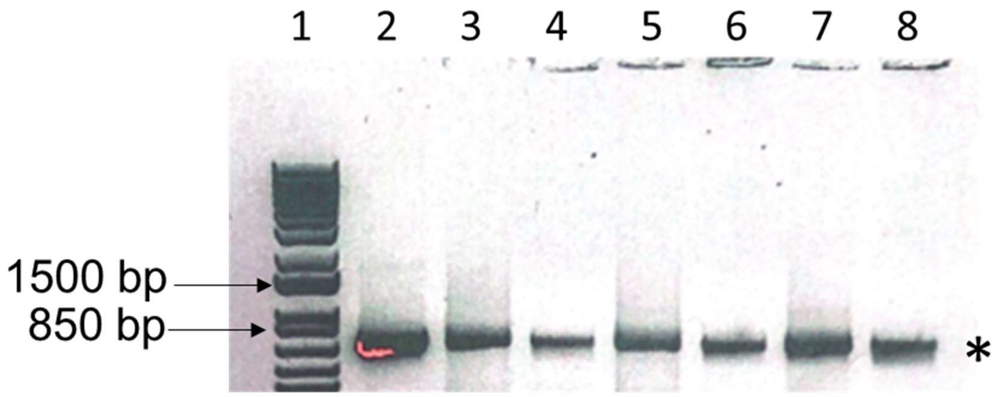

S10 Fig. Endpoint PCR using Taq DNA polymerase cellular reagents dried in the presence or absence of desiccant. Activities of cellular reagents were compared by performing PCR amplification of 10 ng TtgR plasmid templates followed by agarose gel electrophoretic analysis of expected amplicons (*). PCR reactions analyzed in each lane were performed using the following enzyme sources – Lane 1: DNA ladder, lane 2: pure Taq DNA polymerase, lane 3: 2 x 10^7 cellular reagents from 3 μL cellular reagents dried with desiccant, lane 4: 2 x 10^7 cellular reagents from 3 μL cellular reagents dried without desiccant, lane 5: 2 x 10^7 cellular reagents from 15 μL cellular reagents dried with desiccant, lane 6: 2 x 10^7 cellular reagents from 15 μL cellular reagents dried without desiccant, lane 7: 2 x 10^7 cellular reagents from 30 μL cellular reagents dried with desiccant, lane 8: 2 x 10^7 cellular reagents from 30 μL cellular reagents dried without desiccant. Representative data from three biological replicates are depicted.

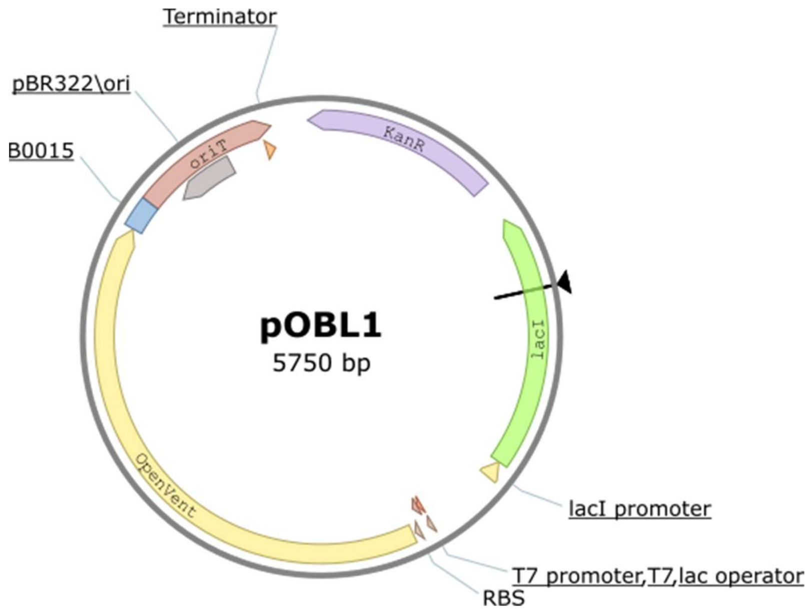

S11 Fig. pOBL1 vector map. pOBL1 sequence is also available as Genbank, FASTA, and benchling files.

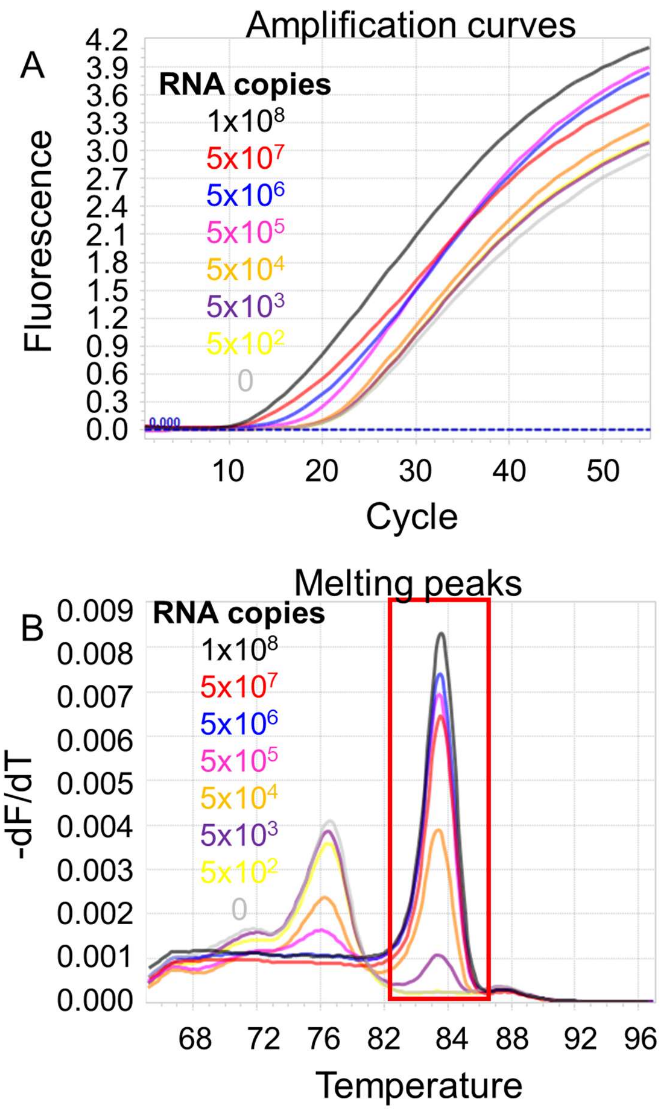

RNA copies	Average Cq	RNA copies	Average Cq
1 x 10^8	9.47 ± 0.59	5 x 10^4	17.29 ± 1.17
5 x 10^7	10.87 ± 0.21	5 x 10^3	17.84 ± 0.87
5 x 10^6	11.80 ± 0.44	5 x 10^2	18.23 ± 1.14
5 x 10^5	15.69 ± 2.14	0	18.35 ± 0.94

S12 Fig. SARS-CoV-2 N1 RT-qPCR assay using RTX polymerase evaporated cellular reagents. Panel A depicts CDC SARS-CoV-2 N1 assays measured in real-time using EvaGreen dye. Amplification curves from reactions containing 10^8 (black traces), $5x10^7$ (red traces), $5x10^6$ (blue traces), $5x10^5$ (pink traces), $5x10^4$ (orange traces), $5x10^3$ (purple traces), $5x10^2$ (yellow traces), and 0 (gray traces) copies of SARS-CoV-2 armored N RNA are shown. Panel B depicts melting peaks of amplicons determined using the 'Tm calling' analysis in the LightCycler96 software. Red box indicates melting peaks of expected amplicons. Average Cq values of triplicate experiments are tabulated.

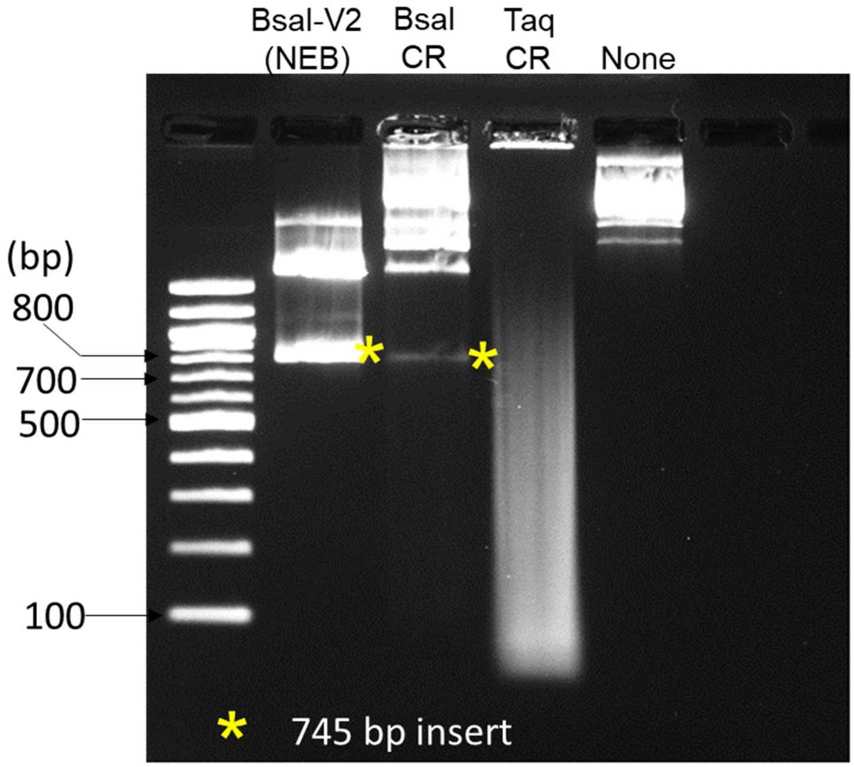

S13 Fig. Restriction digestion of plasmid DNA using evaporated *Bsa*I cellular reagents. A plasmid bearing two *Bsa*I sites flanking a 745 bp insert (asterisk) was incubated in digestion buffer supplemented with either nothing or with commercially sourced pure engineered enzyme *Bsa*I-V2, *Bsa*I evaporated cellular reagents, or *Taq* DNA polymerase evaporated cellular reagents. Resulting products analyzed by agarose gel electrophoresis are depicted. Data are representative of two biological replicates.

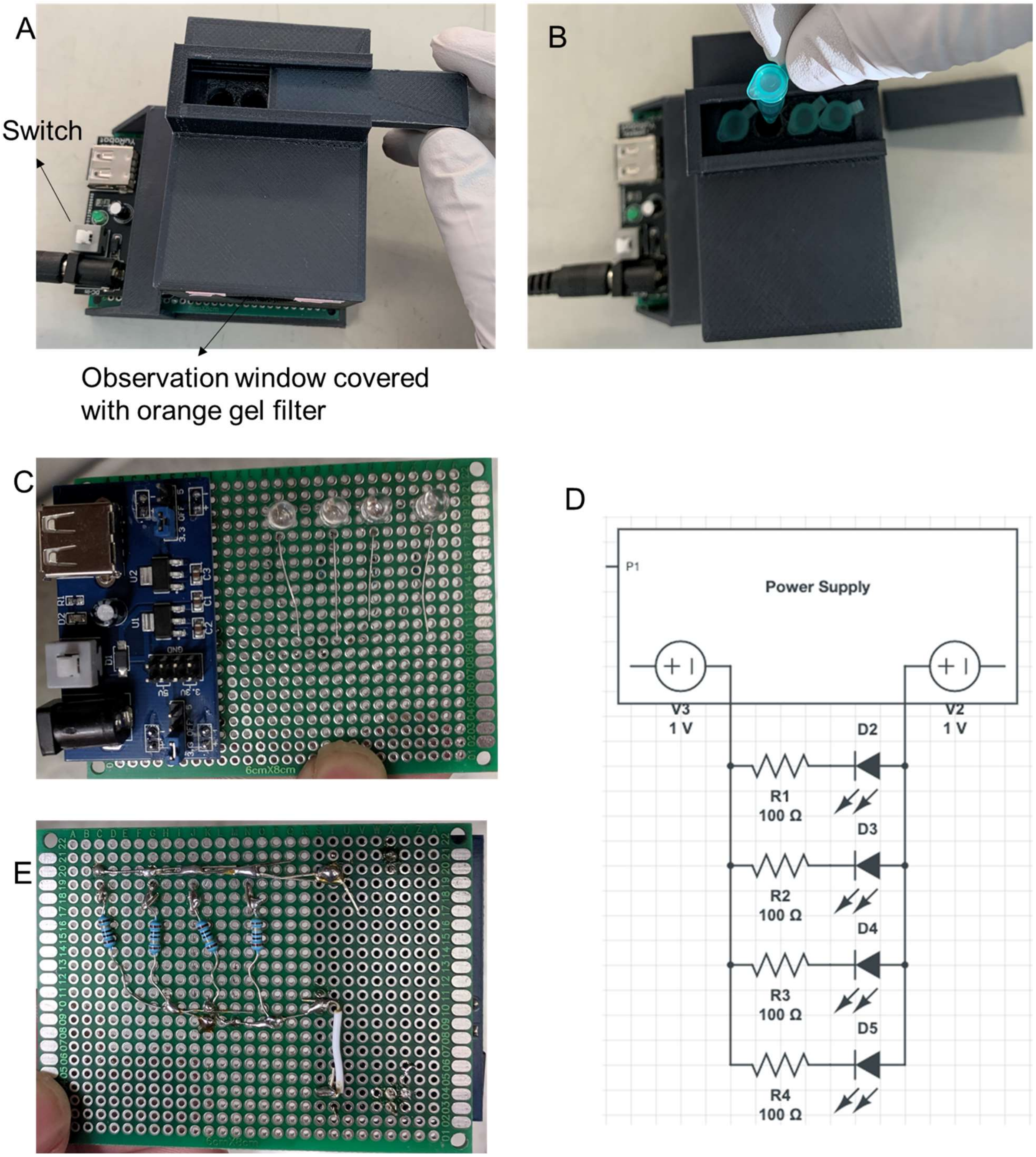

S14 Fig. Design and construction of fluorescence visualization box. (A) Visualization box with 3D printed tube chamber and LED circuit board. (B) Image showing placement of LAMP-OSD tubes inside the visualization box. (C) Top view of the LED circuit board. (D) Circuit design. (E) Bottom view of the circuit board. Detail instructions available at https://github.com/Jose-4625/LAMP-BOX-ECO/tree/master/VisBox.

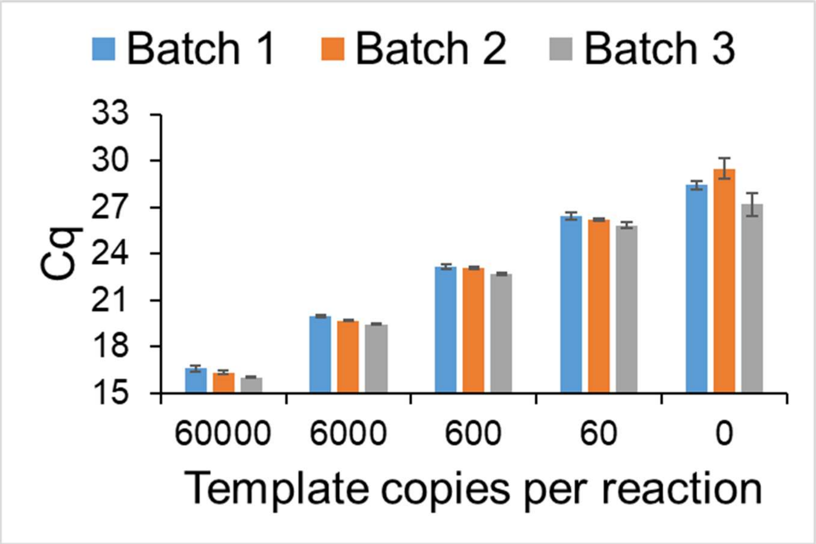

S15 Fig. Functional comparison of different batches of Taq DNA polymerase cellular reagents. Identical qPCR reactions containing indicated copies of CT16S plasmid DNA templates were performed using three independent preparations of evaporated Taq DNA polymerase cellular reagents. The average Cq values for each template copy number measured from three replicate trials performed at weekly intervals are depicted.

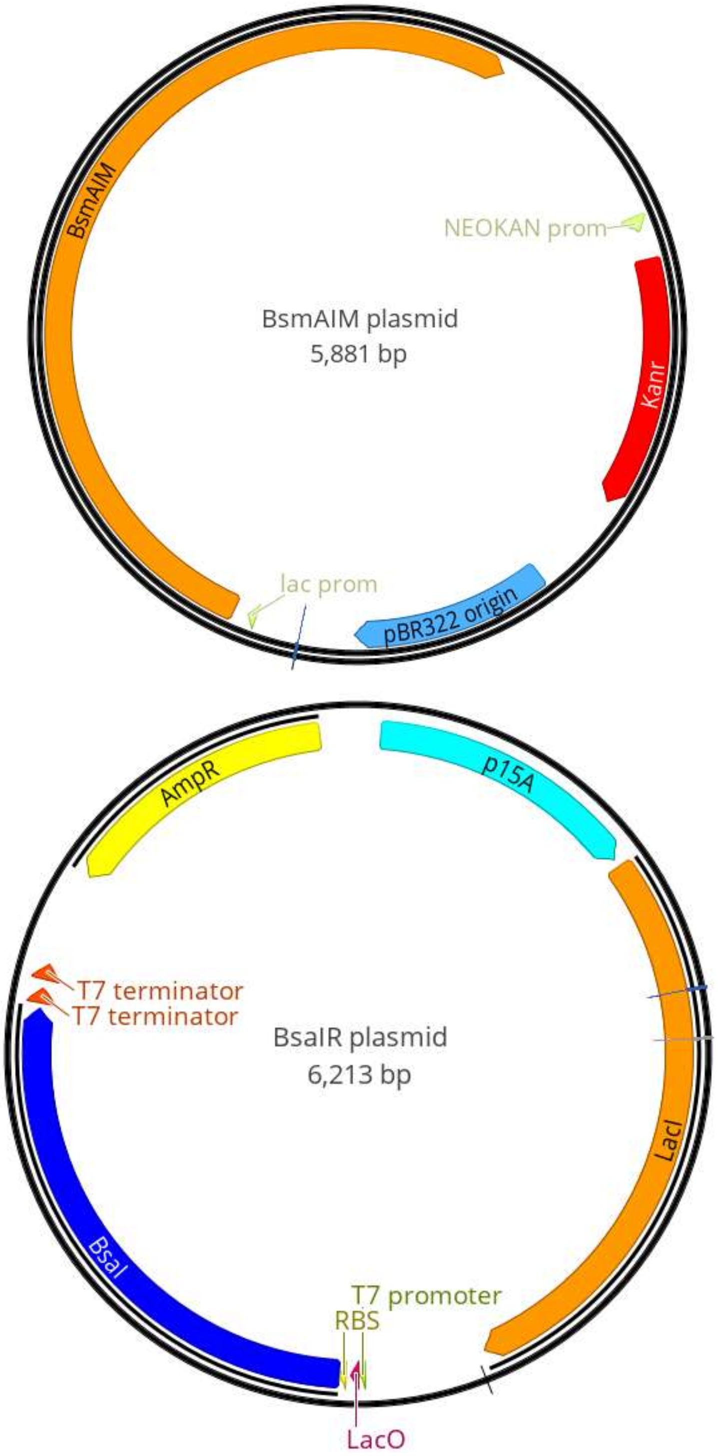

S16 Fig. *Bsm*AIM and *Bsa*IR expression construct designs.

References

1. Bhadra S, Pothukuchy A, Shroff R, Cole AW, Byrom M, Ellefson JW, et al. Cellular reagents for diagnostics and synthetic biology. Plos One. 2018; 13(8): e0201681. Epub 2018/08/16. doi: 10.1371/journal.pone.0201681. PubMed PMID: 30110361; PubMed Central PMCID: PMCPMC6093680.
2. Maranhao A, Bhadra S, Paik I, Walker D, Ellington AD. An improved and readily available version of Bst DNA Polymerase for LAMP, and applications to COVID-19 diagnostics. medRxiv. 2020: 2020.2010.2002.20203356. doi: 10.1101/2020.10.02.20203356.
3. Milligan JN, Shroff R, Garry DJ, Ellington AD. Evolution of a Thermophilic Strand-Displacing Polymerase Using High-Temperature Isothermal Compartmentalized Self-Replication. Biochemistry. 2018. Epub 2018/04/10. doi: 10.1021/acs.biochem.8b00200. PubMed PMID: 29629759.
4. Querellou J, Cambon M. Heat-stable DNA polymerase of archaeobacteria of genus pyrococcus sp. US Patent application number US6673585B1. 2004.
5. Xu MQ, Southworth MW, Mersha FB, Hornstra LJ, Perler FB. In vitro protein splicing of purified precursor and the identification of a branched intermediate. Cell. 1993; 75(7): 1371-1377. Epub 1993/12/31. doi: 10.1016/0092-8674(93)90623-x. PubMed PMID: 8269515.
6. Puigbo P, Guzman E, Romeu A, Garcia-Vallve S. OPTIMIZER: a web server for optimizing the codon usage of DNA sequences. Nucleic Acids Res. 2007; 35(Web Server issue): W126-131. Epub 2007/04/19. doi: 10.1093/nar/gkm219. PubMed PMID: 17439967; PubMed Central PMCID: PMCPMC1933141.
7. Staal J, Alci K, De Schamphelaire W, Vanhoucke M, Beyaert R. Engineering a minimal cloning vector from a pUC18 plasmid backbone with an extended multiple cloning site. BioTechniques. 2019; 66(6): 254-259. Epub 2019/05/28. doi: 10.2144/btn-2019-0014. PubMed PMID: 31124712.
8. Bailey MD, Mohamed YH. Klenow Assembly Method: Seamless cloning. 2019; Available from: https://openwetwareorg/wiki/Klenow_Assembly_Method:_Seamless_cloning.
9. McFarland J. The Nephelometer: an instrument for estimating the number of bacteria in suspensions used for calculating the opsonic index and for vaccines. JAMA. 1907; 49(14): 1176-1178. doi: 10.1001/jama.1907.25320140022001f.
10. Voigt C, Voigt CA. Synthetic Biology, Part A: Methods for Part/Device Characterization and Chassis Engineering: Elsevier Science; 2011.
11. Baldwin G, Bayer T, Dickinson R, Ellis T, Freemont PS, Kitney RI, et al. Synthetic Biology — A Primer: World Scientific; 2012.
12. Waggoner JJ, Pinsky BA. Zika Virus: Diagnostics for an Emerging Pandemic Threat. Journal of clinical microbiology. 2016; 54(4): 860-867. Epub 2016/02/19. doi: 10.1128/JCM.00279-16. PubMed PMID: 26888897; PubMed Central PMCID: PMCPMC4809954.
13. https://www.minipcr.com/product/sickle-cell-genetics-lab/.
