# Supplementary material for: Producing molecular biology reagents without purification
Source: PLoS One. 2021 Jun 1;16(6):e0252507. doi: 10.1371/journal.pone.0252507 (PMC8168896; doi:10.1371/journal.pone.0252507)

Producing molecular biology reagents without purification

Sanchita Bhadra[1,2,*], Vylan Nguyen[3], Jose-Angel Torres[3], Shaunak Kar[1,2], Stéphane Fadanka[6], Chiara Gandini[4], Harry Akligoh[5], Inyup Paik[1,2], Andre C. Maranhao[1,2], Jenny Molloy[4], Andrew D. Ellington[1,2,*]

Raw image files

Fig 2. All images were captured using a ChemiDoc camera (Bio-Rad, Hercules, CA, USA). Activities of cellular reagents were compared by performing 20 cycle PCR amplification of 10 ng CT16S plasmid templates using either 3 µL of rehydrated evaporated or lyophilized cellular reagents prepared without solid support (‘Liquid’) or a single 3 mm paper disc containing evaporated cellular reagents added into the PCR reaction mix (PTR5 and GF800: two types of glass fiber conjugate pad paper; 691, 693, 161: three types of glass fiber filter paper).

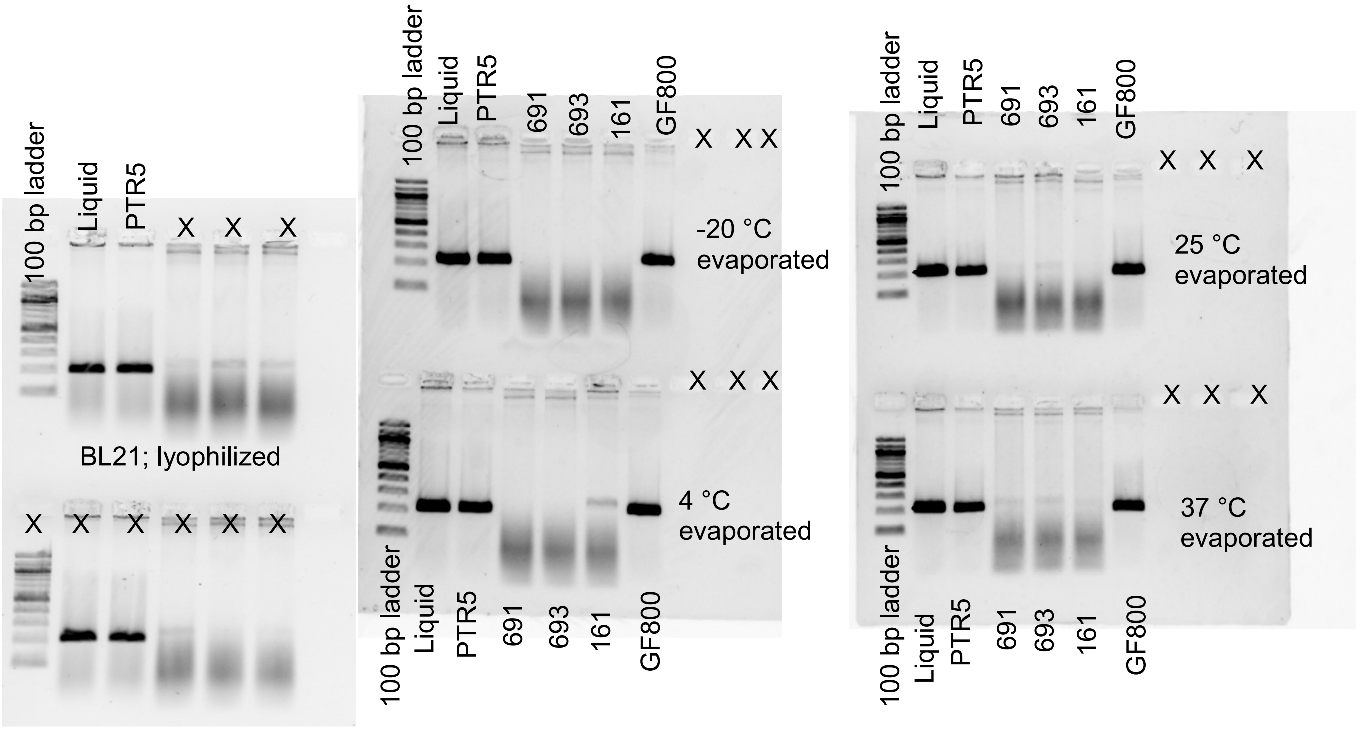

Fig.5 All images are taken using a smartphone camera. The transilluminator used was the MiniPCR blueGel™ electrophoresis system.

(A) Left panel.

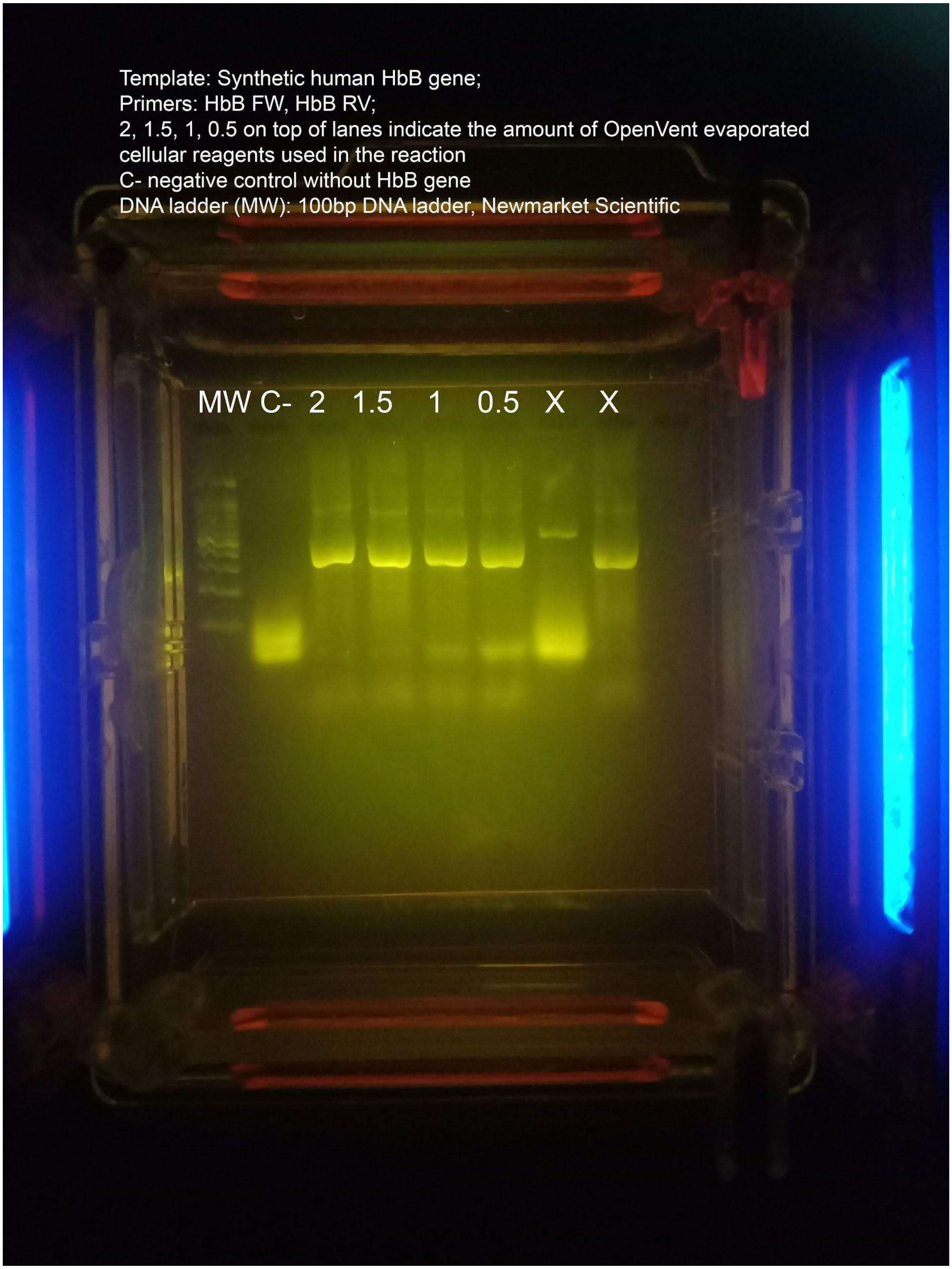

(A) Right panel.

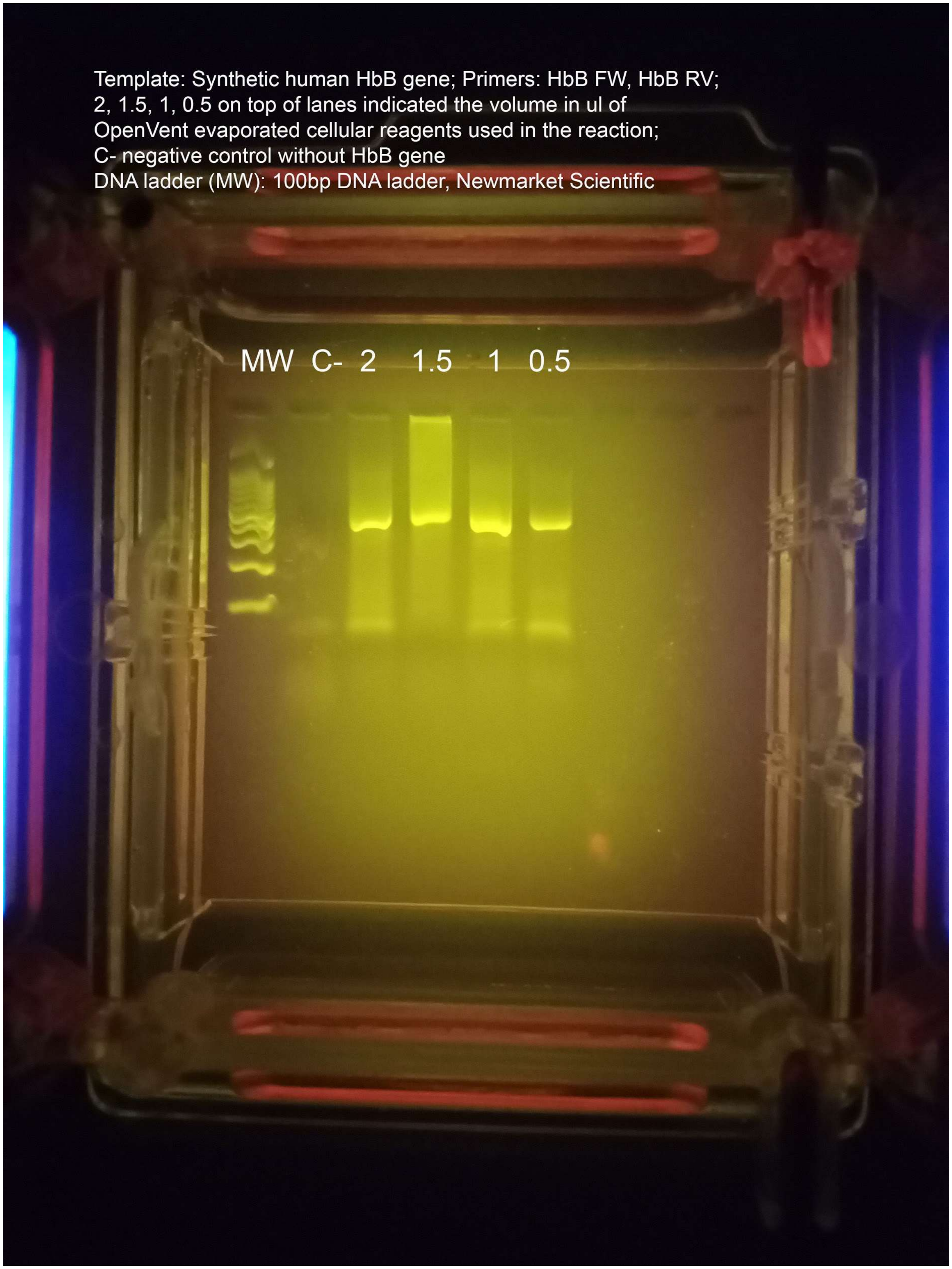

(B) Left panel.

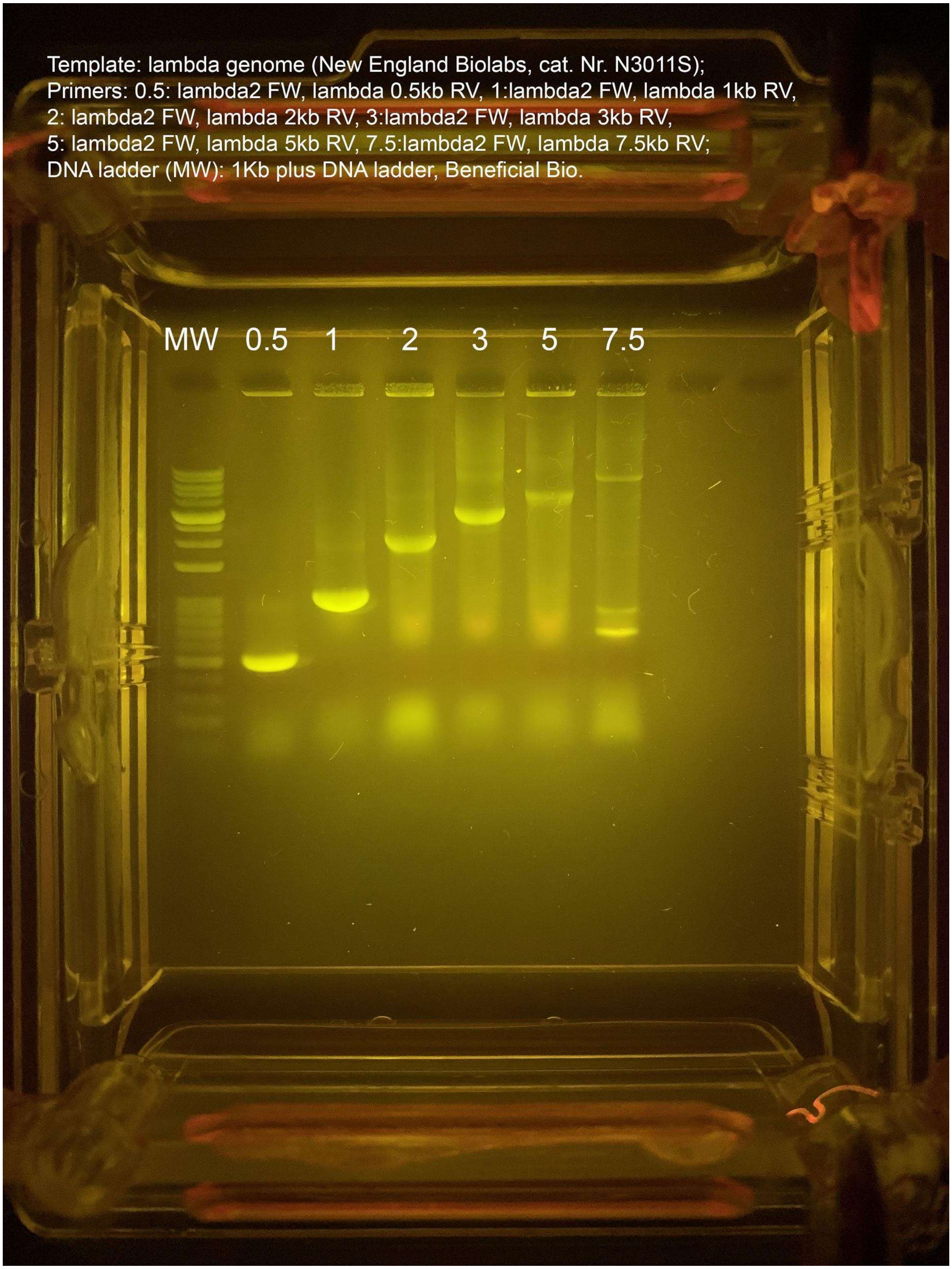

Template: lambda genome (New England Biolabs, cat. Nr. N3011S);
Primers: 0.5: lambda2 FW, lambda 0.5kb RV, 1:lambda2 FW, lambda 1kb RV,
2: lambda2 FW, lambda 2kb RV, 3:lambda2 FW, lambda 3kb RV,
5: lambda2 FW, lambda 5kb RV, 7.5:lambda2 FW, lambda 7.5kb RV;
DNA ladder (MW): 1Kb plus DNA ladder, Beneficial Bio.
MW
0.5
1
2
3
5
7.5

(B) Right panel.

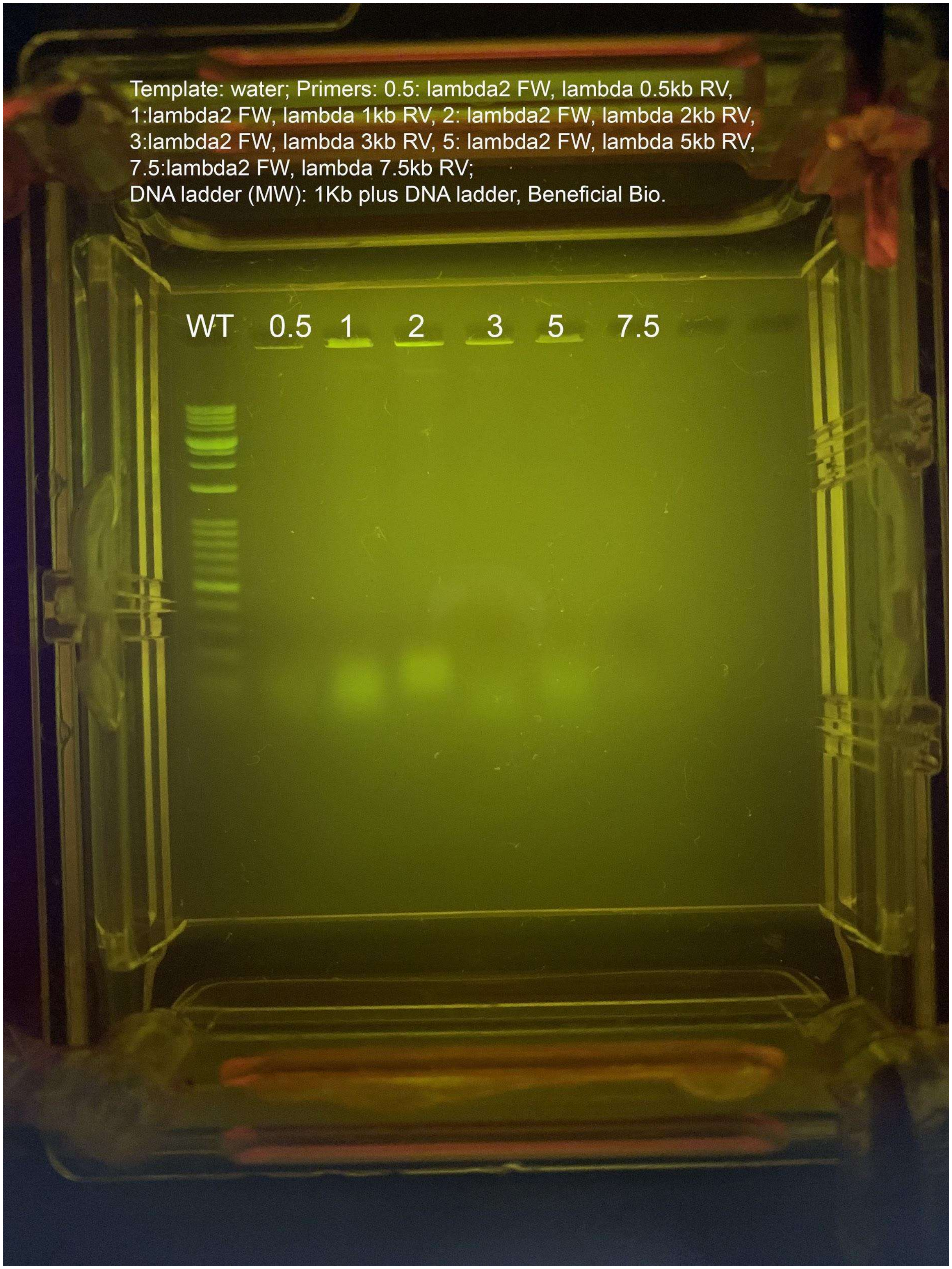

Fig 6. Detection of SARS-CoV-2 using Br512 polymerase evaporated cellular reagents and multiplex RT-LAMP-OSD assays. Multiplex RT-LAMP-OSD assays for viral N and ORF1AB genes were operated using only Br512 polymerase cellular reagents and indicated amounts of inactivated SARS-CoV-2 virions.

Images of endpoint OSD fluorescence were taken using a ChemiDoc camera (Bio-Rad, Hercules, CA, USA). Lateral flow dipsticks were imaged using a smartphone camera.

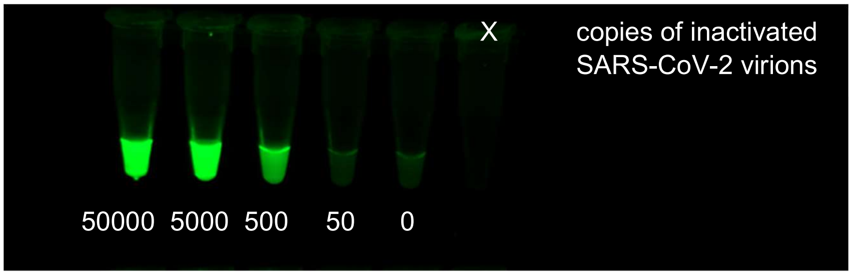

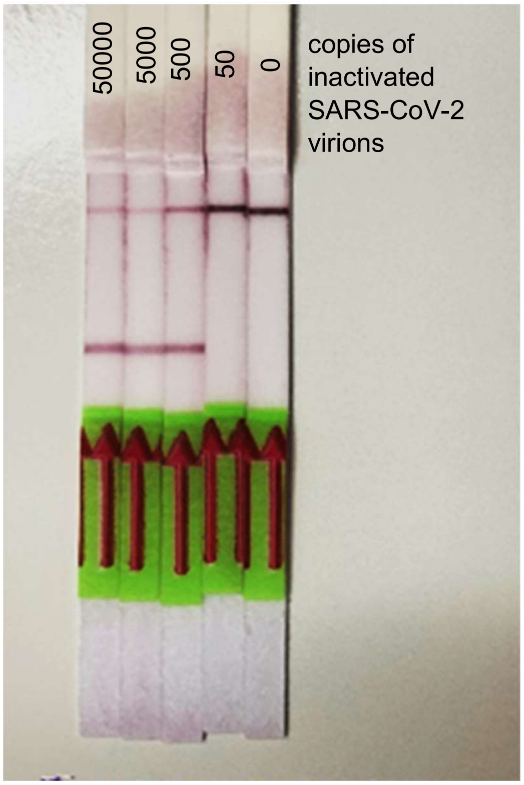

Fig 7. DNA assembly using evaporated cellular reagents. Pure enzymes (NEB) or evaporated cellular reagents (CR) for BsaI, T7 DNA ligase, and T4 DNA ligase were used to assemble a PCR product comprising the coding sequence of pink chromoprotein FP595 flanked by two BsaI restriction sites into a chloramphenicol resistant plasmid bearing two *Bsa*I restriction sites downstream of a constitutive lac promoter.

Image of bacterial pellets centrifuged from overnight cultures was taken with a smartphone camera.

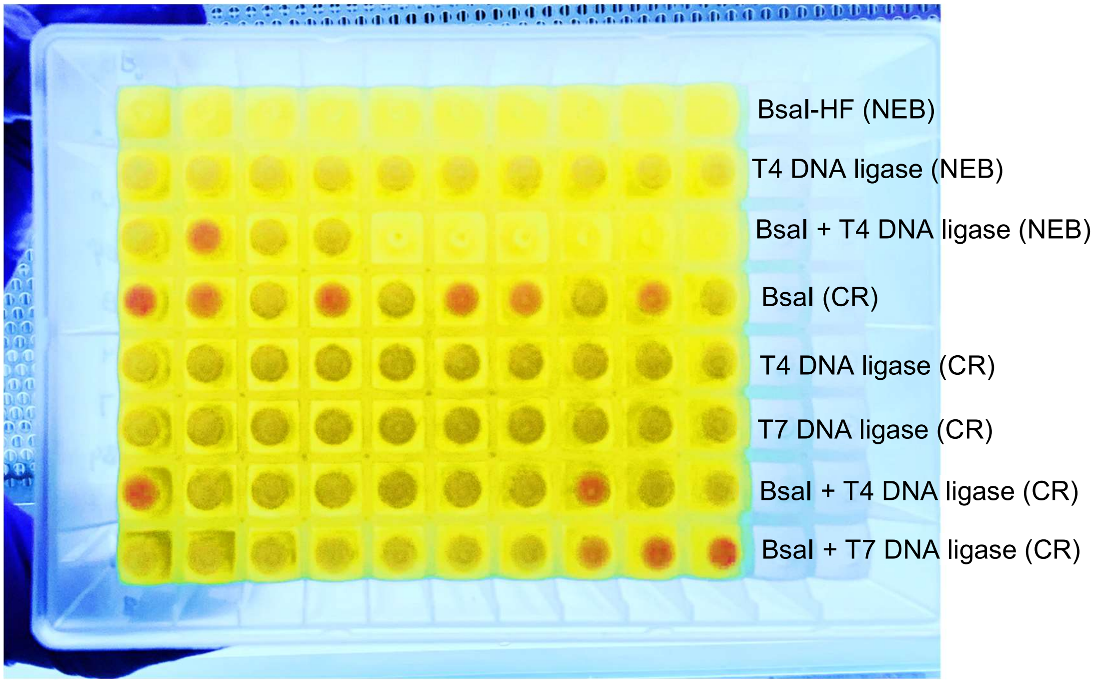

Fig 8. PCR-based molecular testing kit using Taq DNA polymerase evaporated cellular reagents. (B) HF183 PCR performed using evaporated Taq cellular reagents and analyzed by agarose gel electrophoresis. M: 100 bp molecular weight ladder; (-): no template; (+): with 10 ng plasmid template. (C) Indicated amounts of HF183 plasmid templates were PCR amplified using pure Taq DNA polymerase or Taq DNA polymerase evaporated cellular reagents and analyzed using lateral flow dipsticks.

Image of agarose gel was taken using a ChemiDoc camera (Bio-Rad, Hercules, CA, USA). Lateral flow dipsticks were imaged using a smartphone camera.

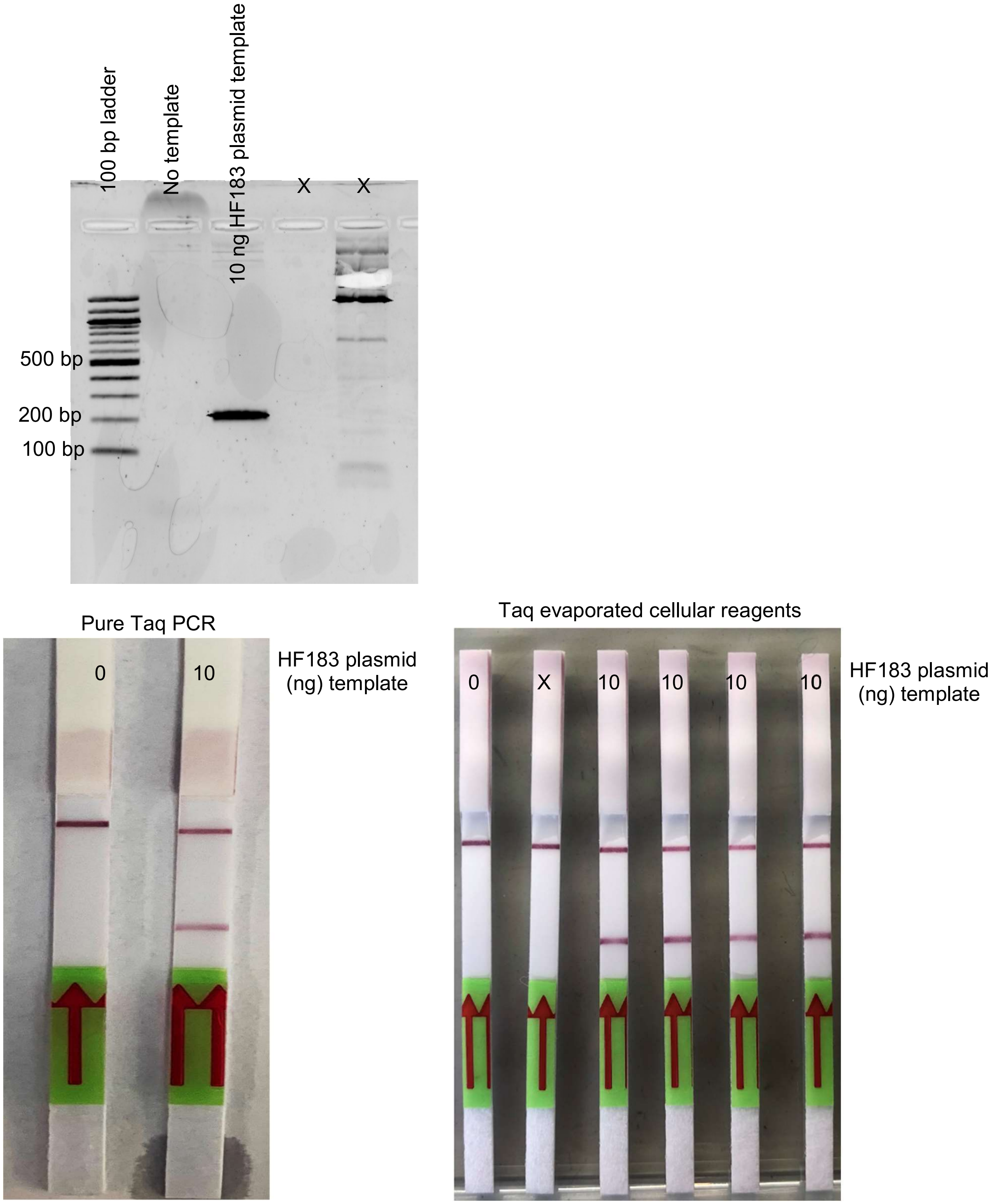

Fig 9. LAMP-OSD based molecular testing kit using Bst-LF evaporated cellular reagents. 80,000 (+) or 0 (--) copies of HF183 plasmid templates were amplified using HF183 LAMP-OSD assays operated with either Bst-LF cellular reagents (CR) or with pure Bst 2.0 enzymes. Endpoint image of OSD fluorescence was taken using a ChemiDoc camera (Bio-Rad, Hercules, CA, USA). Lateral flow dipsticks inside Ustar cartridges were imaged using a smartphone camera.

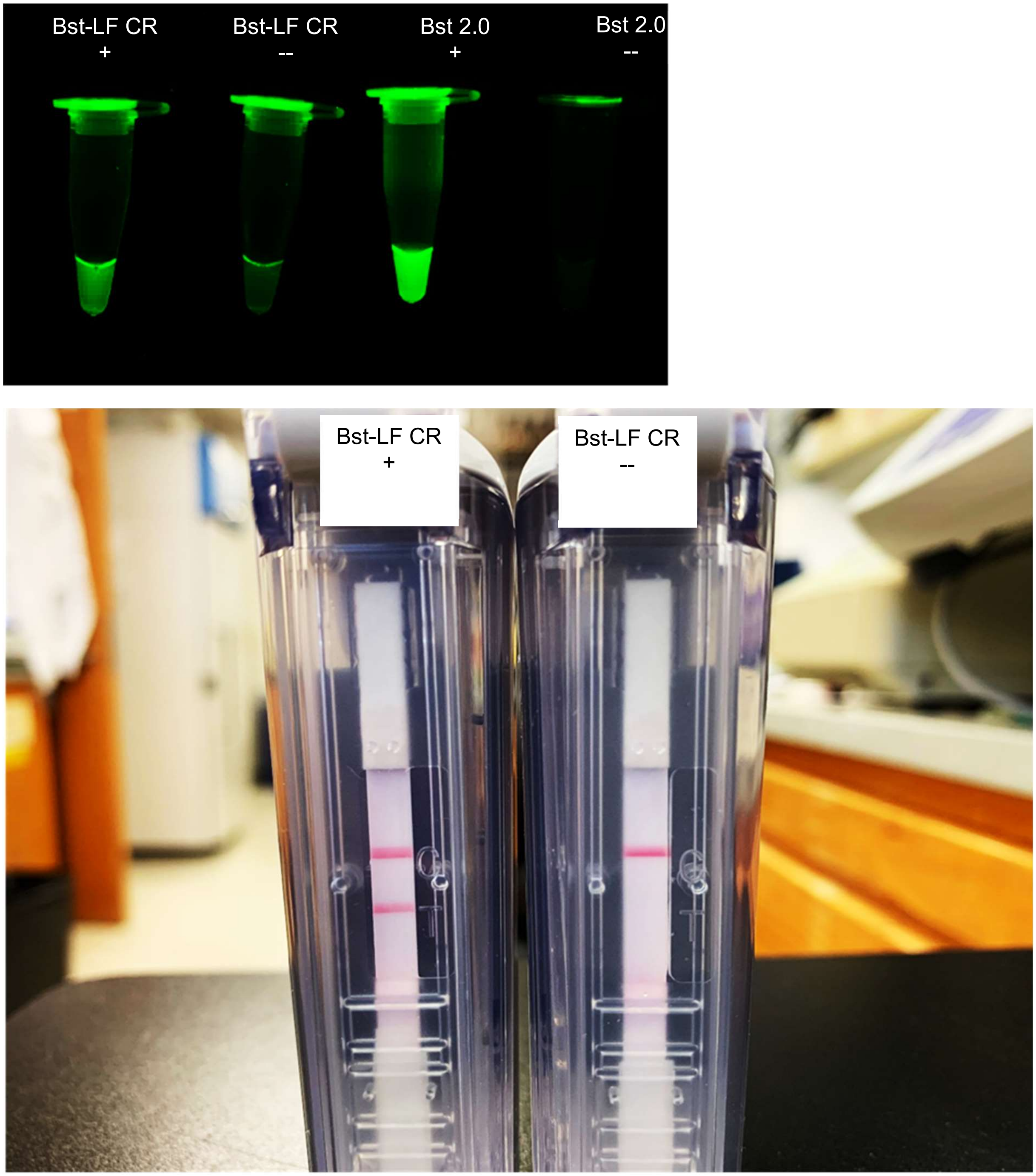

S4 Fig. The raw images are taken using a smartphone camera

Panel B

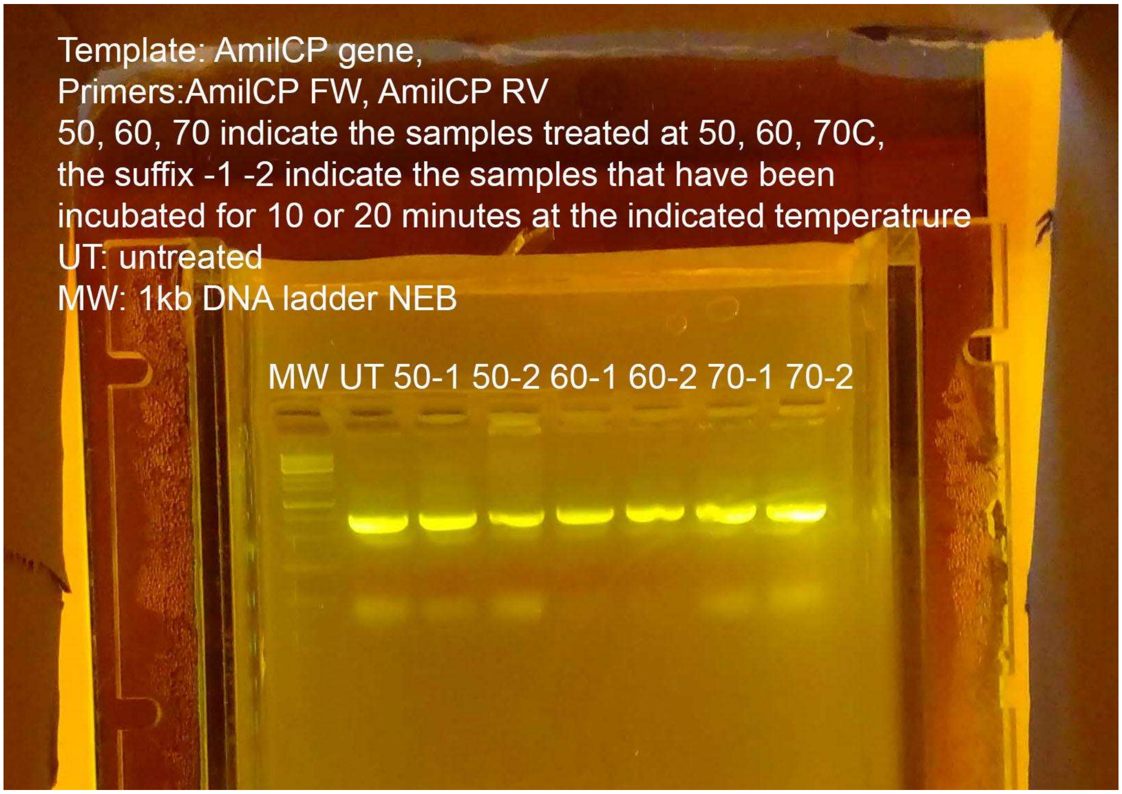

Panel C

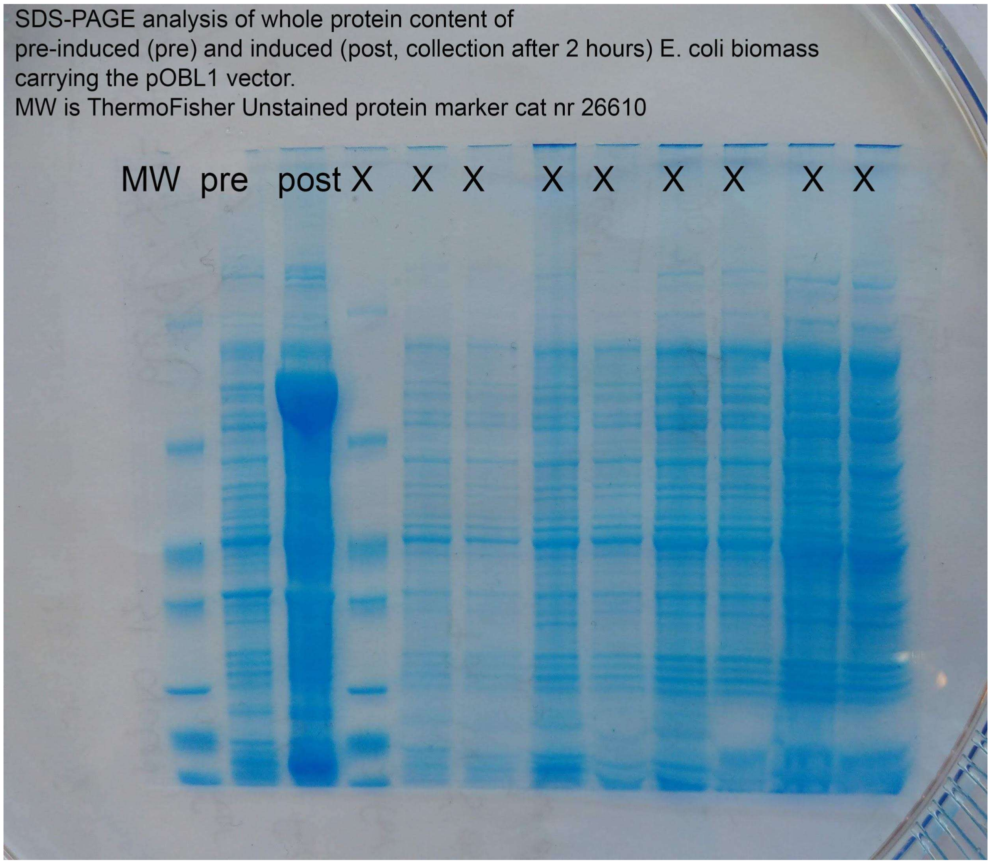

S6 Fig. All images are taken using a smartphone camera. The transilluminator used was the MiniPCR blueGel™ electrophoresis system.

A, left panel

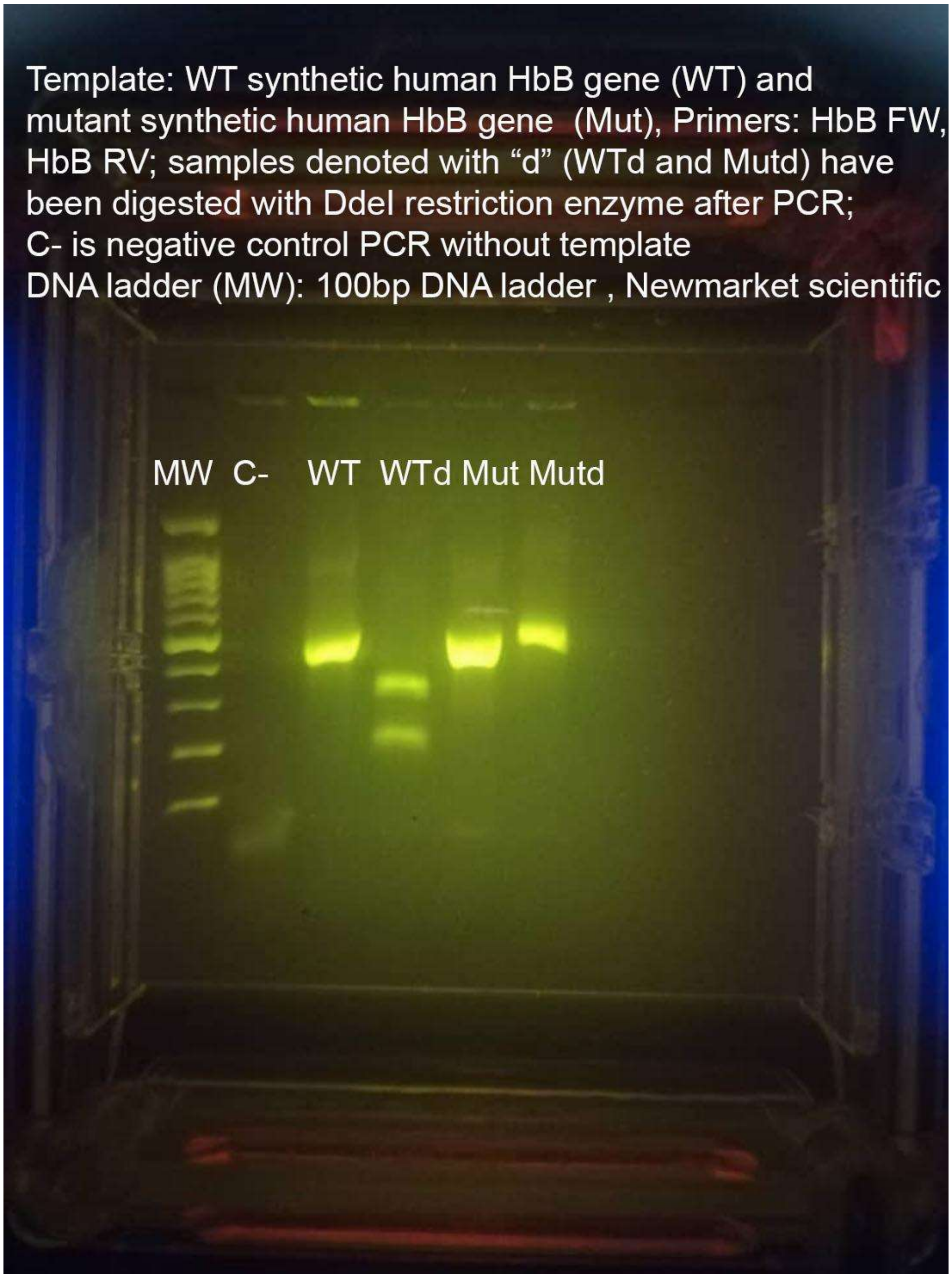

A, right panel

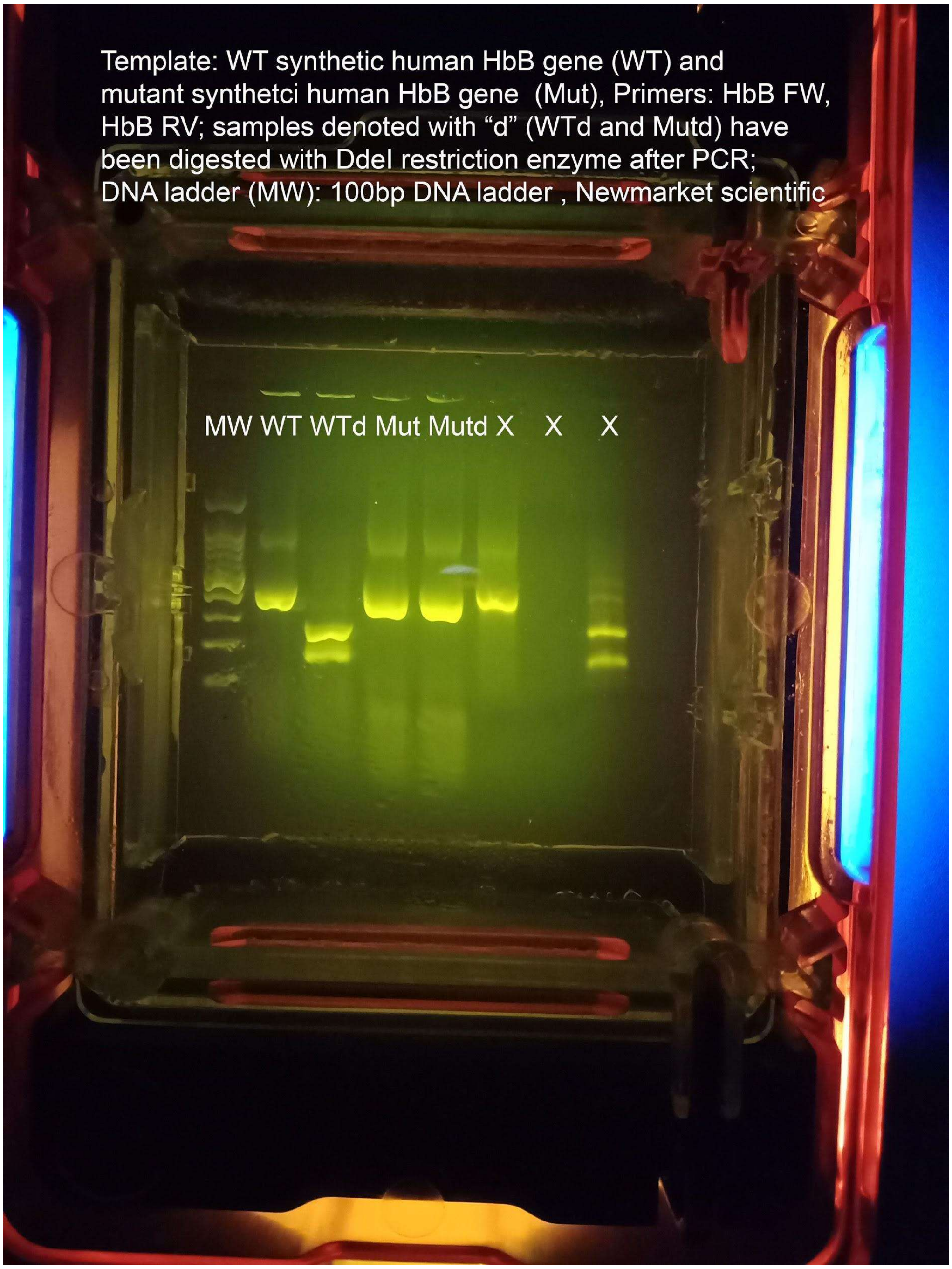

B, left panel 4 months

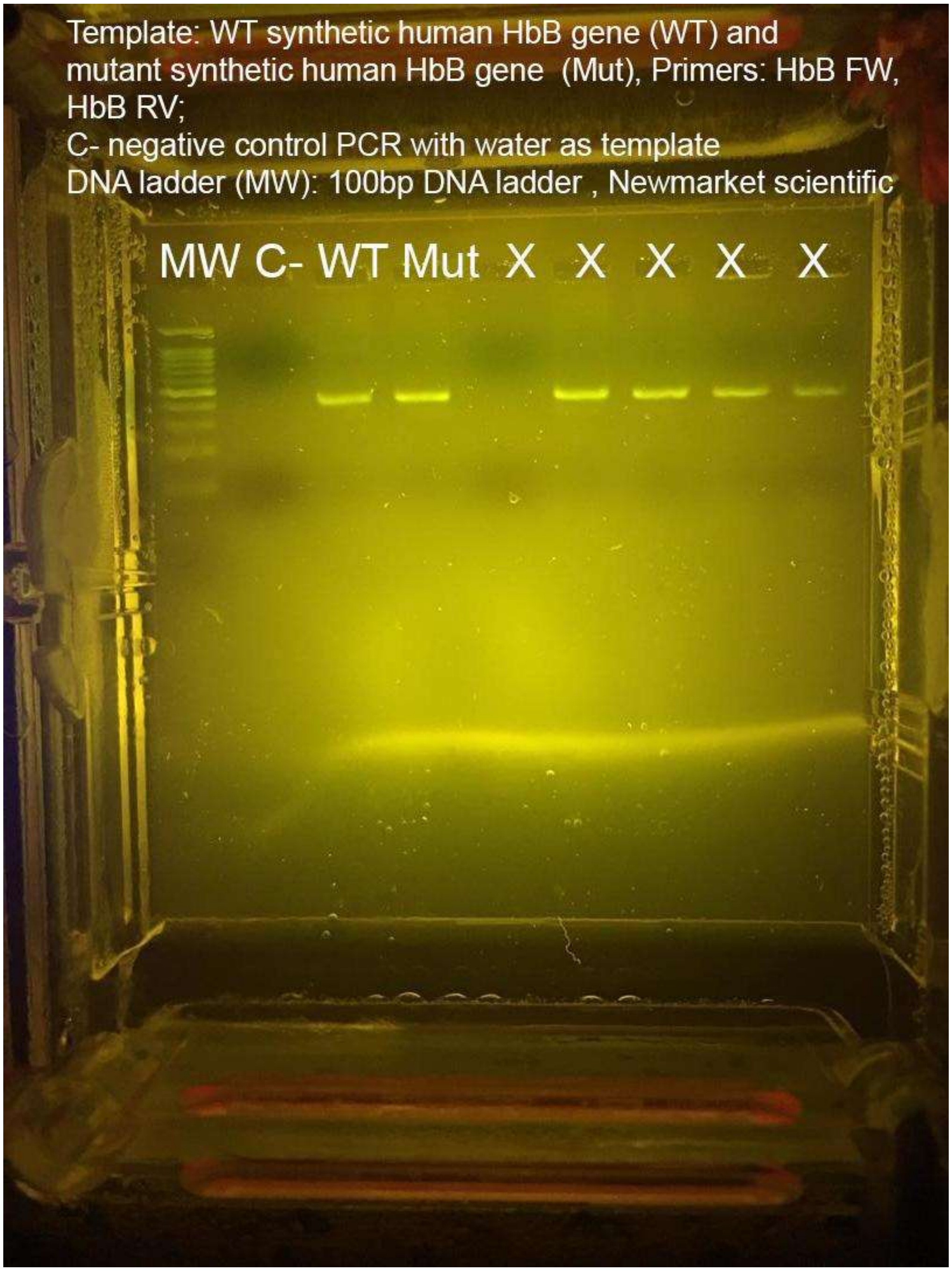

B, left panel 6 months

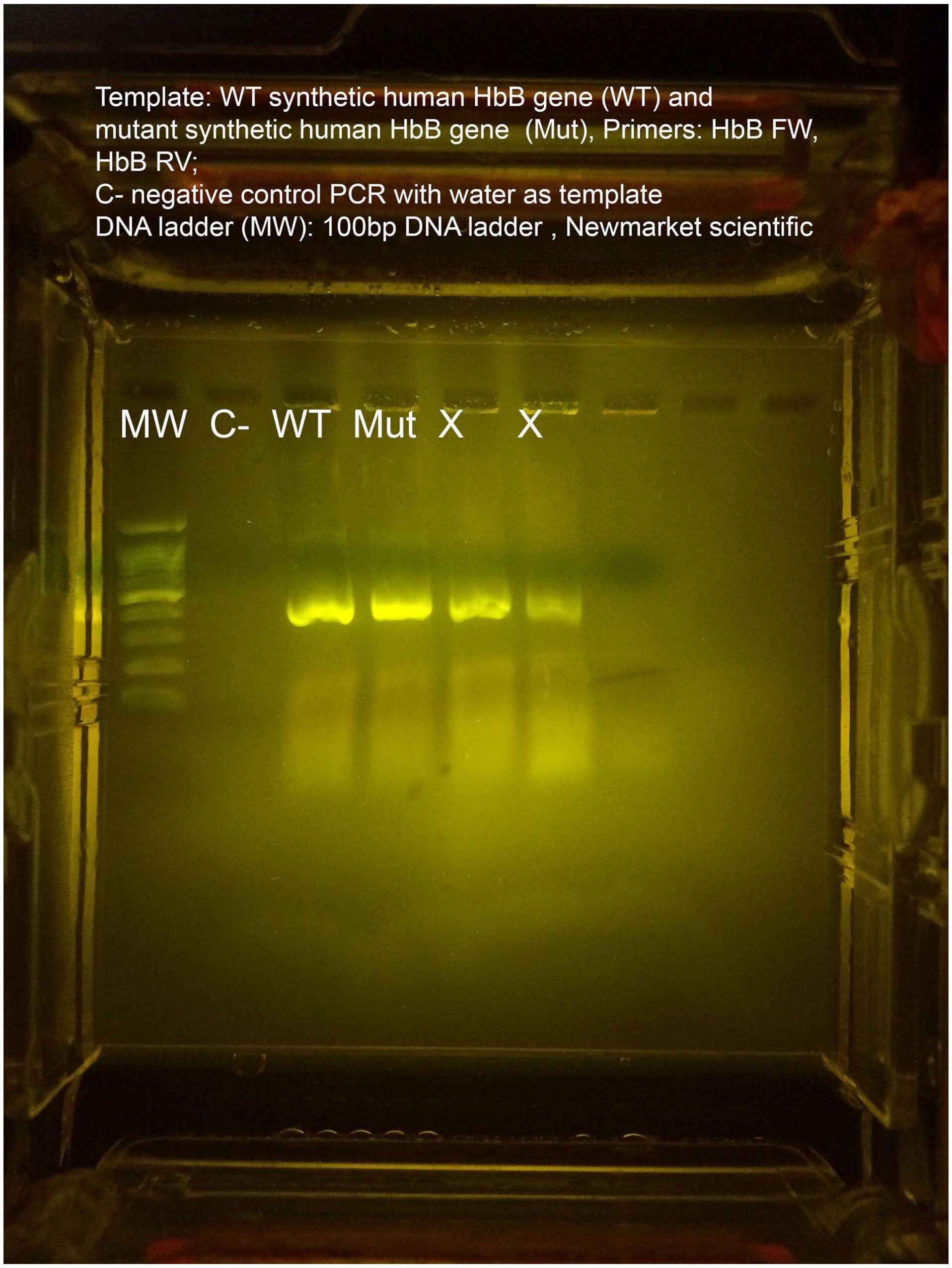

B, right panel 4 months

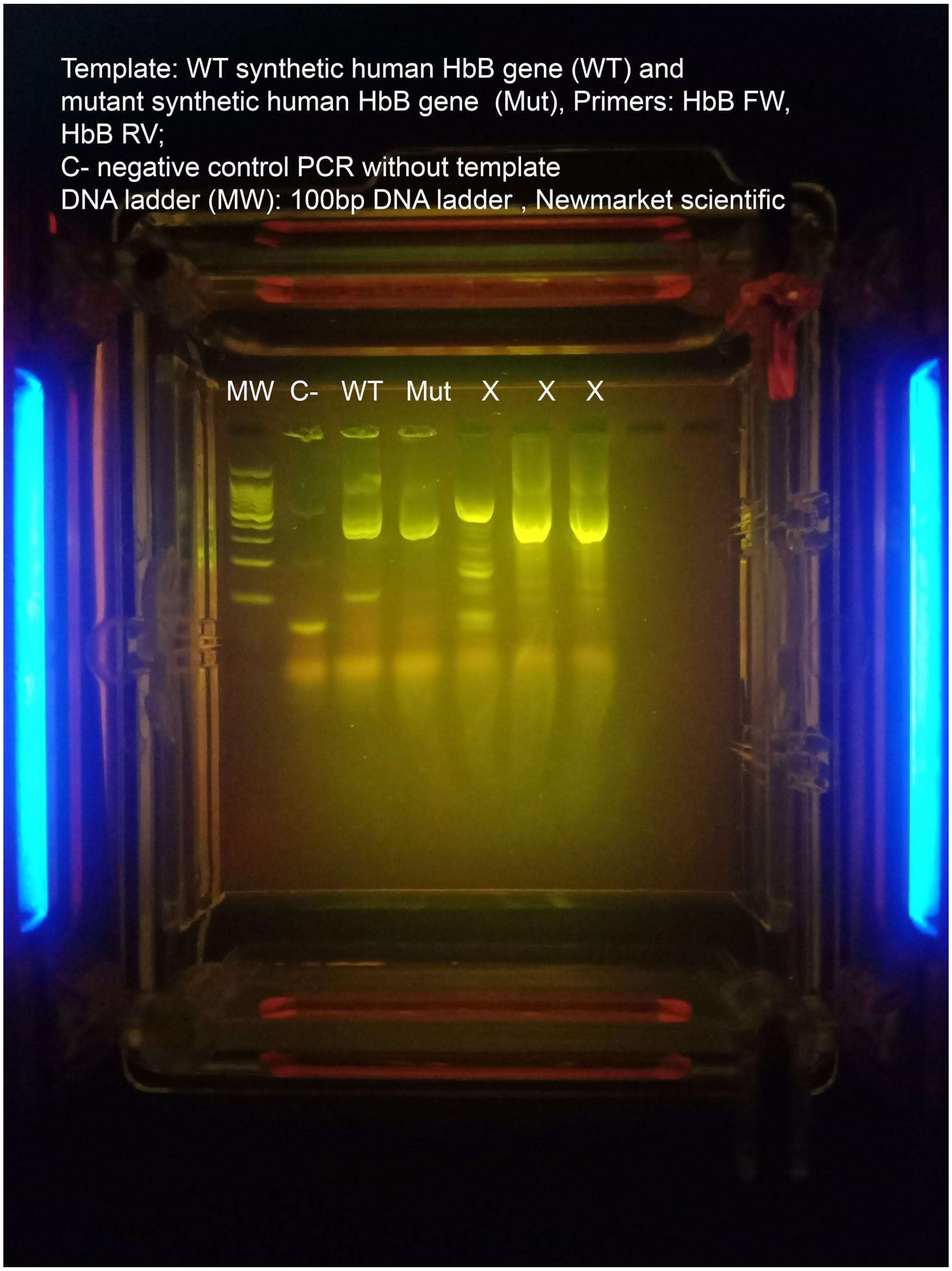

B, right panel 11 months

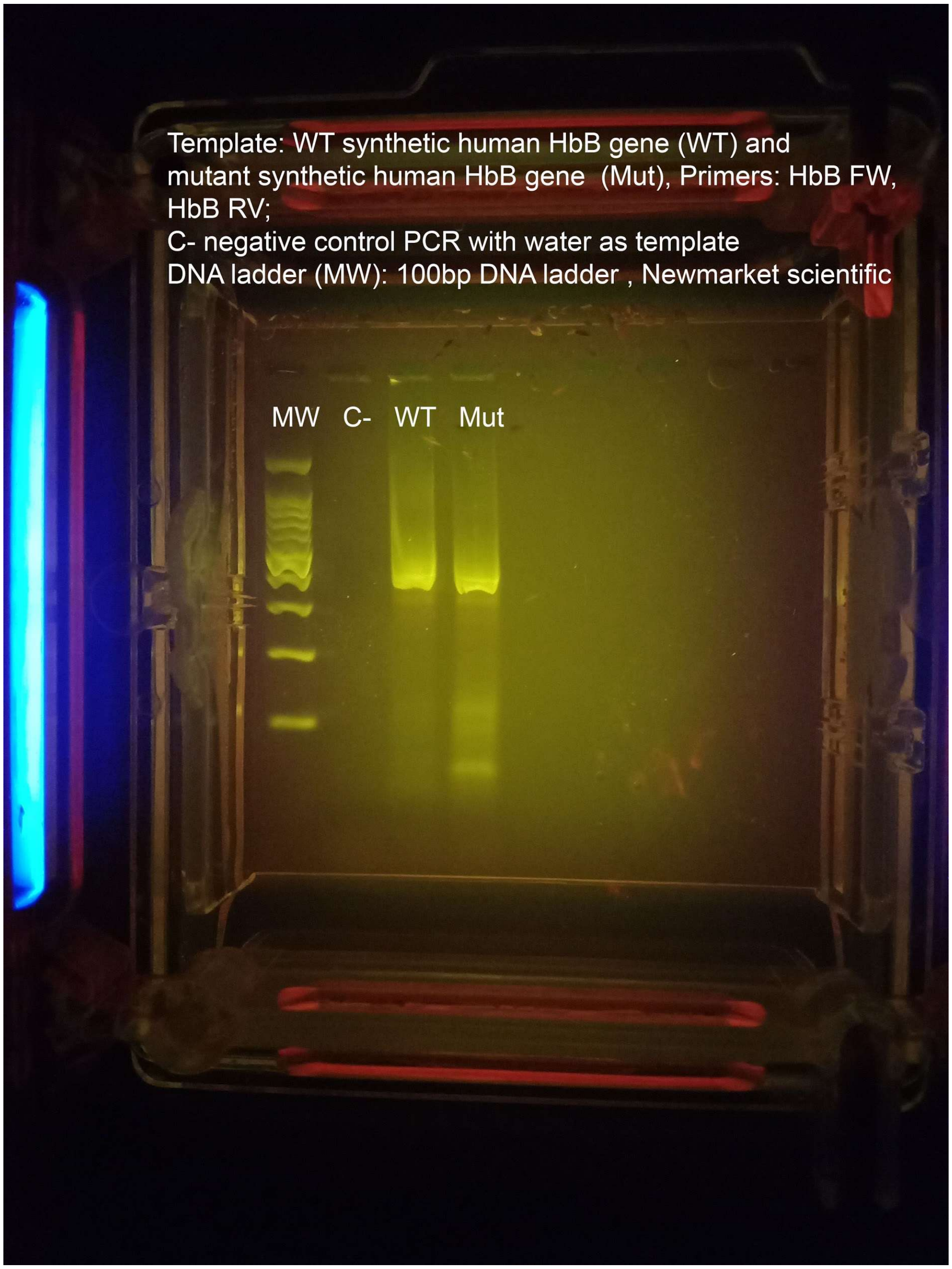

S7 Fig. Comparison of Taq cellular reagents lyophilized without solid support ('None') or lyophilized on glass fiber ('GF-PTR5'), cellulose ('CL'), or polyethersulfone ('PES') paper discs. (**A**) Activities of cellular reagents were compared by performing 20 cycle PCR amplification of CT16S templates (lanes labeled with '+') by adding either 3 µL of rehydrated cellular reagents prepared without solid support or a single 3 mm paper disc containing lyophilized cellular reagents into the PCR reaction mix. Duplicate reactions lacking templates (lanes labeled with '-') were used as negative controls. PCR reactions performed using commercially obtained pure Taq enzyme served as positive controls. (**C**) Taq DNA polymerase cellular reagents prepared by evaporation on PTR5 glass fiber paper discs stuck on laminating plastic (left panel) tested by directly adding a punched out piece of the plastic bearing one cellular reagent disc into a PCR reaction. Agarose gel electrophoretic analysis of PCR amplification performed in the presence (+) or absence (-) of templates is depicted. Reactions performed using cellular reagents evaporated without solid support served as control. Images of agarose gels were taken using a SynGene Gel doc camera (Cambridge, UK).

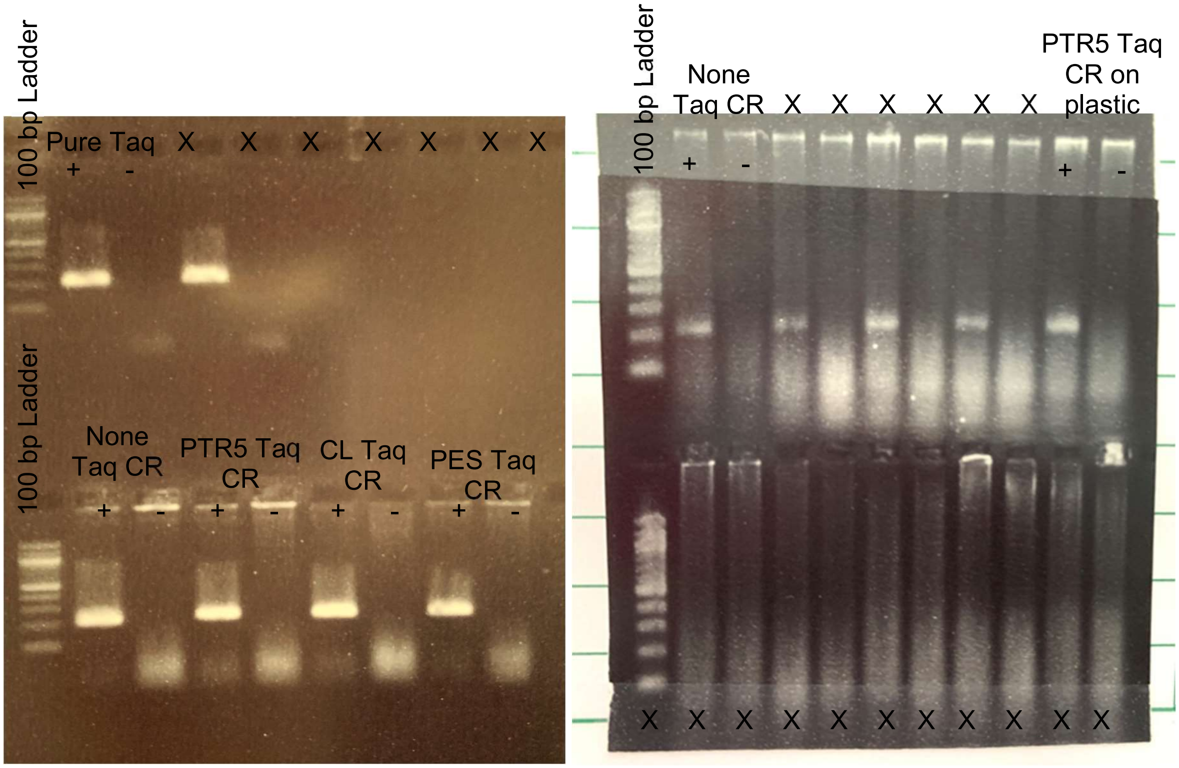

S8 Fig. Storage stability of evaporated Taq DNA polymerase cellular reagents. Two batches of Taq DNA polymerase cellular reagents evaporated at the indicated temperature with (P) or without (N) solid support were tested after 2-2.5 months of preparation by performing 20 cycle PCR reactions with or without CT16S plasmid templates. Agarose gels were imaged using a ChemiDoc camera (Bio-Rad).

37	37	25	25	4	4	-20	-20	37	37	Temperature (°C)
N	P	N	P	N	P	N	P	N	P	Solid support

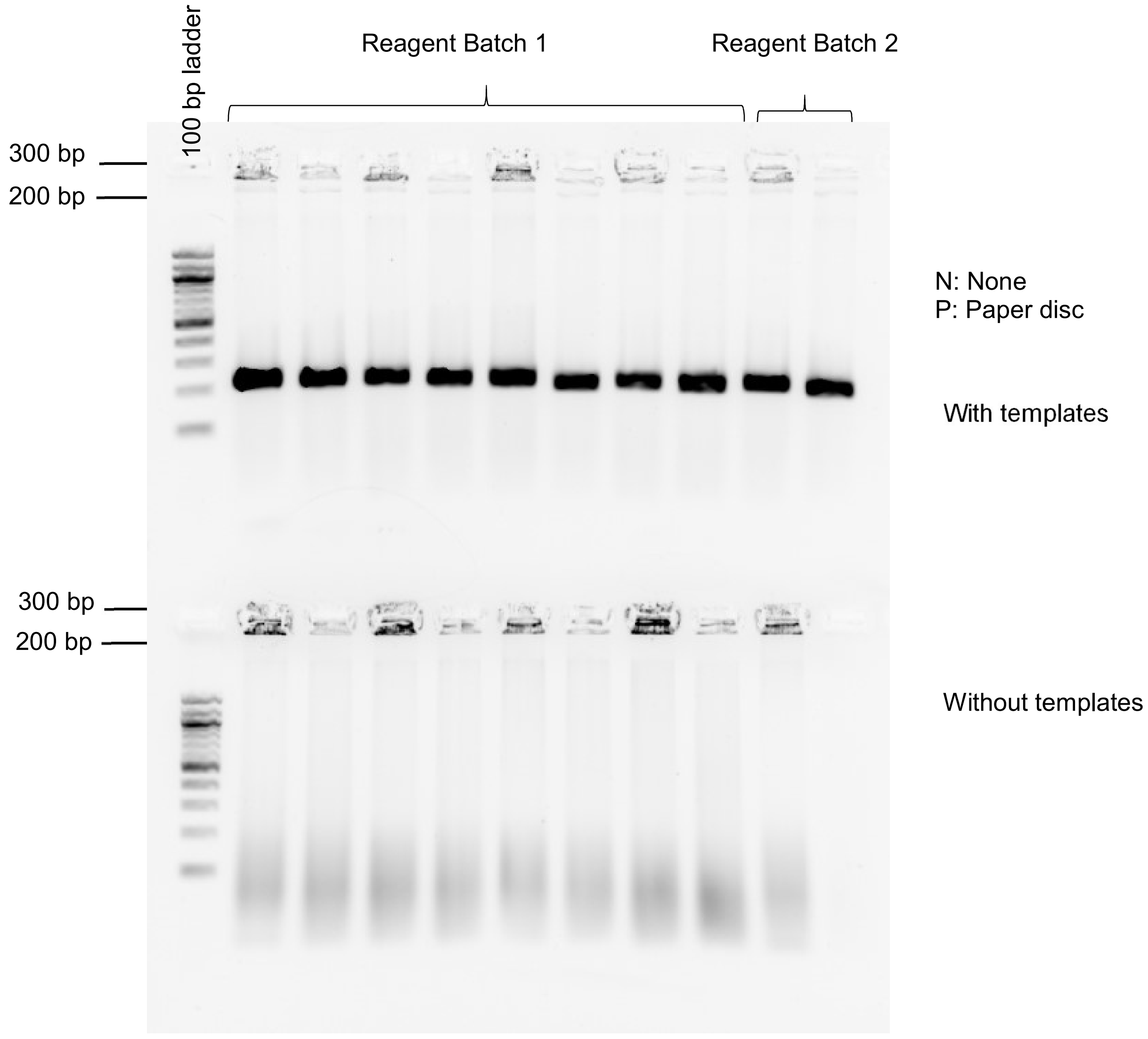

S10 Fig. Endpoint PCR using Taq DNA polymerase cellular reagents dried in the presence or absence of desiccant. Activities of cellular reagents were compared by performing PCR amplification of 10 ng TtgR plasmid templates followed by agarose gel electrophoretic analysis of expected amplicons (*). PCR reactions analyzed in each lane were performed using the following enzyme sources – Lane 1: DNA ladder, lane 2: pure Taq DNA polymerase, lane 3: 2 x 10^7 cellular reagents from 3 µL cellular reagents dried with desiccant, lane 4: 2 x 10^7 cellular reagents from 3 µL cellular reagents dried without desiccant, lane 5: 2 x 10^7 cellular reagents from 15 µL cellular reagents dried with desiccant, lane 6: 2 x 10^7 cellular reagents from 15 µL cellular reagents dried without desiccant, lane 7: 2 x 10^7 cellular reagents from 30 µL cellular reagents dried with desiccant, lane 8: 2 x 10^7 cellular reagents from 30 µL cellular reagents dried without desiccant. Agarose gel was imaged using a ChemiDoc camera (Bio-Rad).

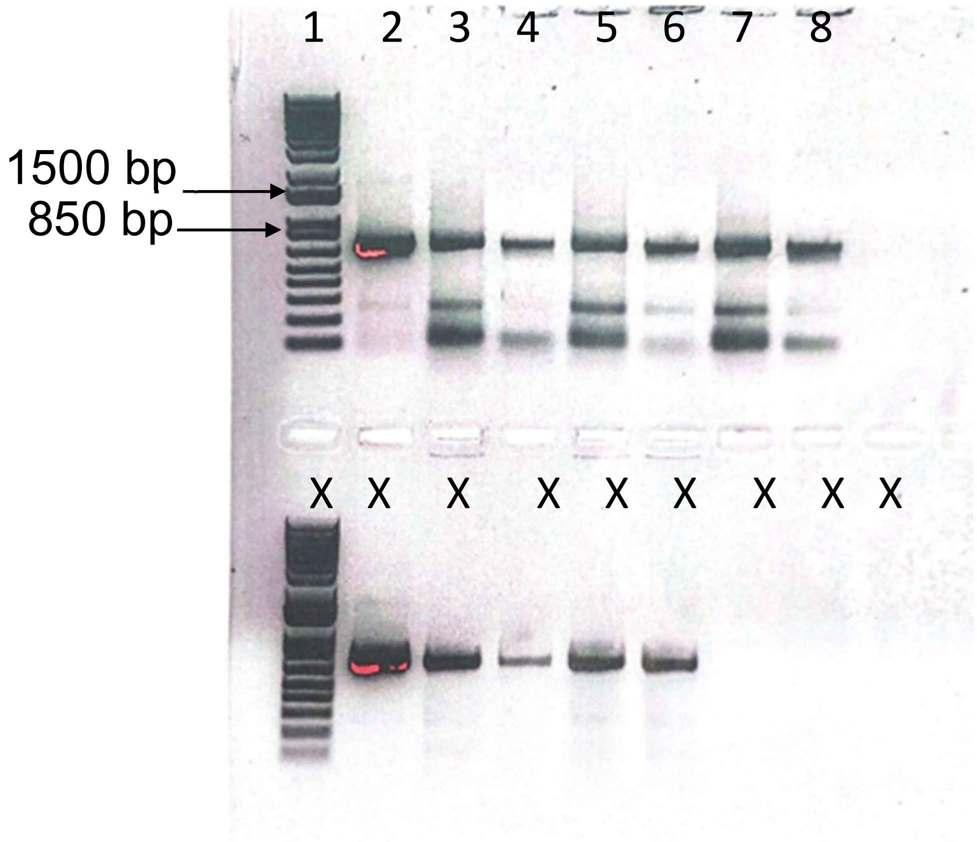

S13 Fig. Restriction digestion of plasmid DNA using evaporated *Bsa*I cellular reagents. A plasmid bearing two *Bsa*I sites flanking a 745 bp insert (asterisk) was incubated in digestion buffer supplemented with either nothing or with commercially sourced pure engineered enzyme *Bsa*I-V2, *Bsa*I evaporated cellular reagents, or *Taq* DNA polymerase evaporated cellular reagents. Agarose gel was imaged using a ChemiDoc camera (Bio-Rad).

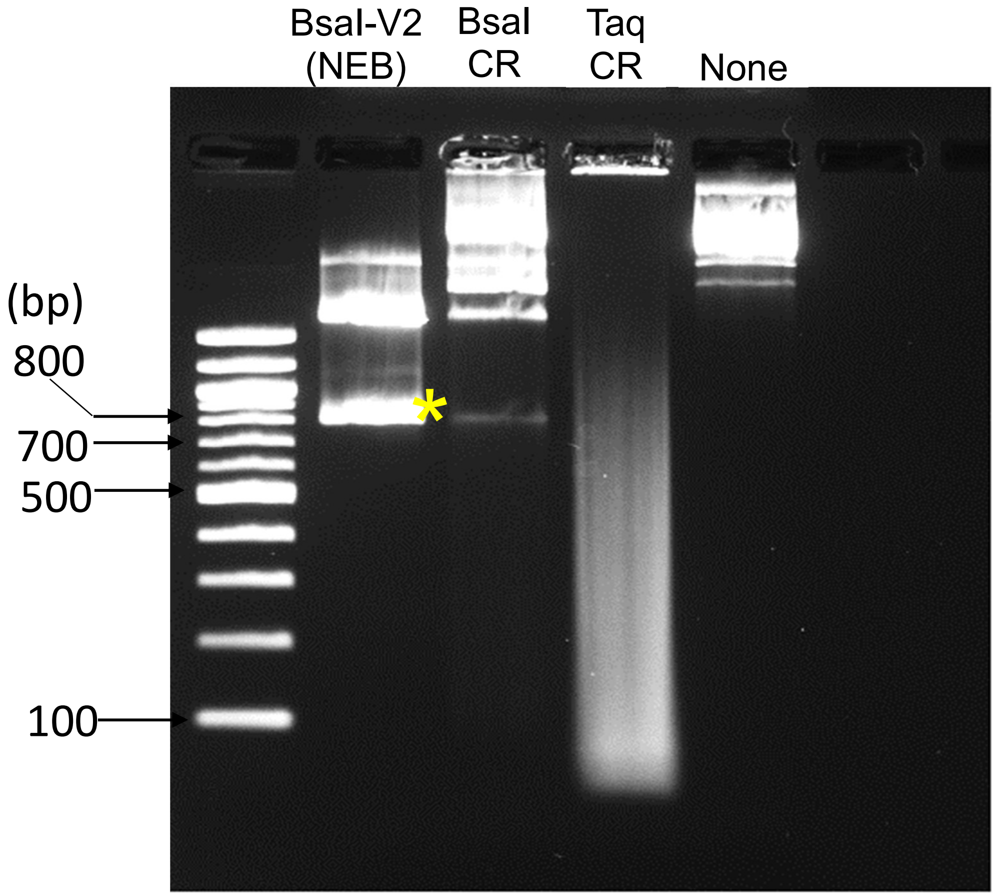

Supplement: S1 Raw images — (PDF) [file pone.0252507.s002.pdf]
